# Gold(I) α‐Trifluoromethyl Carbenes: Synthesis, Characterization and Reactivity Studies

**DOI:** 10.1002/anie.202204781

**Published:** 2022-05-03

**Authors:** Mathilde Rigoulet, David Vesseur, Karinne Miqueu, Didier Bourissou

**Affiliations:** ^1^ CNRS/Université Paul Sabatier, Laboratoire Hétérochimie Fondamentale et Appliquée (LHFA, UMR 5069) 118 Route de Narbonne 31062 Toulouse Cedex 09 France; ^2^ CNRS/Université de Pau et des Pays de l'Adour, E2S-UPPA Institut des Sciences Analytiques et de Physico-Chimie pour l'Environnement et les Matériaux (IPREM, UMR 5254) Hélioparc, 2 Avenue du Président Angot 64053 Pau Cedex 09 France

**Keywords:** Bonding, Carbene Complexes, Diazo Compounds, Gold, Trifluoromethyl

## Abstract

Aryl trifluoromethyl diazomethanes **2‐R** (R=Ph, OMe, CF_3_) are readily decomposed by the (*o*‐carboranyl)diphosphine gold(I) complex **1**. The resulting α‐CF_3_ substituted carbene complexes **3‐R** have been characterized by multi‐nuclear NMR spectroscopy as well as X‐ray crystallography (for **3‐Ph**). The bonding situation was thoroughly assessed by computational means, showing stabilization of the electrophilic carbene center by π‐donation from the aryl substituent and backdonation from Au, as enhanced by the chelating P^P ligand. Reactivity studies under stoichiometric and catalytic conditions substantiate typical carbene‐type behavior for **3‐Ph**.

## Graphical abstract

Using an (*o*‐carboranyl)diphosphine ligand, α‐CF_3_ gold(I) carbenes were isolated and fully characterized. Their stabilization involves Au‐to‐C_carb_ backdonation and arene‐to‐C_carb_ π‐donation. They readily engage in carbene‐type transformations under both stoichiometric and catalytic conditions.

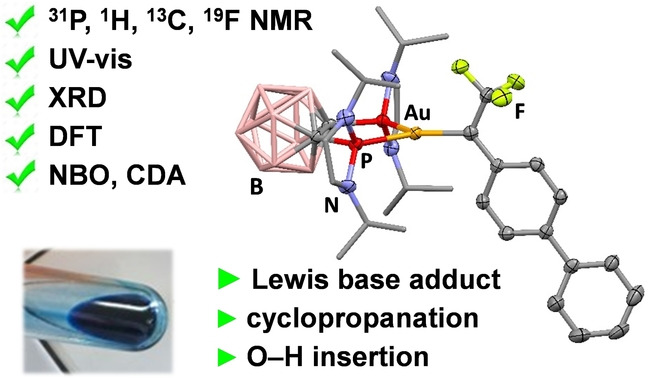

## Introduction

**Figure 1:**
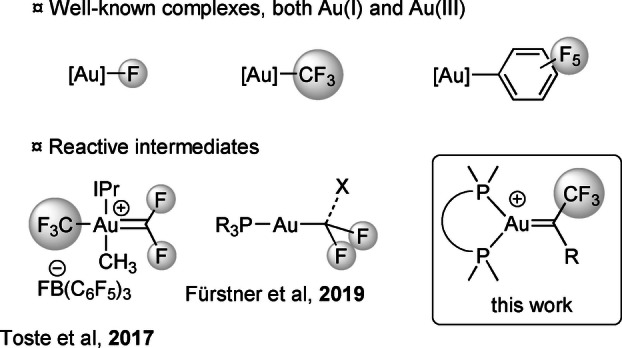
Different classes of F‐containing gold complexes.

The interplay of gold and fluorine, two peculiar elements within the periodic table, has turned out to a very active field of research in recent years (Figure 1).^[1]^ Accordingly, fluoro, trifluoromethyl and pentafluorophenyl Au^I^ and Au^III^ complexes have become well‐known and powerful species from both mechanistic and synthetic viewpoints.[^2^, ^3^, ^4^, ^5^, ^6^, ^7^] Highly reactive gold complexes featuring organofluoro moieties are also starting to be investigated. Toste et al. proposed in 2017 a F‐rebound mechanism involving Au^III^ difluorocarbenes as key intermediates to account for C(sp^3^)−CF_3_ coupling at gold.^[8]^ In 2019, Fürstner et al. spectroscopically characterized at low temperature a series of gold(I) difluorocarbenoids supported by phosphine ligands.^[9]^ Our interest in highly reactive gold complexes and the ability of *o*‐carboranyl diphosphine ligands to stabilize gold(I) carbene species thanks to enhanced π‐backdonation^[10]^ prompted us to investigate α‐CF_3_ complexes (Figure 1). A comprehensive study combining synthesis, NMR and crystallographic characterization, theoretical analysis of the bonding situation and reactivity studies is reported hereafter.

α‐CF_3_ carbene complexes are important and powerful intermediates in transition metal‐catalyzed transformations enabling the rapid construction of CF_3_‐containing derivatives. They are typically generated via diazo decomposition and engaged in cycloaddition or insertion reactions. Ru and Cu are the most used and studied metals in this context,^[11]^ but recently, major achievements have been reported with Fe,^[12]^ Ag^[13]^ and Pd^[14]^ catalysts as well. Strikingly, despite the great interest and potential of gold carbene complexes,[^15^, ^16^] we found only two isolated examples involving an α‐CF_3_ species, namely the C−H functionalization of phenothiazine/carbazole with PhC(=N_2_)CF_3_ catalyzed by (phosphite)AuCl/AgSbF_6_.^[17]^

**Figure 2:**
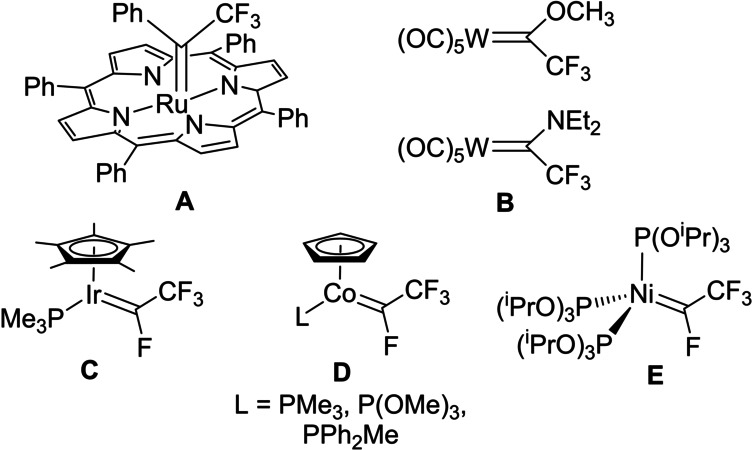
Known α‐CF_3_ carbene complexes.

In parallel to the synthetic developments, efforts have been made to prepare and characterize α‐CF_3_ carbene complexes, but only very few such species have been reported so far (Figure 2): the porphyrin Ru complex **A**,^[18]^ the Fischer‐type W complexes **B** ^[19]^ and the Schrock‐type Ir, Co and Ni fluoro complexes **C**–**E**.^[20]^ Of note, the Co and Ni carbene complexes **D** and **E** display reactivity relevant to the metathesis/polymerization of fluoro alkenes, but none of the isolated α‐CF_3_ carbene complexes was shown to undergo cyclopropanation or insertion reactions.

## Results and Discussion

### Synthesis, Spectroscopic and Structural Characterization

**Scheme 1:**
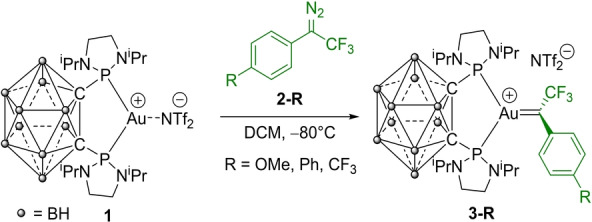
Formation of the gold(I) trifluoromethyl carbenes **3** by diazo decomposition.

To generate the targeted carbene complexes, diazo decomposition was chosen as a mild and versatile route, with only N_2_ as byproduct.^[21]^ One equivalent of biphenyl trifluoromethyl diazomethane **2‐Ph** was added at −80 °C to the tricoordinate pseudo‐cationic complex (P^P)AuNTf_2_ **1** ^[22]^ in dichloromethane (DCM) (Scheme 1). The diphosphine *o*‐carboranyl (DPCb) ligand was used for its unique chelation property, resulting in a bent L_2_Au^+^ fragment with higher π‐backdonation capacity.^[15d]^ With monodentate phosphines such as JohnPhos, no sign of a Au^I^ trifluoromethyl carbene could be observed by NMR spectroscopy even at low temperature.

**Figure 3:**
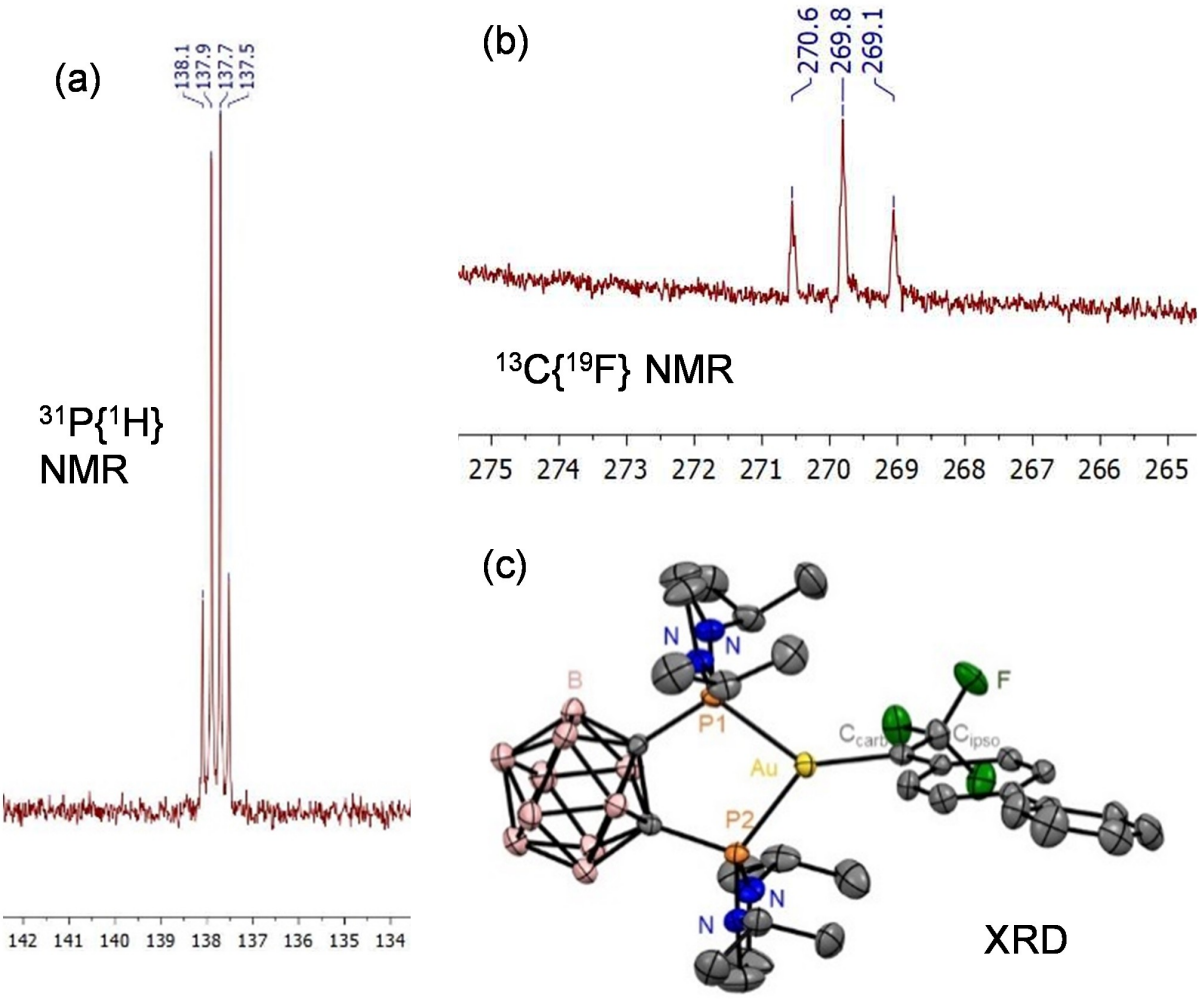
Diagnostic ^31^P{^1^H} and ^13^C{^19^F} NMR signals for **3‐Ph** (243 K, CD_2_Cl_2_, 121 and 126 MHz, respectively); molecular structure of **3‐Ph**. Thermal ellipsoids drawn at 50 % probability, hydrogen atoms, counter anion and disordered atoms are omitted. Selected bond lengths [Å] and bond angles [°]: Au−C_carb_ 1.971(2), Au−P1 2.347(1), Au−P2 2.348(1), C_carb_−C_ipso_ 1.444(6), C_carb_−CF_3_ 1.500(6); P1‐Au‐P2 90.59(4), P1‐Au‐C_carb_ 135.7(1), P2‐Au‐C_Carb_ 133.7(1).

Small bubbles of dinitrogen immediately evolved and the solution turned deep blue. After quick work up at −40 °C, the formed species was spectroscopically characterized. The ^31^P NMR signal (Figure 3a) appears at very similar chemical shift as the gold precursor (*δ* 137.8 and 138.2 ppm, respectively), but as a quartet instead of a singlet. The quartet multiplicity results from PF coupling, as unambiguously established by the presence of a triplet signal in the ^19^F NMR spectrum with similar coupling (23.2 Hz). These patterns suggest the formation of the desired (P^P)Au=C(CF_3_)(biphenyl)^+^ complex **3‐Ph**, something that was definitely confirmed by ^13^C NMR spectroscopy. The ^19^F‐decoupled spectrum (Figure 3b) shows a diagnostic signal at *δ* 269.8 ppm. In line with the blue color, a strong absorption is found at 623 nm in UV/Vis spectroscopy. Carbene **3‐Ph** proved to be moderately stable at room temperature, about 30 % decomposition being observed after 24 hours in DCM.

The *para*‐substituent at the phenyl ring was then changed for OMe and CF_3_ to assess the impact of electron‐donating/withdrawing groups. In both cases, the corresponding carbene complexes **3‐OMe** and **3‐CF_3_** were formed (Scheme 1) and NMR data in line with those of **3‐Ph** were obtained.^[23]^ Some features deserve comment: *i*) the diagnostic ^13^C NMR signal barely shifts (*δ* 268.5 ppm for **3‐OMe**, 265.8 ppm for **3‐CF_3_**); *ii*) the ^2^ *J*_PC_ and ^4^ *J*_PF_ coupling constants decrease in the series *p*‐CF_3_>*p*‐Ph>*p*‐OMe (from 109.0 to 79.5 Hz for ^2^ *J*_PC_, and from 32.1 to 14.0 Hz for ^4^ *J*_PF_), the carbene becomes less electron‐deficient and the Au=C bond order/double bond character decreases (see the DFT optimizations and bonding analyses below); *iii*) the carbene **3‐CF_3_** displays a long‐range ^8^ *J*_PF_ coupling of 10.8 Hz, as apparent in the ^31^P and ^19^F NMR spectra.^[24]^ It is also worth noting that if the carbene complex **3‐OMe** survives for a few hours at room temperature, as **3‐Ph**, **3‐CF_3_** is much less stable. All attempts to work it up at room or low temperature resulted in complete degradation. It was thus characterized *in situ* at low temperature.

Efforts were then made to obtain crystallographic data to know more about the structure of the obtained carbene. Gratifyingly, crystals suitable for X‐ray diffraction analysis were obtained by slow diffusion of pentane into a dichloromethane solution of **3‐Ph** at −40 °C (Figure 3c).[^23^, ^25^] The assymetric unit contains two molecules of very similar geometries (Table S2).^[23]^ For sake of simplicity, the key features of only one will be discussed here. Accordingly, the carbene complex adopts an ion pair structure. The DPCb ligand symmetrically chelates gold with Au−P bond lengths of 2.347(1)/2.348(1) Å and a P−Au−P bite angle of 90.59(4)°. The carbene center is in a perfectly planar environment (as apparent from the sum of bond angles of 360.0°) and it is oriented perpendicularly to the P−Au−P coordination plane (the mean planes around Au and C_carb_ make an angle of 89.76°). This orientation minimizes steric repulsions between the carbene and phosphorus substituents, it also maximizes the d(Au) to 2p^π^(C_carb_) backdonation (see below). The Au=C bond length (1.971(2) Å) is in the lower range of those reported for gold(I) carbene complexes.^[26]^ The carbene center is also stabilized by π‐donation from the biphenyl substituent, as indicated by their coplanar arrangement (the mean plane of the phenyl ring bonded to C_carb_ is rotated by only 2.5° from the carbene coordination plane) and from the relatively short C_carb_−C_ipso_ bond length (1.444(6) Å).

### Structure and Bonding Analysis

To gain more insight into the bonding situation and stabilization mode of the α‐CF_3_ gold(I) carbenes **3**,^[27]^ DFT (Density Functional Theory) calculations (B3PW91/SDD+f(Au),6‐31G** (other atoms)) were performed.^[23]^ The geometry optimized for **3‐Ph** (Table 1) matches well that determined crystallographically with deviations of less than 0.08 Å and 2.2° in the key bond lengths and angles (Table S2).^[23]^ The carbenes **3‐OMe** and **3‐CF_3_** were also computed, showing similar structures as **3‐Ph**. The small variations found in the Au=C/C_carb_−C_ipso_ bond lengths and associated Wiberg bond indexes (WBI) (Table 1) are in line with the electron bias induced by the *para* substituent, i.e. stronger arene‐to‐C_carb_ π‐donation and weaker Au‐to‐C_carb_ backdonation from **3‐CF_3_** to **3‐Ph**, and **3‐OMe** (see below). The NMR data for **3‐CF_3_**, **3‐Ph** and **3‐OMe** were also calculated. The trends observed experimentally for the ^2^ *J*_PC_ and ^4^ *J*_PF_ couplings were nicely reproduced (Table S3).^[23]^

**Table 1:** Data computed for the Au^I^ α‐CF_3_ carbene complexes **3‐Ph**, **3‐OMe**, **3‐CF_3_** at the B3PW91/SDD+f(Au),6‐31G**(other atoms) level of theory: selected bond lengths/angles, Wiberg bond indexes (WBI), charge transfer (CT) from the carbene to the (P^P)Au^+^ fragment, NLMO associated with the aryl‐to‐C_carb_ π‐donation and Au‐to‐C_carb_ backdonation (contribution of C_carb_), donation/backdonation (d/b) ratio as estimated by Charge Decomposition Analysis (CDA).

	**3‐OMe**	**3‐Ph**	**3‐CF_3_**
Geometric parameters			
Au=C_carb_ [Å]	1.987	1.983	1.971
C_carb_−C_ipso_ [Å]	1.423	1.429	1.442
Au−C−C${}_{\mathrm{CF}{}_{3}}$ [°]	114.57	114.76	114.97
Au−C−C_Aryl_ [°]	128.56	128.42	128.31
C_Aryl_−C−C${}_{\mathrm{CF}{}_{3}}$ [°]	116.85	116.82	116.71
PAuP[°]	89.81	89.88	89.79
	
NBO Analysis			
WBI (Au=C_carb_)	0.677	0.687	0.710
WBI (C_carb_−C_ipso_)	1.312	1.289	1.234
CT (e)	−0.10	−0.14	−0.25
d_*xz*_(Au)→C_carb_ backdonation %C_carb_ in NLMO d_*xz*_(Au)	7.3 %	9.7 %	15.5 %
π_C=Caryl_→C_carb_ donation %C_carb_ in NLMO π_C=Caryl_	24.2 %	16.3 %	10.1 %
	
CDA Analysis			
d/b ratio	2.16	2.09	1.86

The bonding situation was then assessed in detail via Natural Bond Orbital (NBO) and Charge Decomposition Analyses (CDA) (Table 1). The C_carb_‐to‐Au charge transfer (CT) is slightly negative for the three carbenes, a little more for the electron‐deprived carbene **3‐CF_3_** (−0.25 e) than for the electron‐enriched one **3‐OMe** (−0.10 e). CT values close to zero suggest that overall the C_carb_‐to‐Au donation and Au‐to‐C_carb_ backdonation roughly compensate each other. The donation/backdonation ratio (d/b), as estimated by CDA, falls in the 1.8–2.2 range (slightly lower for the electron‐deprived carbene **3‐CF_3_**, slightly higher for the electron‐enriched carbene **3‐OMe**), indicating C_carb_‐to‐Au donation prevails, but Au‐to‐C_carb_ backdonation is significant.

**Figure 4:**
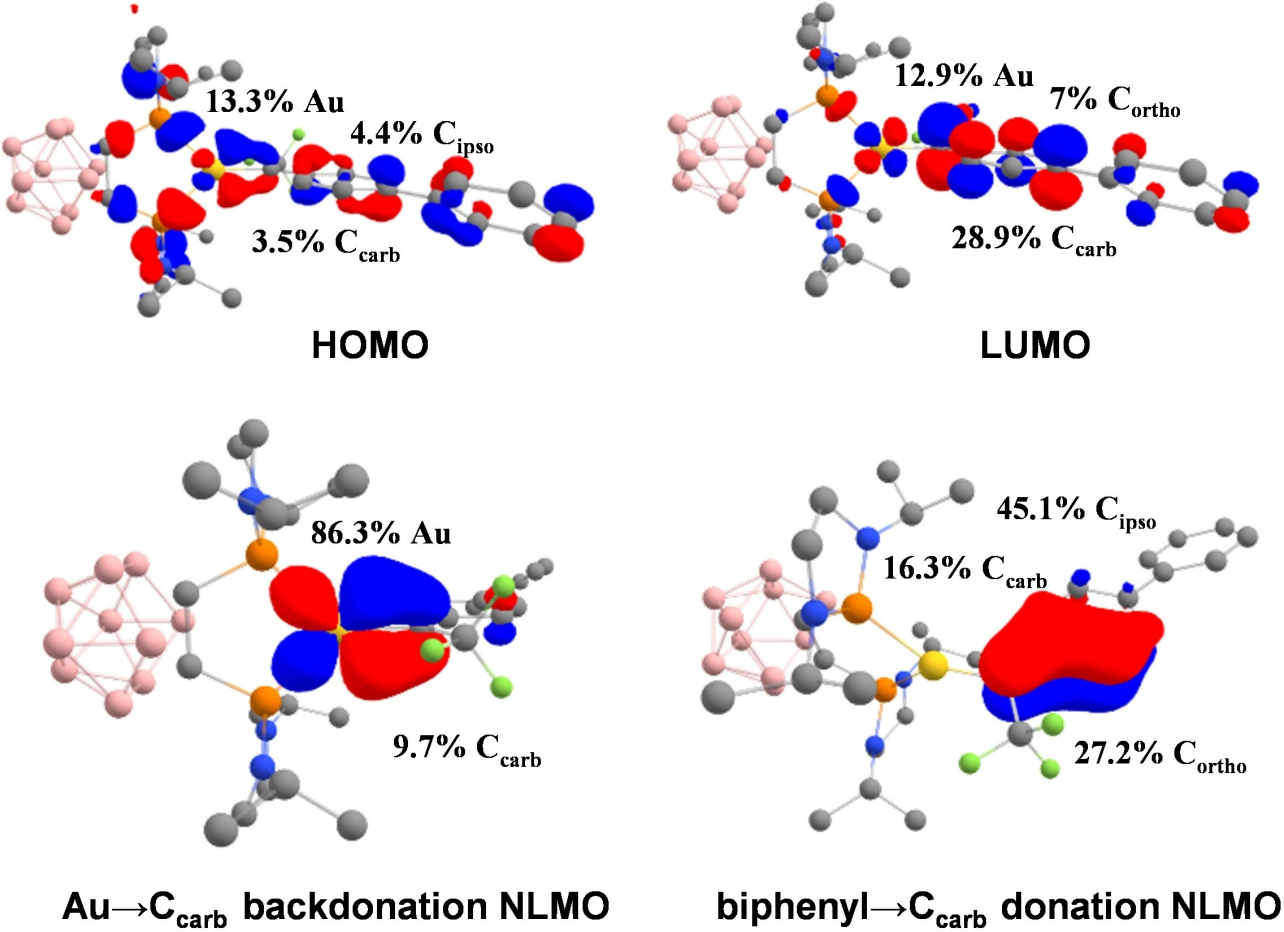
HOMO, LUMO and NLMO (cutoff: 0.04) associated with the arene‐to‐C_carb_ π‐donation and Au‐to‐C_carb_ backdonation computed for the Au^I^ α‐CF_3_ carbene complex **3‐Ph** at the B3PW91/SDD+f(Au),6‐31G**(other atoms) level of theory. Contribution of the main atoms (in %) in the frontier orbitals and NLMO.

Inspection of the molecular orbitals provides useful insight. The HOMO is centered on gold and is associated with an in‐plane d(Au) orbital in bonding combination with the 2p^π^(C_carb_) orbital. Reciprocally, the LUMO is centered on the carbene center and corresponds to the 2p^π^(C_carb_) vacant orbital in anti‐bonding combination with the d(Au) orbital (Figure 4, top). In addition, the π‐system of the aryl substituent is involved in these frontier orbitals, in line with π‐donation from the biphenyl substituent to the carbene center. This description is confirmed by referring to the Natural Localized Molecular Orbitals (NLMO). The Au‐to‐C_carb_ backdonation and aryl‐to‐C_carb_ π‐donation are apparent from the contributions of 2p^π^(C_carb_) in the d(Au)‐centered and π‐aryl orbitals, 9.7 and 16.3 %, respectively (Figure 4, bottom). Both interactions stabilize the carbene by filling partially its vacant orbital. The strength of the two interactions mildly evolves in the **3‐CF_3_**, **3‐Ph**, **3‐OMe** series. As expected from the electron‐withdrawing/releasing effect of the *para* substituent, the C_carb_ contribution is the largest in the d(Au) NLMO for **3‐CF_3_**, while for **3‐OMe**, it is in the π(arene) NLMO (Table 1 and Figure S37). Of note, the energy gap between the HOMO/LUMO frontier orbitals is relatively small (2.34 eV from DFT, 1.95 eV from TD‐DFT (Time‐Dependent Density Functional Theory) for **3‐Ph**, Figure S38).^[23]^ Consistently, TD‐DFT calculations taking into account solvent effects (DCM) by SMD (solvation model based on density) predict a low‐energy symmetry‐allowed electronic transition at 635 nm (HOMO→LUMO) that nicely matches the absorption band found experimentally at 623 nm (Figure S39 and Table S5).^[23]^

**Figure 5:**
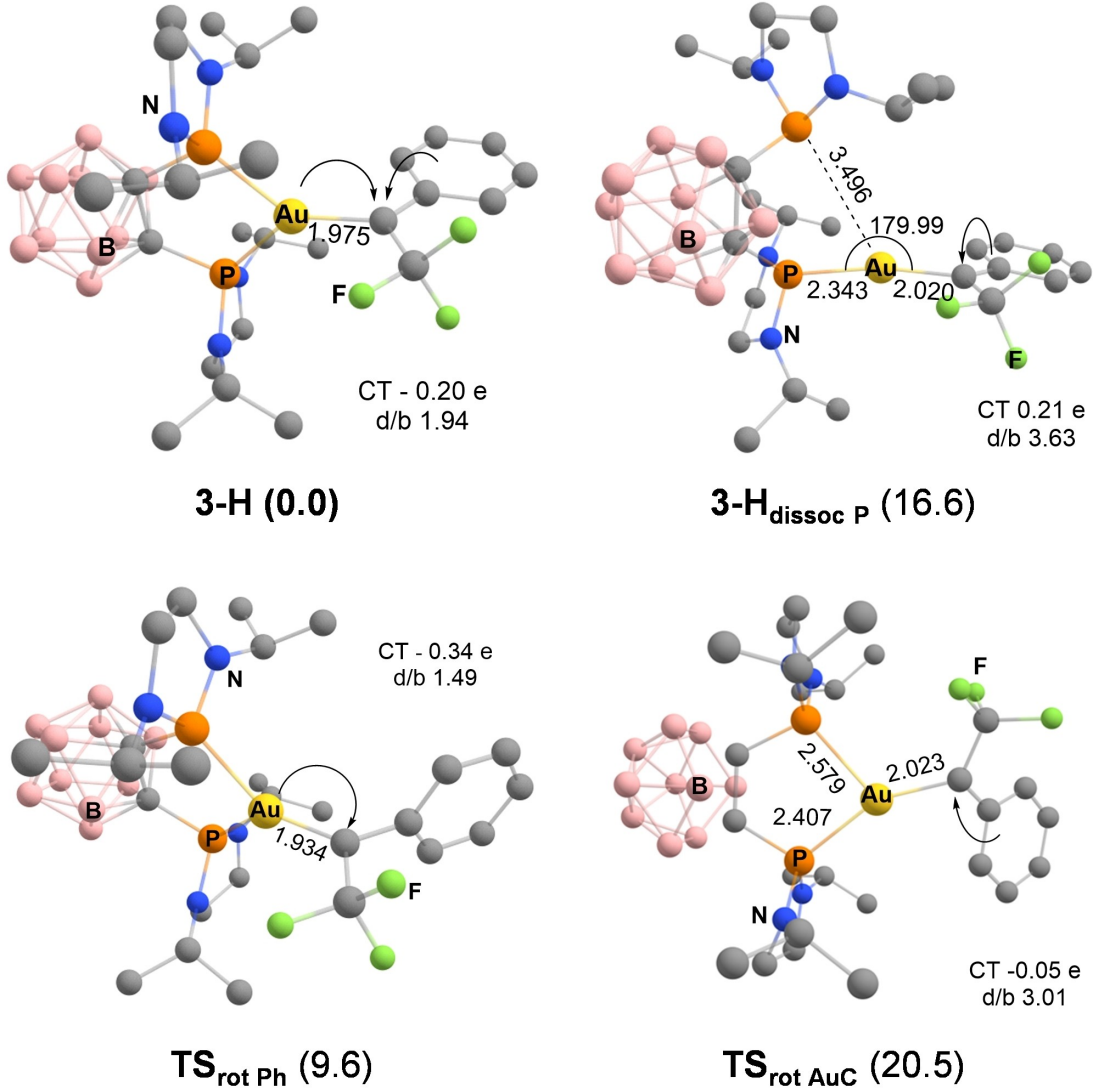
Optimized geometry of the parent α‐CF_3_ Au^I^ carbene **3‐H**, the form resulting from dissociation of one P atom (**3‐H_dissoc P_**) and the transition states associated with rotations about the C_carb_−C_ipso_ (**TS_rot Ph_**) and AuC (**TS_rot AuC_**) bonds at the B3PW91/SDD+f(Au),6‐31G**(other atoms) level of theory. Charge transfer (CT) and donation‐to‐backdonation (d/b) ratio. In parenthesis, related Gibbs free energy, in kcal mol^−1^.

To assess further the stabilization effect and relative importance of the arene‐to‐C_carb_ π‐donation and Au‐to‐C_carb_ backdonation,^[28]^ the “dynamics” of the parent α‐CF_3_ gold(I) carbene **3‐H** were analyzed. A first transition state was located on the potential energy surface (PES) for the rotation about the Au=C_carb_ bond (Figure S41 and Table S7) with the two phosphine arms coordinated to gold (**TS_rot AuC_**).[^29^, ^30^] It lies 20.5 kcal mol^−1^ above **3‐H** (Figure 5). From **3‐H** to **TS_rot AuC_**, we notice an elongation of the metal–carbene bond from 1.975 to 2.023 Å and a shortening of the C_carb_−C_ipso(Ph)_ bond from 1.438 to 1.427 Å (Tables S6 and S7). CDA and NBO Analyses of the bonding situation in **TS_rot AuC_** substantiate a decrease of the Au‐to‐C_carb_ backdonation, as deduced from the CT (−0.05 e vs −0.20 e in **3‐H**), the d/b ratio (3.01 vs 1.94 in **3‐H**) and the contribution of the C_carbene_ in the d(Au) NLMO (7.5 vs 14.8 % in **3‐H**). In addition, arene‐to‐C_carb_ π‐donation increases, as visible from the contribution of the C_carbene_ in the π_C=C(phenyl)_ NLMO (17.5 % in **TS_rot AuC_** vs 10.6 % in **3‐H**).

Rotation about the C_carb_−C_ipso_ bond is also possible and actually turned out to be easier. Forcing the phenyl group to be perpendicular to the carbene center costs ca. 10 kcal mol^−1^ only (**TS_rot Ph_**). It induces some shortening of the Au=C_carb_ bond length (from 1.975 to 1.934 Å). In the absence of π‐donation from the phenyl substituent, Au‐to‐C_carb_ backdonation is further enhanced in **TS_rot Ph_**, as apparent from the more negative C_carb_‐to‐Au CT (−0.34 e vs −0.20 e in **3‐H**), the higher 2p^π^(C_carb_) contribution in the d(Au) NLMO (17.6 vs 14.8 % in **3‐H**, Table S7) and the lower d/b ratio (1.49 vs 1.95 in **3‐H**).

Forcing one of the P atom to dissociate from gold is more energetically demanding, the corresponding structure (**3‐H_dissoc P_**) being located 16.6 kcal mol^−1^ above **3‐H**. This results in T‐shape instead of trigonal geometry. The Au center is now about linear (with P−Au distances of 2.343/3.496 Å and a P−Au−C_carb_ bond angle of 179.99°) while the Au=C_carb_ bond length increases to 2.020 Å. In the absence of P^P‐chelation, Au‐to‐C_carb_ backdonation is decreased and the carbene center is mainly stabilized by π‐donation from the phenyl substituent. Accordingly, the C_carb_‐to‐Au CT turns positive (0.19 e), the 2p^π^(C_carb_) participation to the d(Au) NLMO decreases to 4.4 % and the d/b ratio increases to 3.60. The stability and bonding analysis of **3‐H**, **3‐H_dissoc P_**, **TS_rot AuC_** and **TS_rot Ph_** emphasize that arene‐to‐C_carb_ π‐donation and Au‐to‐C_carb_ backdonation act in concert to stabilize the carbene center, with key contribution of P^P‐chelation.

Of note, the absence of free rotation about the Au=C_carb_ bond at the NMR timescale is apparent from the inequivalency of the CH, CH_2_ and CH_3_ groups of the diazaphospholane moiety in the ^13^C NMR spectra. Conversely, the presence of a single set of signals for the *ortho* and *meta* CH groups of the phenyl substituent in the ^1^H and ^13^C NMR spectra is consistent with free rotation about the C_carb_−C_ipso_ bond.

Then the reactivity of the α‐CF_3_ gold(I) carbenes was investigated.

### Electrophilic Behavior

**Scheme 2:**
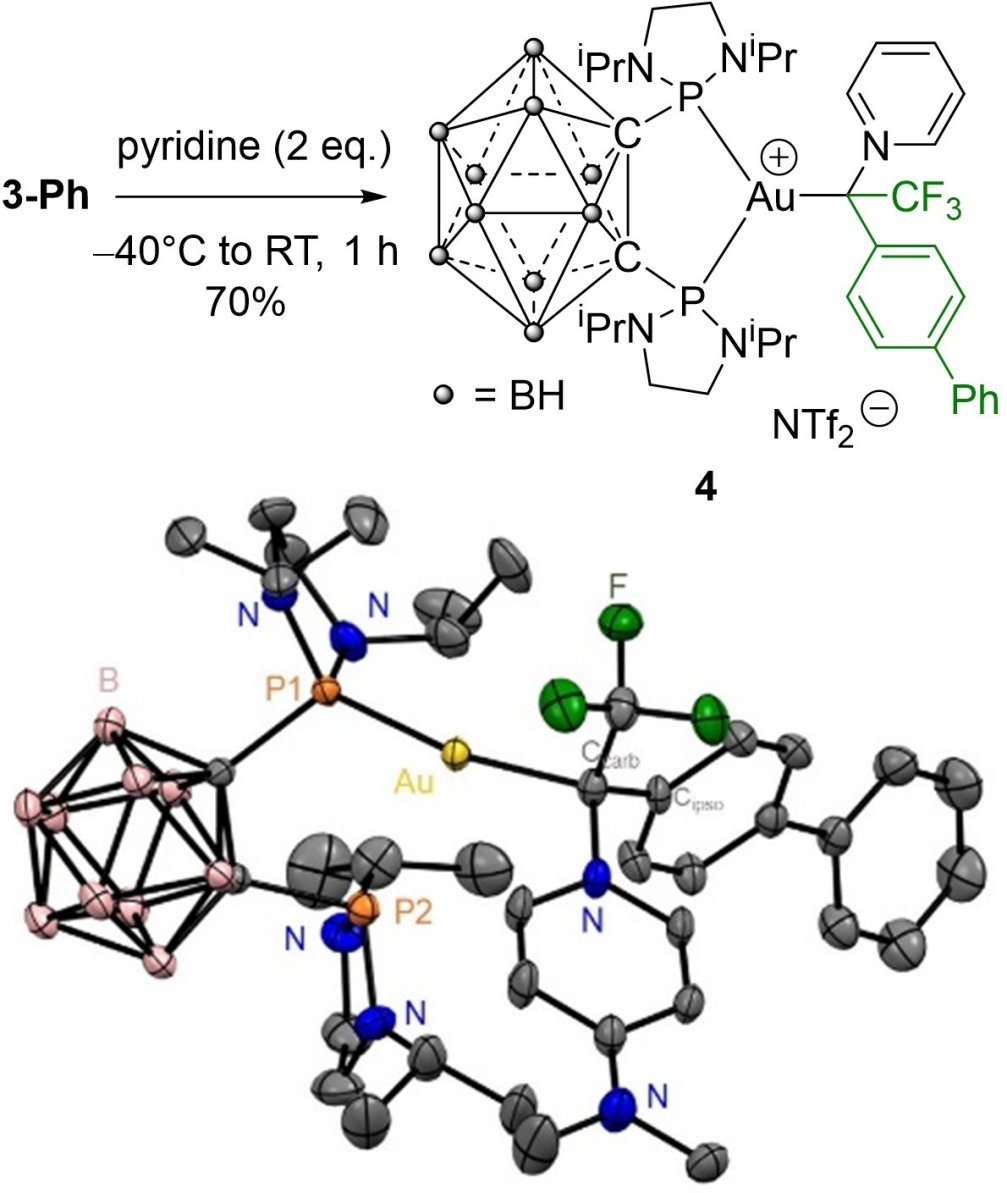
Reaction of the α‐CF_3_ Au^I^ carbene **3‐Ph** with pyridine; molecular structure of the related DMAP adduct **4′** (bottom). Thermal ellipsoids drawn at 50 % probability, hydrogen atoms, counter anion and disordered atoms are omitted. Selected bond lengths [Å] and bond angles [°]: Au−C_carb_ 2.108(4), Au−P1 2.254(1), Au−P2 3.606(1), C_carb_−C_ipso_ 1.530(5), C_carb_−CF_3_ 1.521(5), C_carb_−N 1.499(5); P1‐Au‐P2 68.14(3), P1‐Au‐C_carb_ 167.8(1), P2‐Au‐C_Carb_ 116.9(1).

Upon addition of pyridine (2 equiv) at −40 °C, the blue color characteristic of **3‐Ph** rapidly vanished, indicating rapid reaction. After work‐up, the carbene‐pyridine adduct **4** was isolated in 70 % yield as a pale‐yellow solid (Scheme 2). The NMR data are diagnostic for the addition of pyridine to the carbene center, not to gold. The ^13^C NMR signal at 269.8 ppm for the carbene center is shifted by more than 170 ppm and now appears at *δ* 98.4 ppm (quartet, ^2^ *J*_CF_=38.0 Hz). The ^1^H NMR signals for the H_ortho_ atoms of pyridine are significantly deshielded at *δ* 8.98 ppm, while the ^4^ *J*_PF_ coupling constant decreases from 23.2 to 5.8 Hz upon coordination of the Lewis base. Since all our attempts to crystallographically characterize **4** failed, we prepared the analogous 4‐dimethylamino‐pyridine (DMAP) adduct **4′**. Gratifyingly, crystals suitable for X‐ray diffraction analysis could be obtained in this case,^[25]^ unambiguously confirming the addition of the N‐Lewis base to the carbene center (Scheme 2, bottom). The pyridine N atom of DMAP is tightly coordinated to the former carbene center (N−C 1.499(5) Å) which is now in tetrahedral environment. Of note, only one of the P atom is coordinated to gold which adopts a quasi‐linear dicoordinate geometry (P−Au−C 167.81(11)°). The coordination of the Lewis base reduces the electrophilicity of the carbene center and gold atom. Combined with higher steric shielding, this prevents the two P atoms of the DPCb ligand from chelating to gold. It is likely that the gold fragment swings in between the two phosphorus atoms, as previously observed in the (DPCb)AuCl complexes.^[31]^ The reactions of **3‐Ph** with pyridine and DMAP substantiate its C‐centered electrophilic behavior, in line with a Fischer‐type carbene complex.

### Cyclopropanation Reactions

**Scheme 3:**
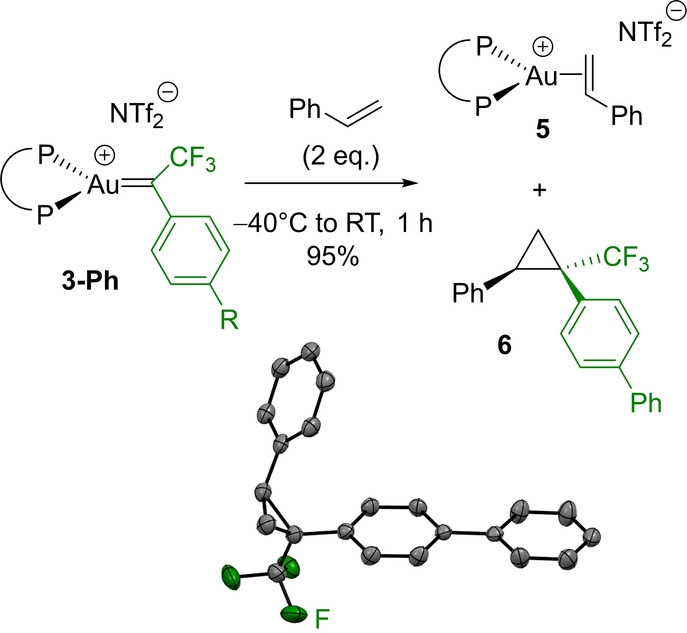
Reaction of the α‐CF_3_ Au^I^ carbene **3‐Ph** with styrene; molecular structure of the ensuing cyclopropane **6** (bottom) showing the *syn* relationship of the phenyl and biphenyl moieties (thermal ellipsoids drawn at 50 % probability, hydrogen atoms are omitted).

The α‐CF_3_ gold(I) carbene **3‐Ph** also readily reacts with styrene (Scheme 3).^[32]^ Two equivalents of alkene are needed for the reaction to reach completion (>95 % spectroscopic yield of cyclopropane **6**). The π‐alkene gold(I) complex **5** is obtained concomitantly, as deduced from ^31^P and ^1^H NMR spectroscopy.^[33]^

**Scheme 4:**
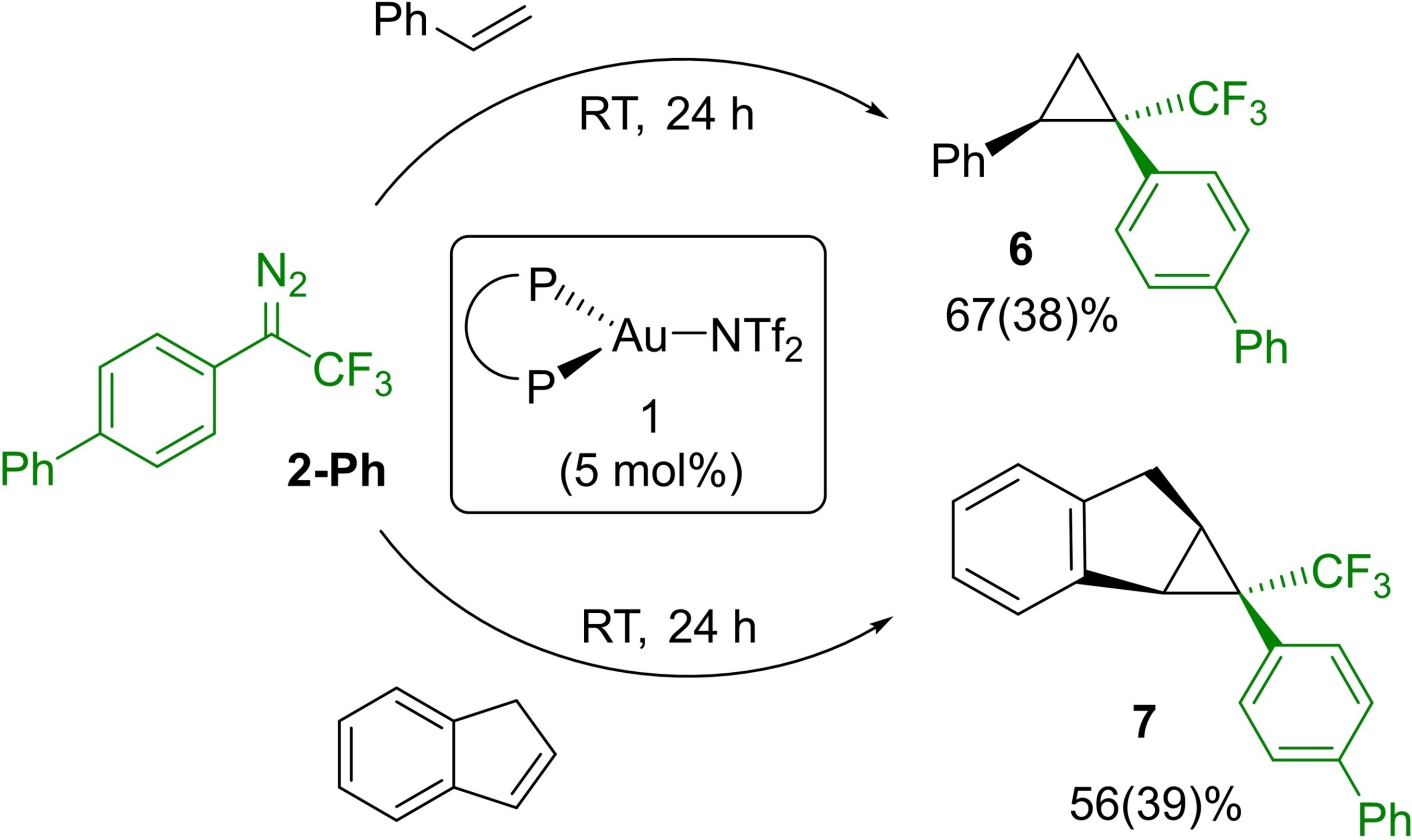
Gold‐catalyzed cyclopropanation reactions from the α‐CF_3_ diazo derivative **2‐Ph** (NMR yields determined by ^19^F NMR using 4,4′‐difluorobiphenyl as internal standard, isolated yields in parentheses).

The transformation is amenable to catalysis (Scheme 4). Starting from the diazo compound **2‐Ph**, the transformation can be performed under catalytic conditions. Using 5 mol % of the “cationic” complex (P^P)AuNTf_2_ **1** and 1.05 equivalent of styrene, **6** is obtained in 67 % yield after 24 hours at room temperature. Of note, the cyclopropanation is fully diastereoselective. The *syn* relationship of the phenyl and biphenyl groups was first deduced from {^1^H,^19^F} HOESY (Heteronuclear Overhauser Effect SpectroscopY) NMR experiments, and then definitely confirmed by X‐ray diffraction analysis.[^23^, ^25^] Under similar conditions, indene is smoothly converted into **7** (56 % spectroscopic yield), with again complete diastereoslectivity for the *syn* cyclopropane.

### Insertion Reactions

**Scheme 5:**
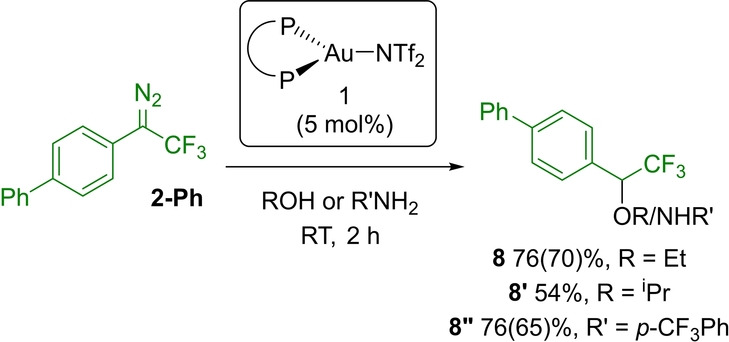
Gold‐catalyzed O−H/N−H insertion reactions from the α‐CF_3_ diazo derivative **2‐Ph** (NMR yields determined by ^19^F NMR using 4,4′‐difluorobiphenyl as internal standard, isolated yield in parentheses).

With alcohols, O−H insertion reactions also proceed readily under similar conditions (Scheme 5). The corresponding α‐CF_3_ ethers **8** and **8′** are obtained thereby in 76 and 54 % yields from ethanol and isopropanol, respectively.^[34]^ With anilines, N−H insertion occurs, as substantiated by the formation of the α‐CF_3_ amine **8′′**.[^11c^, ^11d^] Notably, attempts of C−H insertion from *N*‐heterocycles (N−H or N−Me indole/carbazole) led to complex mixtures.^[35]^

## Conclusion

Using an (*o*‐carboranyl)diphosphine ligand, we have been able to prepare and fully characterize α‐CF_3_ gold(I) carbenes. The bonding situation was thoroughly analyzed by experimental and computational means. Accordingly, the carbene is stabilized by a combination of Au‐to‐C_carb_ backdonation and arene‐to‐C_carb_ π‐donation, whose balance finely depends on the electronic properties of the aryl substituent (OMe, Ph, CF_3_).

Further studies will aim to explore and extend further the chemistry of α‐CF_3_ gold(I) carbene complexes. More generally, it is likely that other highly reactive (organo)fluoro gold complexes can be stabilized, isolated and exploited employing suitable ligands.

## Conflict of interest

The authors declare no conflict of interest.

1

## Supporting information

As a service to our authors and readers, this journal provides supporting information supplied by the authors. Such materials are peer reviewed and may be re‐organized for online delivery, but are not copy‐edited or typeset. Technical support issues arising from supporting information (other than missing files) should be addressed to the authors.

Supporting Information

Supporting Information

Supporting Information

Supporting Information

## Data Availability

The data that support the findings of this study are available in the Supporting Information of this article.
