# Supplementary material for: Gold(I) α‐Trifluoromethyl Carbenes: Synthesis, Characterization and Reactivity Studies
Source: Angew Chem Int Ed Engl. 2022 May 3;61(25):e202204781. doi: 10.1002/anie.202204781 (PMC9323441; doi:10.1002/anie.202204781)
Supplement: Supplementary file 1 — Supporting Information [file ANIE-61-0-s004.pdf]

Angewandte Chemie
Eine Zeitschrift der Gesellschaft Deutscher Chemiker

Supporting Information

Gold(I) α-Trifluoromethyl Carbenes: Synthesis, Characterization and Reactivity Studies

*M. Rigoulet, D. Vesseur, K. Miqueu, D. Bourissou**

Table of content

1. Materials and methods S3

2. Experimental procedures and analytical data S3

   2.1 Synthesis of the gold complex S3

   2.2 Synthesis of the diazo compounds **2-R** S3

   2.3 Synthesis of the α-CF_3 gold carbenes **3-R** S4

   2.4 Reactivity with Lewis bases S5

   2.5 Stoichiometric reaction with styrene S6

   2.6 Catalytic reactions with styrene, indene, ethanol, isopropanol & *p*-CF_3 aniline S6

3. NMR spectra S8

4. Crystallographic data S26

5. Computational details S27

6. Computational results S29

7. Z-matrices and energies in au S38

1. Materials and methods

Unless otherwise stated, all reactions and manipulations were carried out under an atmosphere of dry argon using standard Schlenk techniques or in a glovebox under an inert atmosphere. Dry, oxygen-free solvents were employed. Solution ^{1}H, ^{13}C, ^{31}P, ^{19}F and ^{11}B NMR spectra were recorded on Bruker Avance 300, 400 or 500 spectrometers at 298K unless otherwise stated. Chemical shifts are expressed with a positive sign, in parts per million, calibrated to residual ^{1}H and ^{13}C solvent signals. External 85% H_3PO_4, $CFCl_3$ and $BF_3.OEt_2$ were used as reference for ^{31}P, ^{19}F and ^{11}B NMR, respectively. The following abbreviations and their combinations are used: br, broad; s, singlet; d, doublet; t, triplet; q, quartet; quin, quintuplet; m, multiplet. The ^{1}H and ^{13}C resonance signals were attributed by means of 2D HSQC and HMBC. Mass spectra were recorded on a Waters UPLC Xevo G2 Q TOF apparatus. The UV-vis spectrum was recorded on an Agilent Cay 60 UV-Vis apparatus. Dilithio-1,2-dicarba-*closo*-dodecaborane,[1] 2-chloro-1,3-diisopropyl-1,3,2-diazaphospholidine,[2] 1,2-bis(diaminophosphino)-1,2-dicarba-*closo*dodecaborane,[3] hydrazone[4] were prepared according to reported procedures. All other starting materials were purchased from Aldrich and used as received unless otherwise stated.

2. Experimental procedures and analytical data

2.1 Synthesis of the gold complex

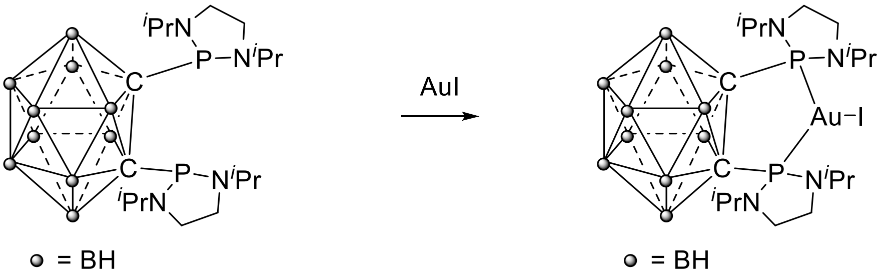

Prepared in a similar way than the AuCl complex,[3] with AuI instead of (DMS)AuCl.
^{31}P{^{1}H} NMR (121 MHz, $CDCl_3$): δ 141.0 (s).

2.2 Synthesis of the diazo compounds 2-R

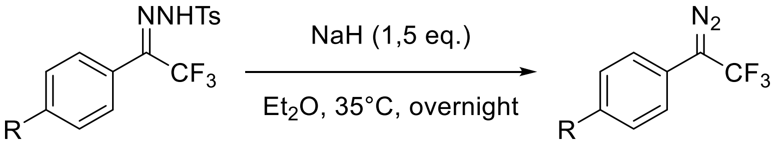

In a Schlenk flask, under argon, the hydrazone was dissolved in Et_2O (0.3 M). A solution of NaH in Et_2O (0.3 M) was added stepwise over the solution of hydrazone. The reaction mixture was stirred overnight at 35°C. The solution was filtered and the product was purified by silica gel chromatography (passivated with Et_3N, eluent: pentane). CAUTION: diazo compounds are presumed to be toxic and potentially explosive and should be handled with care, in a fume hood.
Data for 1-(1-diazo-2,2,2-trifluoroethyl)-4-methoxybenzene, 4-(1-diazo-2,2,2-trifluoroethyl)-1,1'-biphenyl and 1-(1-diazo-2,2,2-trifluoroethyl)-4-trifluoromethylbenzene are consistent with literature.[5]

[1] B. Wrackmeyer, E. V. Klimkina, W. Milius, *Appl. Organomet. Chem.* **2010**, *24*, 25–32.
[2] J. Krysiak, C. Lyon, A. Baceiredo, H. Gornitzka, M. Mikolajczyk, G. Bertrand, *Chem. – Eur. J.* **2004**, *10*, 1982–1986.
[3] M. Joost, L. Estévez, S. Mallet-Ladeira, K. Miqueu, A. Amgoune, D. Bourissou, *Angew. Chem. Int. Ed.* **2014**, *53*, 14512–14516.
[4] E. Emer, J. Twilton, M. Tredwell, S. Calderwood, T. L. Collier, B. Liégault, M. Taillefer, V. Gouverneur, *Org. Lett.* **2014**, *16*, 6004–6007.
[5] S. Hyde, J. Veliks, D. M. H. Ascough, R. Szpera, R. S. Paton, V. Gouverneur, *Tetrahedron*, **2019**, *75*, 17–25.

2.3 Synthesis of the α-CF_3 gold carbenes 3-R

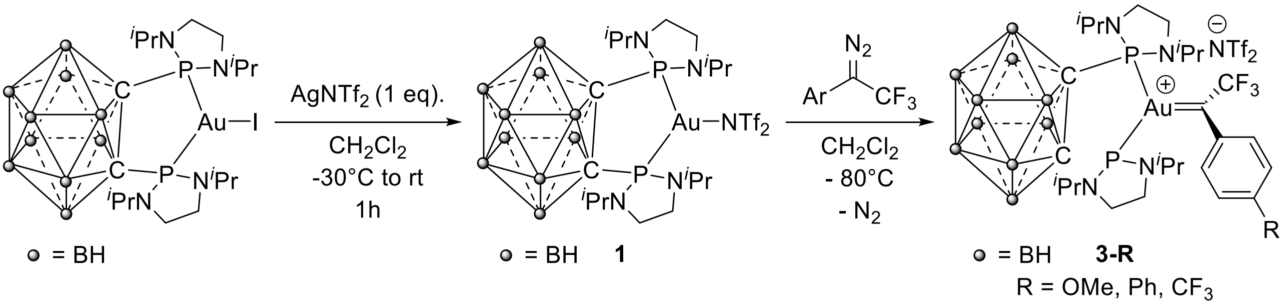

Preparation of complex 1: A solution of (DPCb)AuI (326 mg, 0.40 mmol) in dichloromethane (8 mL) was added to a dispersion of $AgNTf_2$ (155 mg, 0.40 mmol, 1.0 eq) in dichloromethane (4 mL) at –20 °C and stirred for 1 hour under protection from light while slowly warming up to room temperature. The reaction mixture was filtered over a pad of celite to give a clear yellow filtrate.
$^{31}P\{^1H\}$ NMR (121 MHz, CD_2Cl_3): δ 138.2 (s)

A solution of the diazo compound **2-R** (0.40 mmol, 1.0 eq) in dichloromethane (2 mL) was added to the solution of complex **1** at -80°C. At -40°C, the solvent was partially evaporated under vacuum. The product was precipitated by addition of pentane. The solvent was removed by filtration with a cannula. The solid residue was dissolved in toluene (partially soluble) and precipitated by addition of pentane. The solvent was removed by filtration with a cannula and the residue was dried under vacuum to give the desired α-CF_3 gold(I) carbene complex **3-R**.

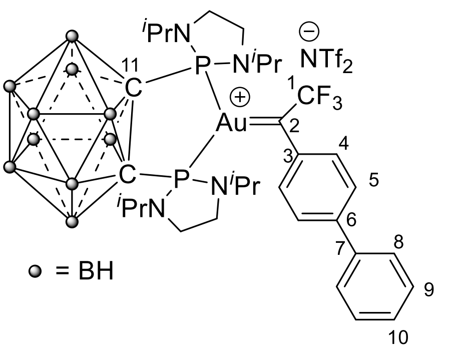

3-Ph: The pure product was obtained as a dark blue powder (428 mg, 89%). **$^{31}P\{^1H\}$ NMR** (121 MHz, $CDCl_3$, 298 K): δ 137.8 (q, $^4J_{PF}$ = 23.2 Hz). **^1H NMR** (500 MHz, CD_2Cl_2, 243 K): δ 8.00-7.88 (m, 2H, H_5), 7.88-7.82 (m, 2H, H_4), 7.81-7.78 (d, J_{HH} = 7.1 Hz, 2H, H_8), 7.55-7.49 (m, 3H, H_9 & H_{10}), 3.72-3.60 (m, 4H, C*H*($CH_3)_2$), 3.39-3.23 (m, 8H, N(C*H*$_2$)$_2$N), 3.01-1.70 (bs, 10H, BH), 1.36 (d, 6H, J_{HH} = 6.5 Hz, CH(C*H*$_3$)$_2$), 1.34 (d, 6H, J_{HH} = 6.6 Hz, CH(C*H*$_3$)$_2$), 1.19 (d, J_{HH} = 6.5 Hz, 6H, CH(C*H*$_3$)$_2$), 1.04 (d, J_{HH} = 6.6 Hz, 6H, CH(C*H*$_3$)$_2$). **$^{13}C\{^1H\}$ NMR** (126 MHz, CD_2Cl_2, 243K): δ 269.8 (qt, $^2J_{CF}$ = 34.9 Hz, $^2J_{CP}$ = 94.4 Hz, C_2), 150.6 (t, J_{PC} = 12.8 Hz, C_6), 143.6 (m, C_3), 138.1 (t, J_{PC} = 6.1 Hz, C_7), 130.6 (s, C_{10}), 129.6 (s, C_8 & C_9), 128.3 (t, J_{PC} = 9.5 Hz, C_4), 127.7 (t, J_{PC} = 3.7 Hz, C_5), 119.8 (q, $^1J_{CF}$ = 321.1 Hz, NTf_2), 90.2 (t, $^1J_{PC}$ = 25.8 Hz, C_{11}), 50.5 (*pseudo* q, J_{CP} = 8.7 Hz, *C*H($CH_3)_2$), 42.6 (s, N(*C*H$_2$)$_2$N), 42.2 (s, N(*C*H$_2$)$_2$N), 21.6 (s, CH(*C*H$_3$)$_2$), 21.5 (s, CH(*C*H$_3$)$_2$), 21.2 (s, CH(*C*H$_3$)$_2$), 19.9 (s, CH(*C*H$_3$)$_2$). **^{19}F NMR** (282 MHz, $CDCl_3$): δ -54.8 (t, $^4J_{PF}$ = 23.2 Hz, CF_3), -79.7 (s, NTf_2). **$^{11}B\{^1H\}$ NMR** (96 Hz, CD_2Cl_2): δ -2.5, -10.1, -16.9. **HRMS (ESI$^+$):** calculated for $[M]^+$ = $C_{32}H_{55}B_{10}N_4P_2F_3Au^+$: 920.4510. Found: 920.4500.

Due to the coupling with F and P, in addition to overlap with some signals for aromatic carbon atoms, the signal for C_1 could not be assigned.

Crystals suitable for XRD analysis were obtained by slow diffusion of pentane into a concentrated solution of **3-Ph** in DCM at 4°C.

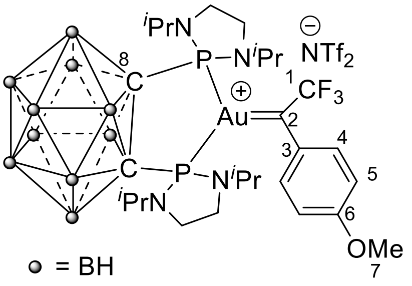

3-OMe: The pure product was obtained as a dark blue powder (240 mg, 52%). **^{31}P{^{1}H} NMR** (121 MHz, $CDCl_3$): δ 142.1 (q, $^{4}J_{PF}$ = 14.1 Hz). **^{1}H NMR** (500 MHz, CD_2Cl_2): δ 7.90 (dq, J_{HH} = 9.1 Hz, $^{5}J_{HF}$ = 5.0 Hz, 2H, H_4), 7.20 (d, J_{HH} = 9.1 Hz, 2H, H_5), 4.07 (t, J_{HH} = 1.4 Hz, H_7), 3.74-3.61 (m, 4H, C*H*$(CH_3)_2$), 3.36-3.33 (m, 8H, N(C*H*$_2$)$_2$N), 3.18-1.71 (bs, 10H, H_{BH}), 1.38-1.32 (m, 12H, CH(C*H*$_3$)$_2$), 1.19 (d, J_{HH} = 6.5 Hz, 6H, CH(C*H*$_3$)$_2$), 1.04 (d, J_{HH} = 6.5 Hz, 6H, CH(C*H*$_3$)$_2$). **^{13}C{^{1}H} NMR** (126 MHz, CD_2Cl_2): δ 268.5 (qt, $^{2}J_{CF}$ = 37.2 Hz, $^{2}J_{CP}$ = 79.5 Hz, C_2), 171.3 (t, J_{CP} = 9.9 Hz, C_4), 140.0 (qt, $^{3}J_{CP}$ = 13.3 Hz, J_{CF} = 5.6 Hz, C_3), 129.5 (qt, , J_{CP} = 8.3 Hz, J_{CF} = 278.1 Hz, C_1), 120.4 (q, $^{1}J_{CF}$ = 321.4 Hz, NTf_2), 117.3 (t, J_{CP} = 5.9 Hz, C_5), 115.1 (s,C_6), 92.5 (t, J_{CP} = 26.5 Hz, C_8), 58.0 (s, C_7), 51.0 (t, J_{CP} = 8.1 Hz, *C*H$(CH_3)_2$), 50.9 (t, J_{CP} = 8.1 Hz, *C*H$(CH_3)_2$), 43.1 (s, N(*C*H$_2$)$_2$N), 42.7 (s, N(*C*H$_2$)$_2$N), 21.9 (s, CH(*C*H$_3$)$_2$), 21.4 (s, CH(*C*H$_3$)$_2$), 20.2 (s, CH(*C*H$_3$)$_2$), 19.0 (s, CH(*C*H$_3$)$_2$). **^{19}F{^{1}H} NMR** (282 MHz, $CDCl_3$): δ -53.9 (t, $^{4}J_{PF}$ = 14.1 Hz, CF_3), -78.5 (s, NTf_2). **HRMS (ESI$^+$):** calculated for $[M]^+$ = $C_{27}H_{53}B_{10}N_4P_2OF_3Au^+$: 874.4300. Found: 874.4297.

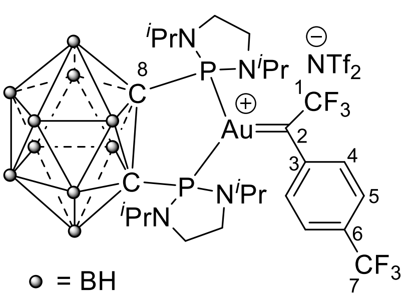

3-CF$_3$: work up led to decomposition. The complex was characterized directly after addition of the diazo derivative to **1**, that gave a deep purple solution. **^{31}P{^{1}H} NMR** (121 MHz, CD_2Cl_2, 298 K): δ 132.8 (qq, $^{4}J_{PF}$ = 32.1 Hz, $^{8}J_{PF}$ = 10.8 Hz). **^{1}H NMR** (400 MHz, CD_2Cl_2, 213 K): δ 7.80-7.73 (m, 4H, H_{Ar}), 3.70-3.54 (m, 4H, C*H*$(CH_3)_2$), 3.40-3.17 (m, 8H, N(C*H*$_2$)$_2$N), 2.96-1.65 (bs, 10H, H_{BH}), 1.31 (pseudo t, J_{HH} = 6.9 Hz, 12H, CH(C*H*$_3$)$_2$), 1.14 (d, J_{HH} = 6.5Hz, 6H, CH(C*H*$_3$)$_2$), 0.97 (d, J_{HH} = 6.4 Hz, 6H, CH(C*H*$_3$)$_2$). **^{13}C{^{1}H, ^{19}F} NMR** (100 MHz, CD_2Cl_2, 213 K): δ 265.8 (t, $^{2}J_{CP}$ = 109.0 Hz, C_2), 145.2 (t, $^{3}J_{CP}$ = 24.1 Hz, C_3), 134.7 (t, J_{CP} = 13.5 Hz, C_5), 132.4 (t, J_{CP} = 14.1 Hz, C_4), 129.1 (t, J_{CP} = 17.5 Hz, C_1), 126.3 (t, J_{CP} = 10.7 Hz, C_6), 123.0 (t, J_{CP} = 5.9 Hz, C_7), 119.3 (s, NTf_2), 87.7 (t, $^{1}J_{PC}$ = 25.9 Hz, C_8), 50.1 (t, J_{CP} = 6.8 Hz, *C*H$(CH_3)_2$), 50.0 (t, J_{CP} = 7.1 Hz, *C*H$(CH_3)_2$), 42.1 (s, N(*C*H$_2$)$_2$N), 41.7 (s, N(*C*H$_2$)$_2$N), 21.5 (s, CH(*C*H$_3$)$_2$), 21.0 (s, CH(*C*H$_3$)$_2$). 20.4 (s, CH(*C*H$_3$)$_2$), 19.6 (s, CH(*C*H$_3$)$_2$). **^{19}F{^{1}H} NMR** (282 MHz, CD_2Cl_2, 213 K): δ -54.5 (t, $^{4}J_{PF}$ = 32.1 Hz, CF_3), -64.2 (t, $^{8}J_{PF}$ = 10.8 Hz, CF_3), -79.4 (s, NTf_2). **^{11}B {^{1}H} NMR** (96 MHz, CD_2Cl_2, 298 K): δ -2.4, -6.8, -10.9, -16.9. **HRMS (ESI$^+$):** calculated for $[M]^+$ = $C_{27}H_{50}B_{10}N_4P_2F_6Au^+$: 912.4068. Found: 912.4077.

2.4 Reactivity with Lewis bases

To a solution of **3-Ph** (36 mg, 0.03 mmol) in dichloromethane (1.5 mL) was added a solution of pyridine (0.06 mmol) in DCM (0.5 mL) at -40°C. The reaction mixture was stirred for 1 h while slowly warming up to room temperature. Solvent was removed under vacuum and the solid was washed twice with pentane. After drying under vacuum, the carbene/pyridine adduct was obtained as a pale-yellow solid (27 mg, 0.021, 70%).

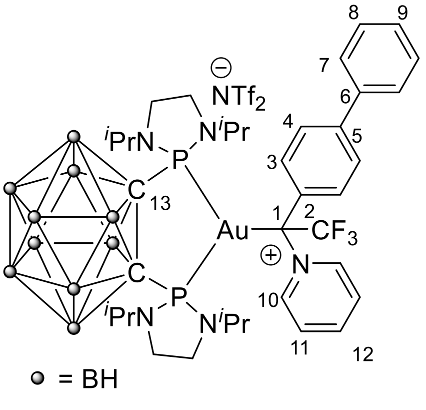

Compound 4: ^{31}P{^{1}H} NMR (202 MHz, $CDCl_3$): δ 127.6 (q, $^{4}J_{PF}$ = 5.8 Hz). **^{1}H NMR** (500 MHz, $CDCl_3$): δ 8.98 (d, J_{HH} = 6.2 Hz, 2H, H_{10}), 8.65 (t, J_{HH} = 7.7 Hz, 1H, H_{12}), 8.14 (t, J_{HH} = 7.1 Hz, 2H, H_{11}), 7.65 (d, J_{HH} = 8.5 Hz, 2H, H_4), 7.57 (d, J_{HH} = 7.5 Hz, 2H, H_7), 7.47 (t, J_{HH} = 7.7 Hz, 2H, H_8), 7.41-7.33 (m, 3H, H_3 & H_9), 3.69-3.57 (m, 4H, C*H*$(CH_3)_2$), 3.28-3.16 (m, 8H, N(C*H*$_2$)$_2$N), 2.96-1.65 (bs, 10H, H_{BH}), 1.22 (d, J_{HH} = 6.6 Hz, 6H, CH(C*H*$_3$)$_2$), 1.17 (d, J_{HH} = 6.6 Hz, 6H, CH(C*H*$_3$)$_2$), 1.08 (d, J_{HH} = 6.6 Hz, 6H, CH(C*H*$_3$)$_2$), 1.05 (d, J_{HH} = 6.5 Hz, 6H, CH(C*H*$_3$)$_2$). **^{13}C{^{1}H} NMR** (126 MHz, $CDCl_3$): δ 148.1 (s, C_{12}), 147.8 (s, C_{10}), 141.2 (s, C_5), 139.5 (s, C_6), 135.9 (s, C_2), 129.2 (s, C_8), 128.6 (s, C_{11}), 128.5 (s, C_4), 128.2 (s, C_9), 127.9 (s, C_3), 127.6 (q, $^{1}J_{CF}$ = 279.3 Hz, CF_3), 127.1 (s, C_7), 120.0 (q, $^{1}J_{CF}$ = 321.4 Hz, NTf_2), 98.4 (q, $^{2}J_{CF}$ = 38.0 Hz, J_{PC} not observed, C_1), 90.4 (t, J_{PC} = 32.2 Hz, C_{13}), 50.3 (t, J_{CP} = 12.5 Hz, *C*H$(CH_3)_2$), 50.0 (t, J_{CP} = 12.5 Hz, *C*H$(CH_3)_2$), 42.9 (s, N(*C*H$_2$)$_2$N), 42.8 (s, N(*C*H$_2$)$_2$N), 21.8 (t, J_{CP} = 3.1 Hz, CH(*C*H$_3$)$_2$), 21.7 (t, J_{CP} = 3.1 Hz, CH(*C*H$_3$)$_2$), 21.2 (s, CH(*C*H$_3$)$_2$), 20.7 (s, CH(*C*H$_3$)$_2$). **^{19}F NMR** (470 MHz, CD_2Cl_2, 213 K): δ -62.6 (bs, CF_3), -78.7 (s, NTf_2). **^{11}B {^{1}H} NMR** (160 MHz, $CDCl_3$): δ -1.3, -6.1, -10.0.

The related carbene/DMAP adduct **4'** was prepared analogously and crystals suitable for X-ray diffraction analysis were grown from a dichloromethane/pentane solution at -30°C.

2.5 Stoichiometric reaction with styrene

To a solution of **3-Ph** (28 mg, 0.023 mmol) in $CDCl_3$ (0.5 mL) was added a solution of styrene (5.3 µL, 0.046 mmol, 2 eq.) and 4,4'-difluorobiphenyl (2 mg, 0.011 mmol, 0.5 eq.) in $CDCl_3$ (0.1 mL) at -40°C. The reaction mixture was stirred manually and warmed up to room temperature. After 1 h, one diastereoisomer of the cyclopropane (with *cis* position of the biphenyl and phenyl groups) is formed quantitatively (>95%) according to $^{19}F\{^1H\}$ NMR. $^{31}P\{^1H\}$ NMR spectroscopy shows the formation of the π-complex **5** as side-product. The structure of **5** was confirmed by independent synthesis, following the procedure previously described for similar compounds.[6]

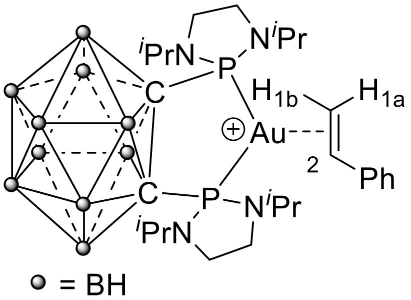

π-complex 5: $^{31}P\{^1H\}$ NMR (121 MHz, CD_2Cl_2): δ 127.5 (s). **^1H NMR** (300 MHz, $CDCl_3$): δ 7.42-7.33 (m, 2H, H_{Ar}), 7.28-7.20 (m, 1H, H_{Ar}), 7.18-7.12 (m, 2H, H_{Ar}), 5.70 (dd, J_{HH} = 13.9, 9.7 Hz, 1H, H_2), 4.27 (dd, J_{HH} = 13.9, 1.8 Hz, 1H, H_{1a}), 4.06 (dd, J_{HH} = 14.1, 1.8 Hz, 1H, H_{1b}), 3.63-3.31 (m, 4H, C*H*(CH_3)$_2$), 3.40-3.23 (m, 8H, N(C*H*$_2$)$_2$N), 2.96-1.65 (bs, 10H, H_{BH}), 1.37 (d, J_{HH} = 6.6 Hz, 6H, CH(C*H*$_3$)$_2$), 1.33 (d, J_{HH} = 6.6 Hz, 6H, CH(C*H*$_3$)$_2$), 1.29-1.12 (m, 12H, CH(C*H*$_3$)$_2$).

2.6 Catalytic reactions with styrene, indene, ethanol, isopropanol & *p*-CF_3 aniline

In a glovebox, a flame dried Schlenk equipped with a magnetic stirrer bar was charged with diazo compound **2-Ph** (52.4 mg, 0.2 mmol) and styrene, indene (0.205 mmol, 1.05 eq.), ethanol, isopropanol, *p*-CF_3 aniline (0.2 mmol, 1 eq.) in DCM (0.4 mL). Outside the glovebox, to this Schlenk was added a solution of complex 1 (0.01 mmol). The reaction was stirred for the appropriate amount of time. The yield was determined by $^{19}F\{^1H\}$ NMR spectroscopy using 4,4'-difluorobiphenyl as internal standard.

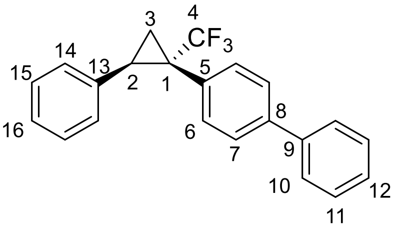

Cyclopropane 6: stirring for 24 h under static vacuum, 67% NMR yield. The pure product was obtained after column chromatography (Petroleum ether/Toluene : 9/1) as a white powder (25,5 mg, 38 %). **^1H NMR** (500 MHz, $CDCl_3$): δ 7.55-7.52 (m, 2H, H_{11}), 7.43-7.39 (m, 4H, H_7 & H_{10}), 7.36-7.31 (m, 1H, H_{12}), 7.24-7.20 (m, 2H, H_6), 7.13-7.09 (m, 3H, H_{15} & H_{16}), 6.96-6.82 (m, 2H, H_{14}), 2.89 (dd, J_{HH} = 9.7, 6.0 Hz, 1H, H_2), 1.93 (dd, J_{HH} = 9.7, 6.0 Hz, 1H, H_3), 1.73 (pseudo t, J_{HH} = 6.0 Hz, 1H, H'$_3$). **$^{13}C\{^1H\}$ NMR** (126 MHz, $CDCl_3$): δ 141.0 (C_8), 140.5 (C_9), 135.7 (C_{13}), 133.0 (C_6), 130.7 (C_5), 128.9 (C_7), 128.1 (C_{14}), 128.0 (C_{15}), 127.6 (C_{12}), 127.2 (C_{11}), 126.8 (C_{10}), 126.7 (C_{16}), 125.9 (q, $^1J_{CF}$ = 274,2 Hz, C_4), 35.6 (q, $^2J_{CF}$ = 32.7 Hz, C_1), 25.9 (q, $^3J_{CF}$ = 2.5 Hz, C_2), 14.9 (q, $^3J_{CF}$ = 2.5 Hz, C_3). **^{19}F NMR** (376 MHz, $CDCl_3$): δ -69.7 ppm (s). The diastereoselectivity has been confirmed by {^1H, ^{19}F} HOESY NMR. **EI-MS:** Calculated for $C_{22}H_{17}F_3$: 338.13. Found: 338.15. Crystals were grown by slow evaporation of DCM.

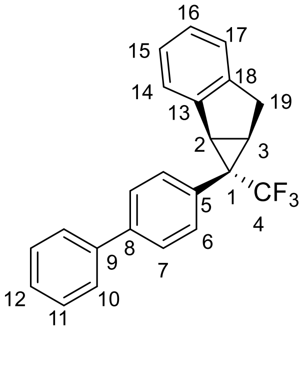

Cyclopropane 7: stirring for 24 h under static vacuum, 56% NMR yield. The pure product was obtained after column chromatography (Petroleum ether/Toluene : 9/1) as a white powder (27,1 mg, 39 %). **^1H NMR** (400 MHz, $CDCl_3$): δ 7.50-7.45 (m, 2H, H_{11}), 7.42 (d, J_{HH} = 7.6 Hz, 1H, H_{14}), 7.40-7.26 (m, 5H, H_7 & H_{10} & H_{12}), 7.16-7.07 (m, 3H, H_{15} & H_6), 6.92 (td, J_{HH} = 7.6, 1.2 Hz, 1H, H_{16}), 6.75 (d, J_{HH} = 7.6 Hz, 1H, H_{17}), 3.33 (d, J_{HH} = 6.8 Hz, 1H, H_2), 3.25 (dd, J_{HH} = 17.6, 6.8 Hz, 1H, H_{19}), 2.83 (d, J_{HH} = 17.6 Hz, 1H, H'$_{19}$), 2.65 (pseudo t, J_{HH} = 6.8 Hz, 1H, H_3). **$^{13}C\{^1H\}$ NMR** (101 MHz, $CDCl_3$): δ 142.3 (C_{18}), 140.6 (C_{13} & C_8), 140.4 (C_9), 133.4 (C_6), 128.8 (C_{10}), 128.0 (C_5), 127.4 (C_{12}), 127.1 (C_{11}), 126.7 (C_{16}), 126.5 (C_{15}), 126.4 (C_7), 125.9 (q, $^1J_{CF}$ = 274,4 Hz, C_4), 125.1 (C_{14}), 124.6 (C_{17}), 36.7 (q, $^2J_{CF}$ = 31.2 Hz, C_1), 33.8 (q, $^3J_{CF}$ = 3.0 Hz, C_2), 32.7 (C_{19}), 25.4 (q, $^3J_{CF}$ = 2.7 Hz, C_3). **^{19}F NMR** (376 MHz, $CDCl_3$): -68.6 ppm (s). The diastereoselectivity has been confirmed by {^1H, ^{19}F} HOESY NMR. **EI-MS:** Calculated for $C_{23}H_{17}F_3$: 350.13. Found: 350.27.

---

[6] M. Navarro, A. Toledo, M. Joost, A. Amgoune, S. Mallet-Ladeira, D. Bourissou, *Chem. Commun.* **2019**, *55*, 7974–7977.

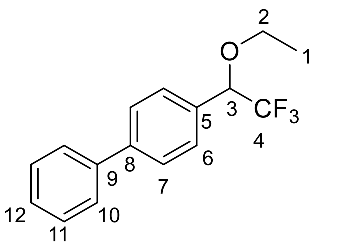

α-Trifluoromethyl ether (8): stirring for 2 h under flux of argon, 76% NMR yield The pure product was obtained after column chromatography (Petroleum ether/Ethyl Acetate : 95/5) as an oil (39 mg, 70 %). **^{1}H NMR** (300 MHz, $CDCl_3$): δ 7.67-7.59 (m, 4H, H_7 & H_{11}), 7.53 (d, J_{HH} = 8.0 Hz, 2H, H_6), 7.43 (m, 2H, H_{10}), 7.41-7.35 (m, 1H, H_{12}), 4.67 (q, $^{3}J_{HF}$ = 6.6 Hz, 1H, H_3), 3.63 (q, J_{HH} = 7.0 Hz, 2H, H_2), 1.29 (t, J_{HH} = 7.0 Hz, 3H, H_1). **^{13}C{^{1}H} NMR** (75 MHz, $CDCl_3$): δ 142.5 (C_8), 140.6 (C_9), 132.3 (C_5), 129.0 (C_{10}), 128.7 (C_6), 127.8 (C_{12}), 127.4 (C_{11}), 127.3 (C_7), 124.3 (q, $^{1}J_{CF}$ = 281,8 Hz, C_4), 79.6 (q, $^{2}J_{CF}$ = 31.0 Hz, C_3), 66.4 (C_2), 15.2 (C_1). **^{19}F NMR** (282 MHz, $CDCl_3$): δ -76.5 ppm (d, $^{3}J_{HF}$ = 6.6 Hz). **EI-MS:** Calculated for $C_{23}H_{17}F_3$: 280.11. Found: 280.27.

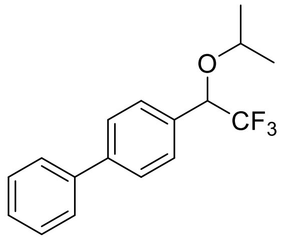

α-Trifluoromethyl ether (8'): stirring for 2 h under flux of argon, NMR yield: 54%. Analytical data are consistent with those previously reported:[7] **^{1}H NMR** (300 MHz, $CDCl_3$): δ 7.65-7.34 (m, 9H), 4.74 (q, J = 6.8 Hz, 1H), 3.75 (hept, J = 6.2 Hz, 1H), 1.27 (d, J = 6.2 Hz, 3H), 1.19 (d, J = 6.2 Hz, 3H). **^{19}F NMR** (282 MHz, $CDCl_3$): δ -76.8 ppm (d, $^{3}J_{HF}$ = 6.8 Hz).

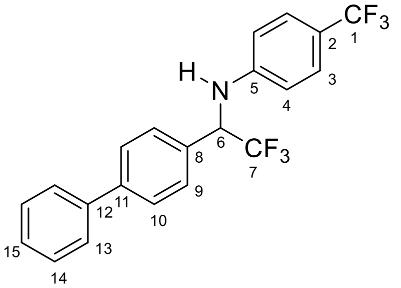

α-Trifluoromethyl amine (8''): stirring for 16 h in a glovebox, 76% NMR yield. The pure product was obtained after column chromatography (Pentane/Ethyl Acetate: 95/5) as an oil (51.5 mg, 65 %). **^{1}H NMR** (300 MHz, $CDCl_3$): δ 7.70-7.66 (m, 2H, H_{10}), 7.65-7.60 (m, 2H, H_{14}), 7.59-7.54 (m, 2H, H_9), 7.53-7.45 (m, 4H, H_3 and H_{13}), 7.45-7.40 (M, 1H, H_{15}) 6.77-6.51 (d, 2H, $^{3}J_{HH}$ = 8.8 Hz, H_4), 5.02 (dq, $^{3}J_{HF}$ = 7.2 Hz, $^{3}J_{HH}$ = 7.3 Hz, 1H, H_6) 4.68 (d, $^{2}J_{HH}$ = 7.3 Hz, 1H, NH). **^{13}C{^{1}H} NMR** (75 MHz, $CDCl_3$): δ 148.1 (C_5), 142.6 (C_{12}), 140.2 (C_{11}), 132.2 (C_8), 129.0 (C_{10}), 128.4 (C_9), 128.0 (C_{13}), 127.9 (C_{15}), 127.3 (C_{14}), 126.9 (q, $^{3}J_{CF}$ = 3.8 Hz, C_3), 125.0 (q, $^{1}J_{CF}$ = 282.1 Hz, C_7), 124.7 (q, $^{1}J_{CF}$ = 270.7 Hz, C_1), 121.1 (q, $^{2}J_{CF}$ = 33.2 Hz, C_2), 113.3 (C_4), 59.9 (q, $^{2}J_{CF}$ = 30.3 Hz, C_6). δ **^{19}F NMR** (282 MHz, $CDCl_3$): δ -61.4 (s, 3F, CF_3), -73.9 (d, $^{3}J_{HF}$ = 7.2 Hz, 3F, CF_3).

---

[7] C. Kuang, X. Zhou, Q. Xie, C. Ni, Y. Gu, J. Hu, *Org. Lett.* **2020**, *22*, 8670–8675.

3. NMR spectra

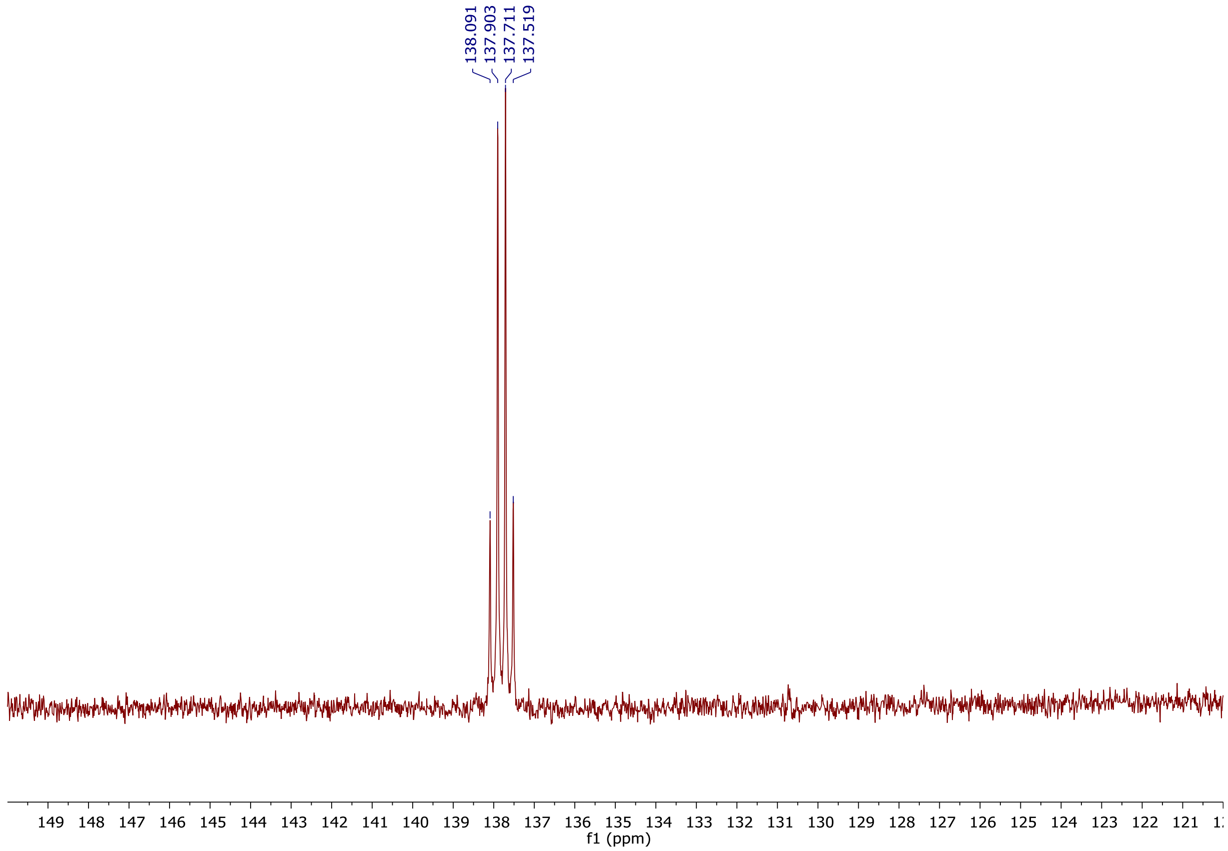

Figure S1. $^{31}P\{^{1}H\}$ NMR spectrum of (**3-Ph**) in CD_2Cl_2.

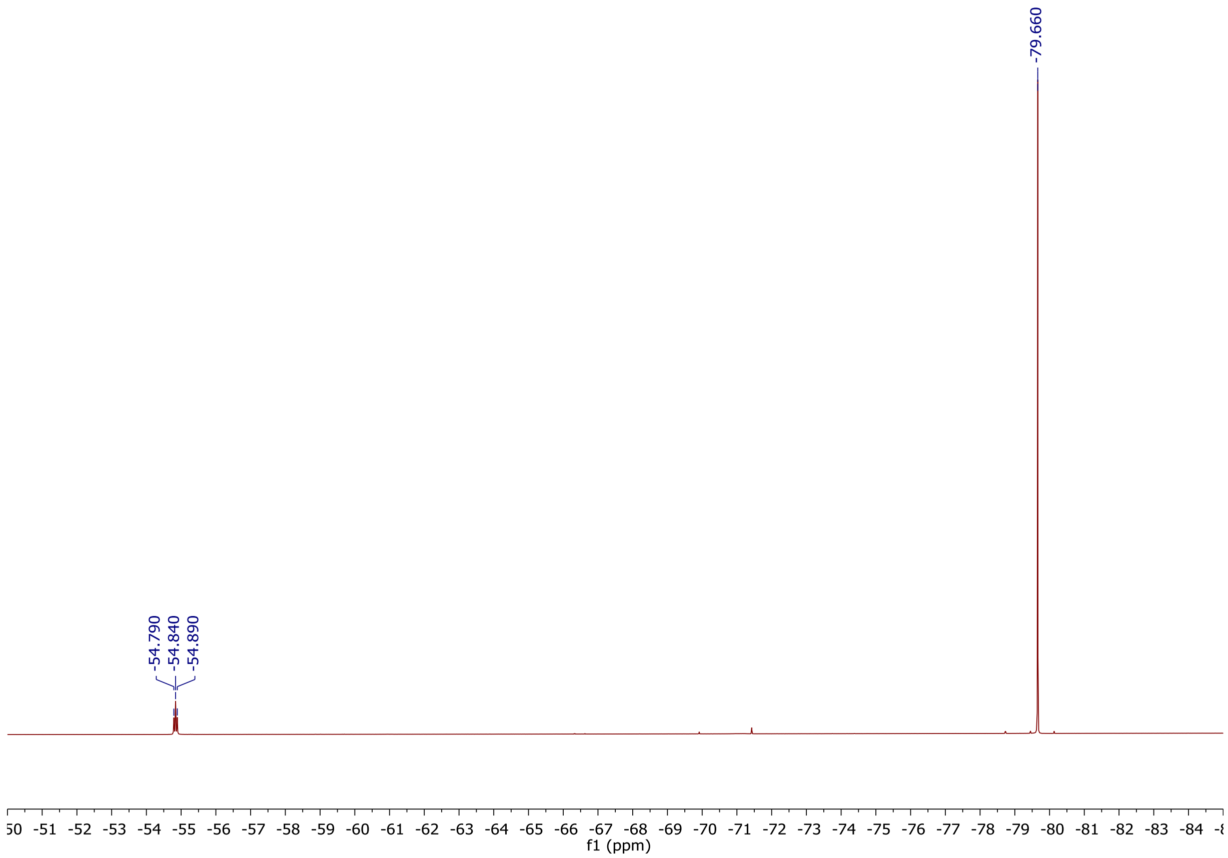

Figure S2. ^{19}F NMR spectrum of (**3-Ph**) in CD_2Cl_2.

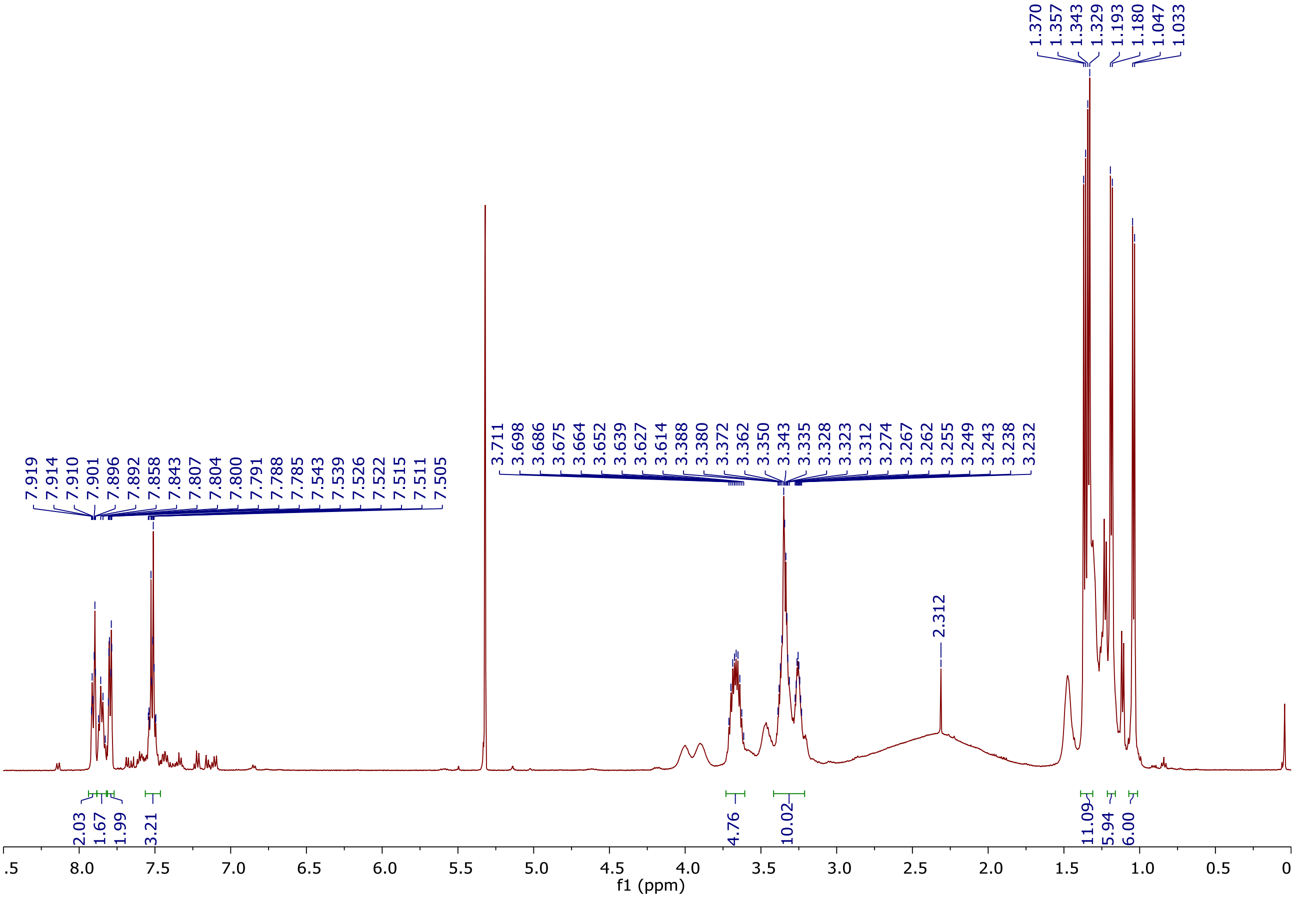

Figure S3. ^{1}H NMR spectrum of (**3-Ph**) in CD_2Cl_2 at 243 K.

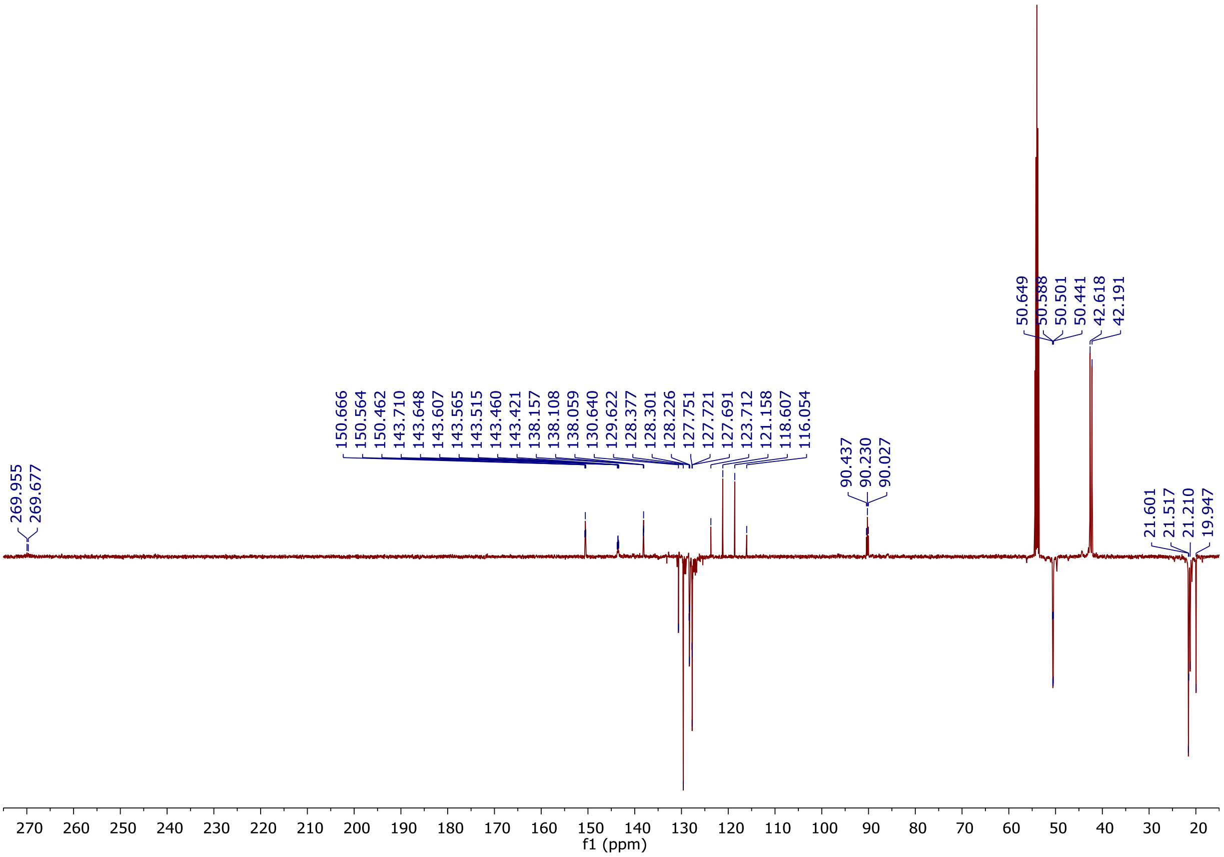

Figure S4. $^{13}C\{^{1}H\}_{jmod}$ NMR spectrum of (**3-Ph**) in CD_2Cl_2.

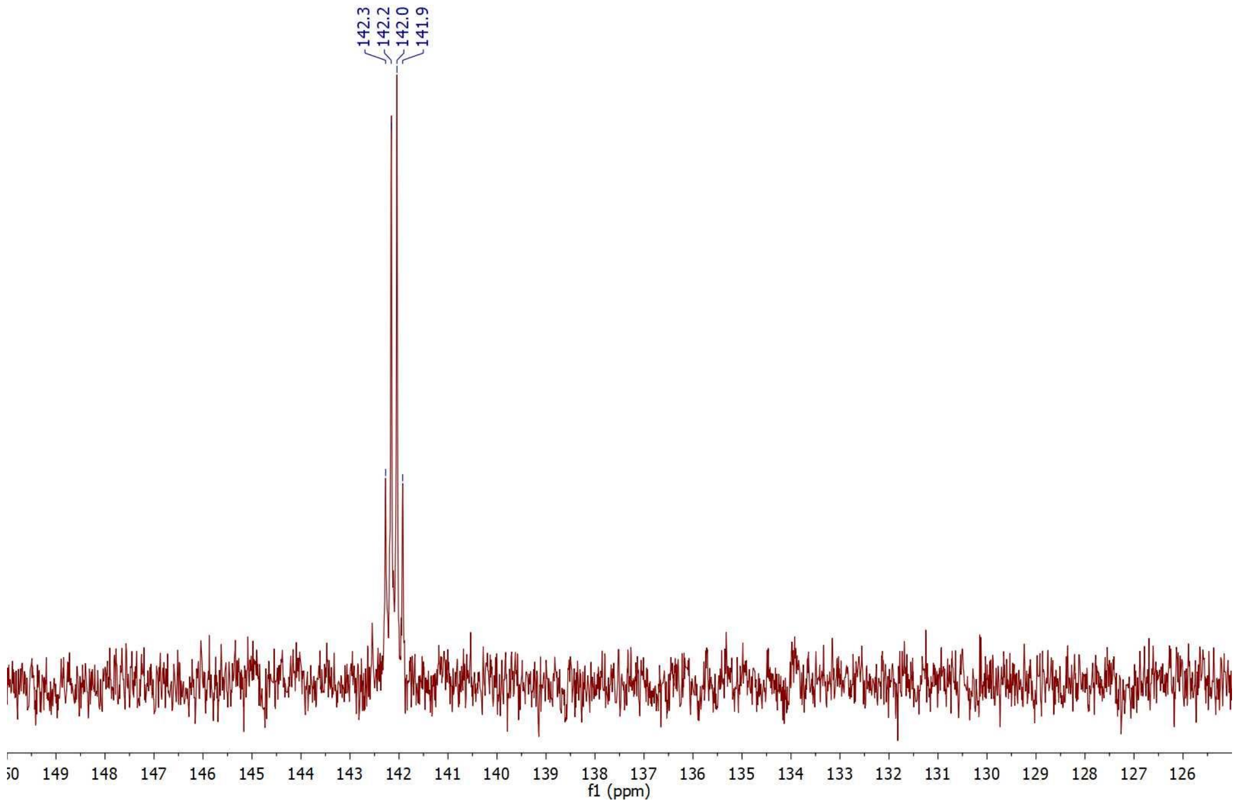

Figure S5. $^{31}P\{^{1}H\}$ NMR spectrum of (**3-OMe**) in $CDCl_3$.

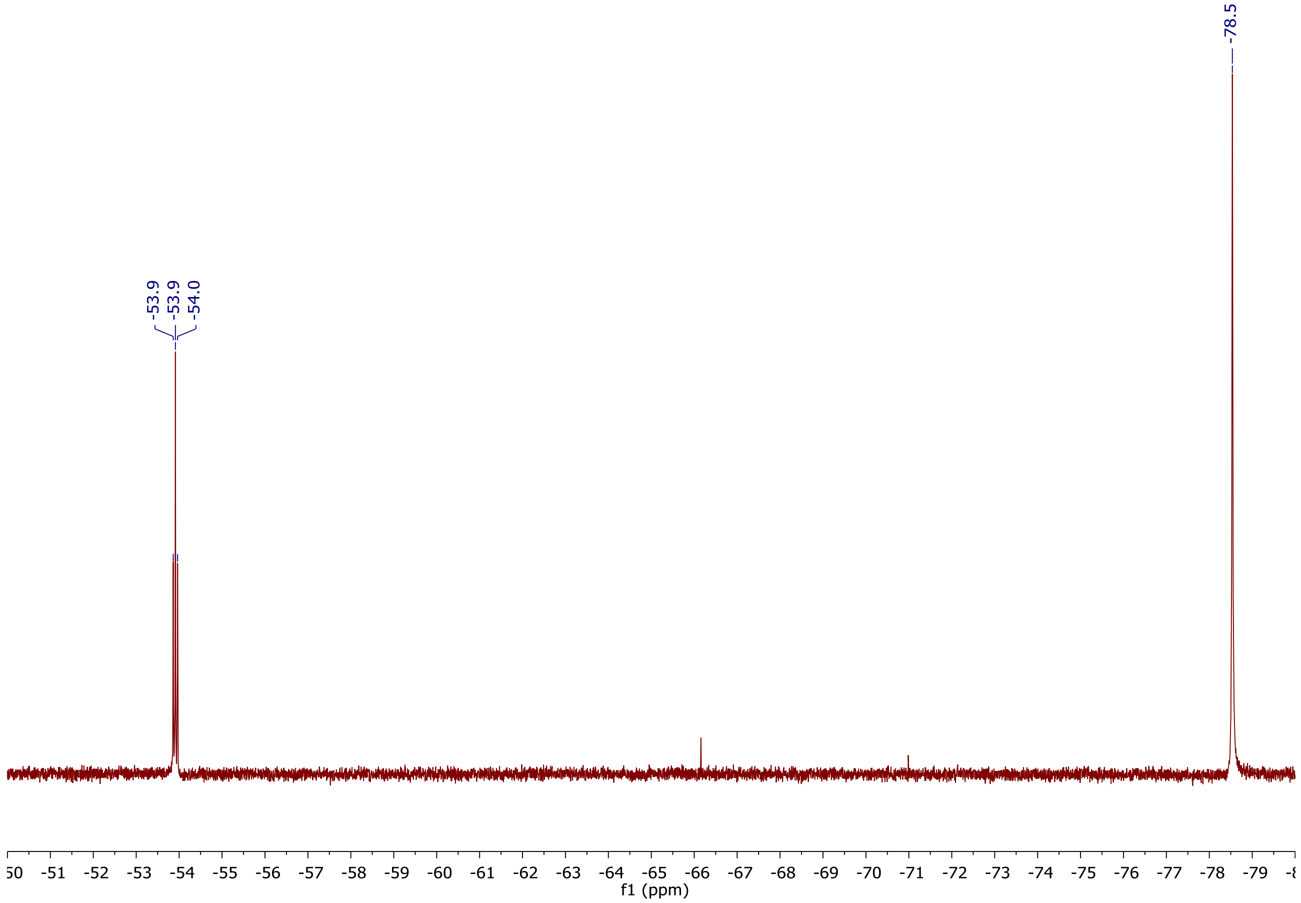

Figure S6. $^{19}F\{^{1}H\}$ NMR spectrum of (**3-OMe**) in $CDCl_3$.

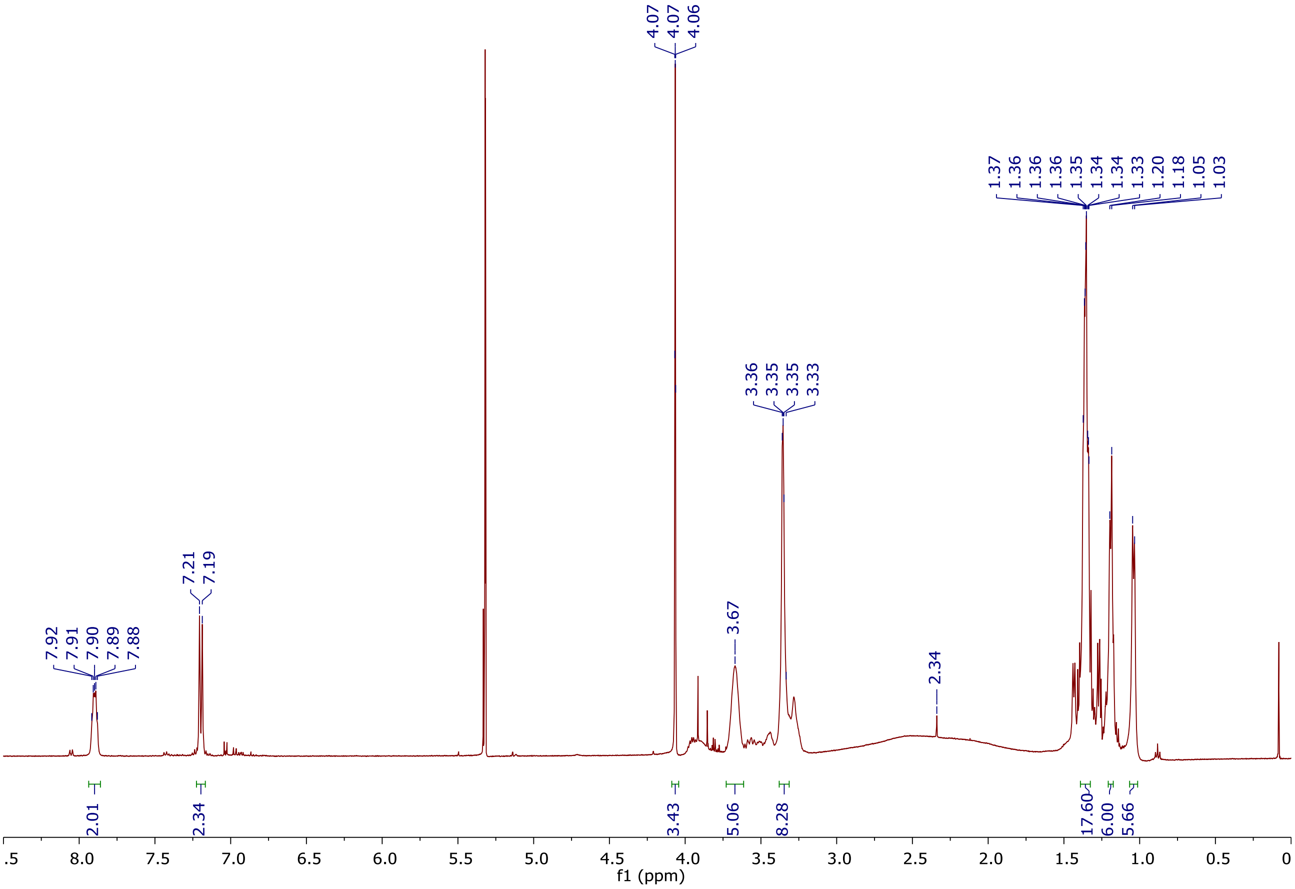

Figure S7. ^{1}H NMR spectrum of (**3-OMe**) in CD_2Cl_2.

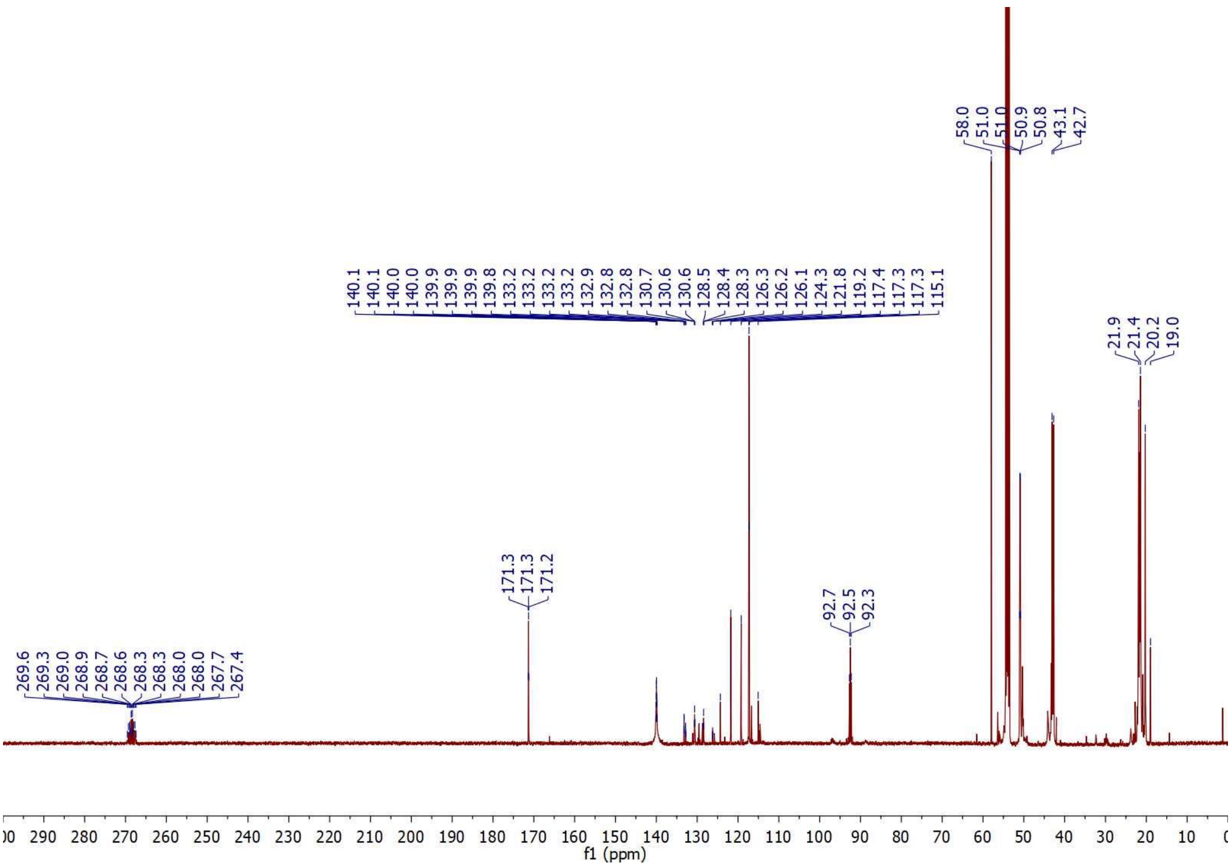

Figure S8. $^{13}C\{^{1}H\}$ NMR spectrum of (**3-OMe**) in CD_2Cl_2.

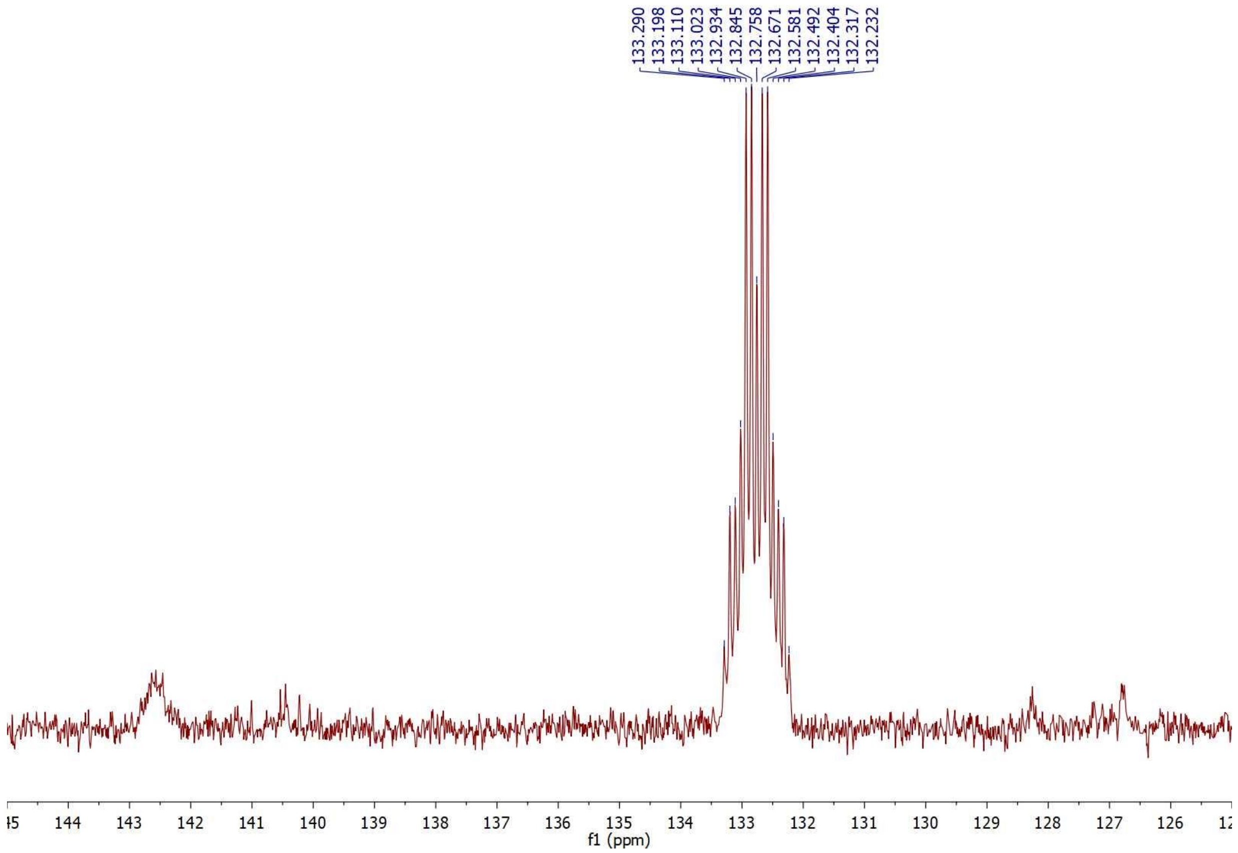

Figure S9. $^{31}P\{^{1}H\}$ NMR spectrum of (**3-CF$_3$**) in CD_2Cl_2.

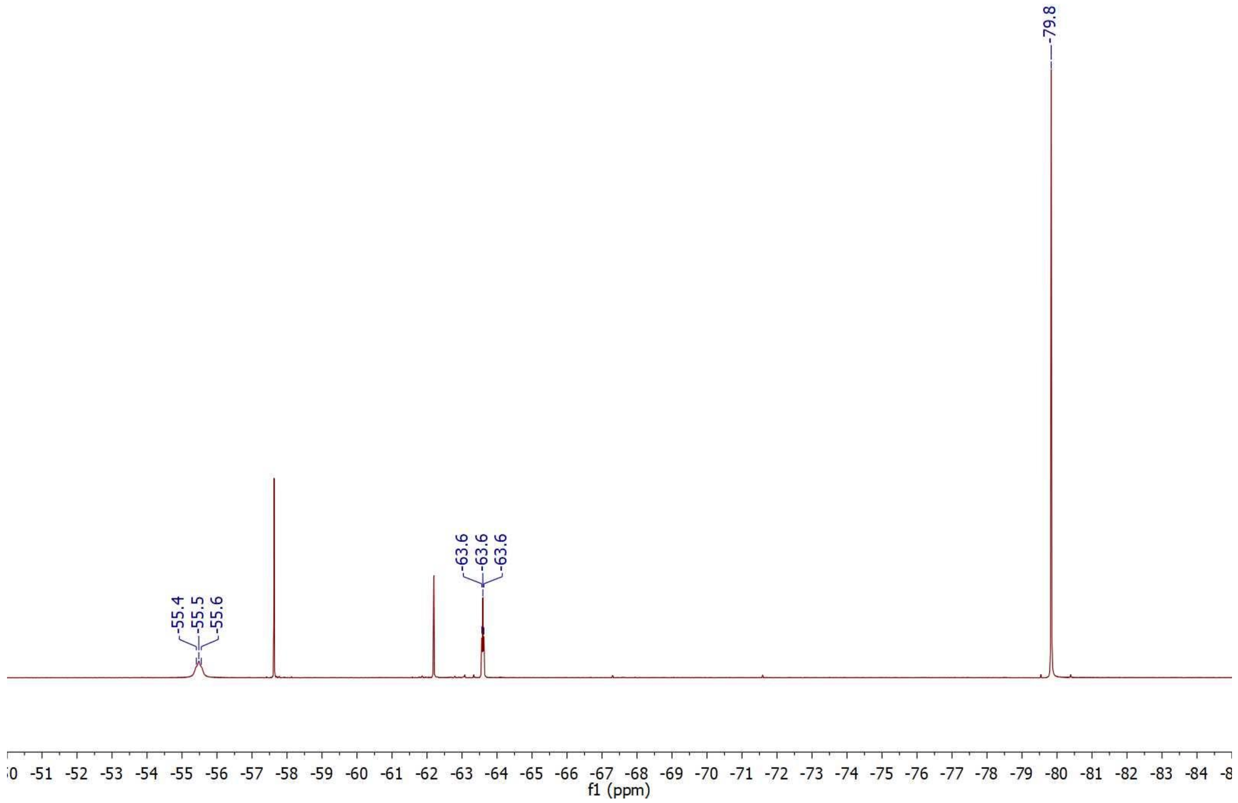

Figure S10. $^{19}F\{^{1}H\}$ NMR spectrum of (**3-CF$_3$**) in CD_2Cl_2 at 213 K.

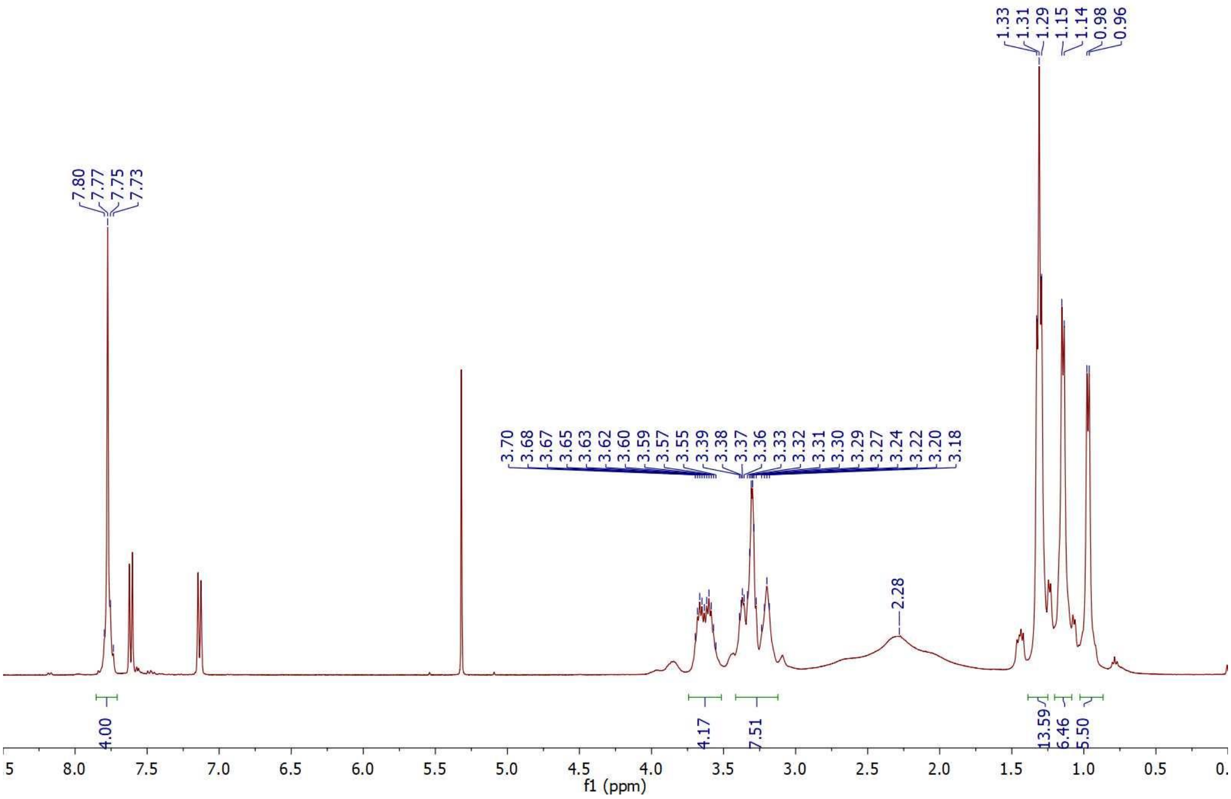

Figure S11. ^{1}H NMR spectrum of (**3-CF₃**) in CD_2Cl_2 at 213 K.

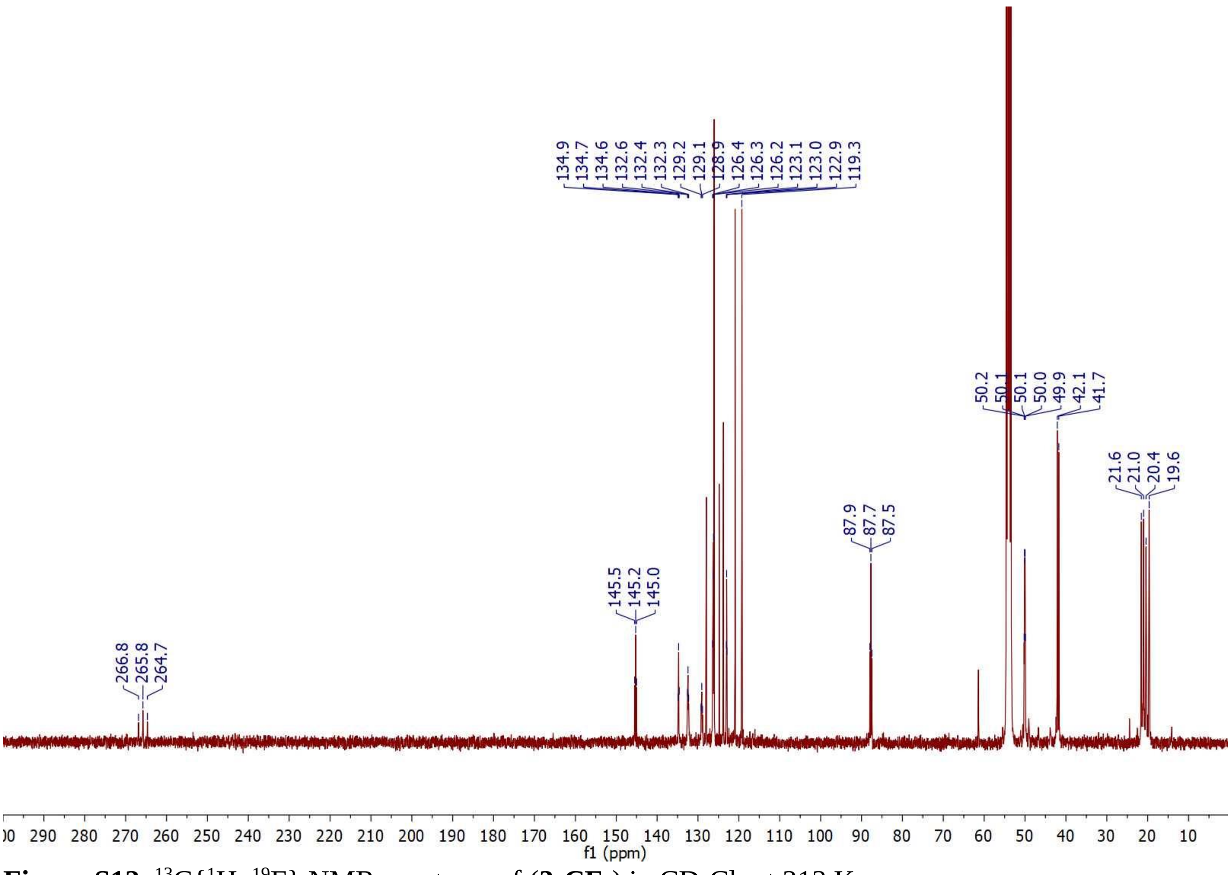

Figure S12. $^{13}C\{^{1}H, ^{19}F\}$ NMR spectrum of (**3-CF₃**) in CD_2Cl_2 at 213 K.

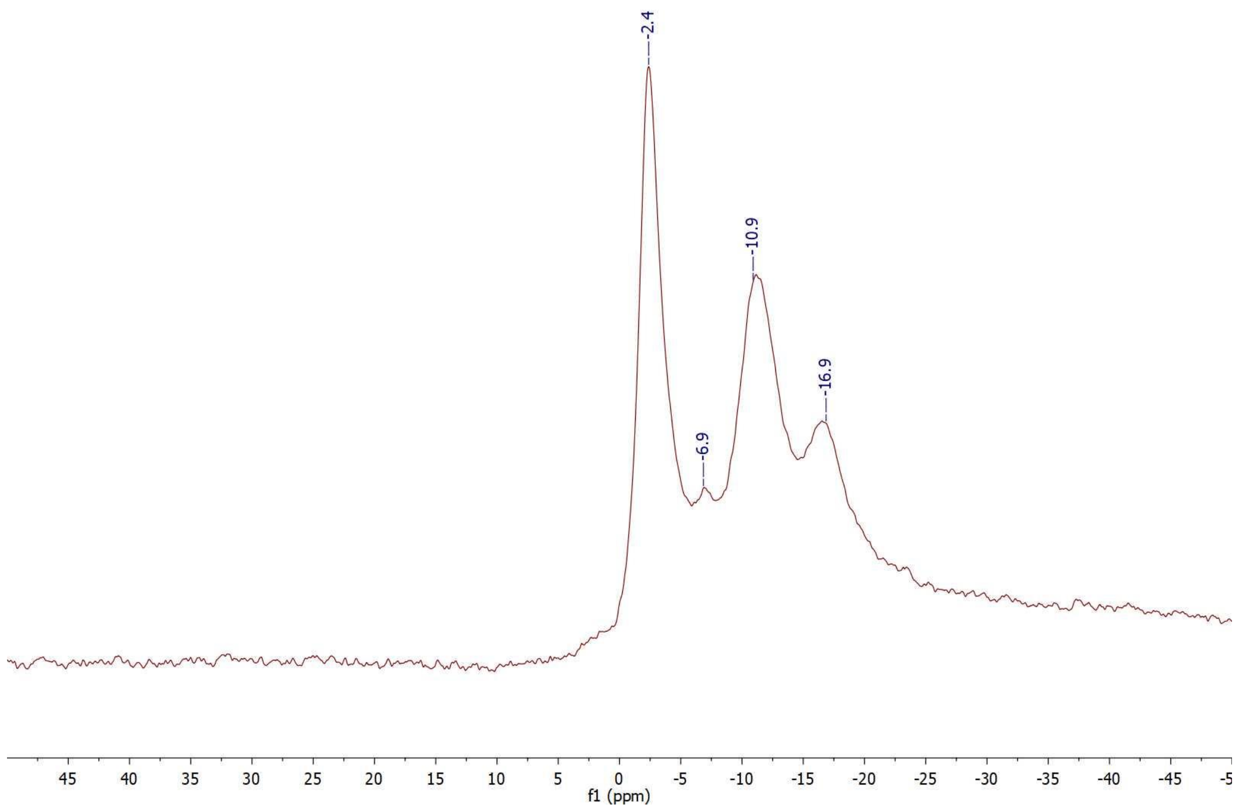

Figure S13. $^{11}B\{^{1}H\}$ NMR spectrum of (**3-CF$_3$**) in CD_2Cl_2.

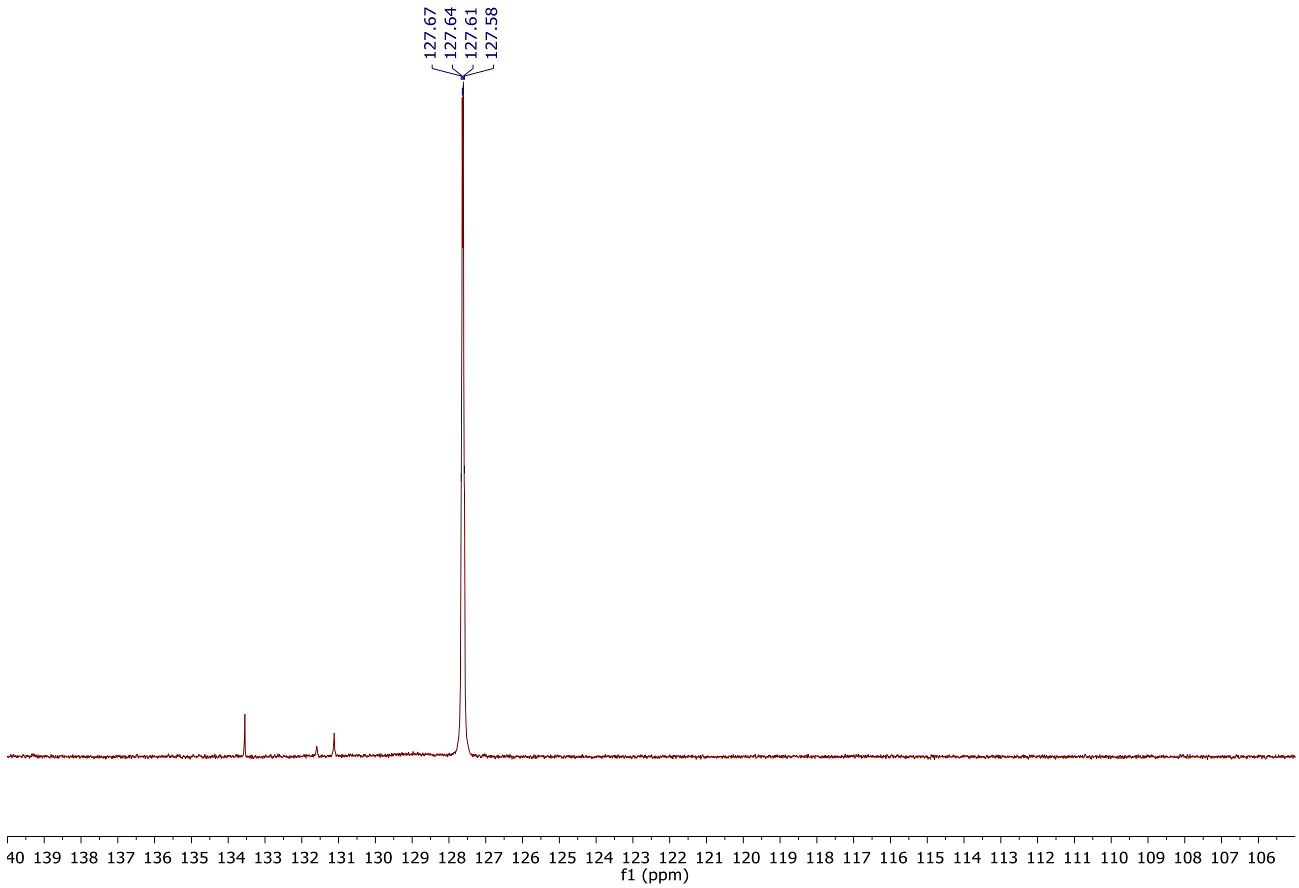

Figure S14. $^{31}P\{^{1}H\}$ NMR spectrum of (**4**) in $CDCl_3$.

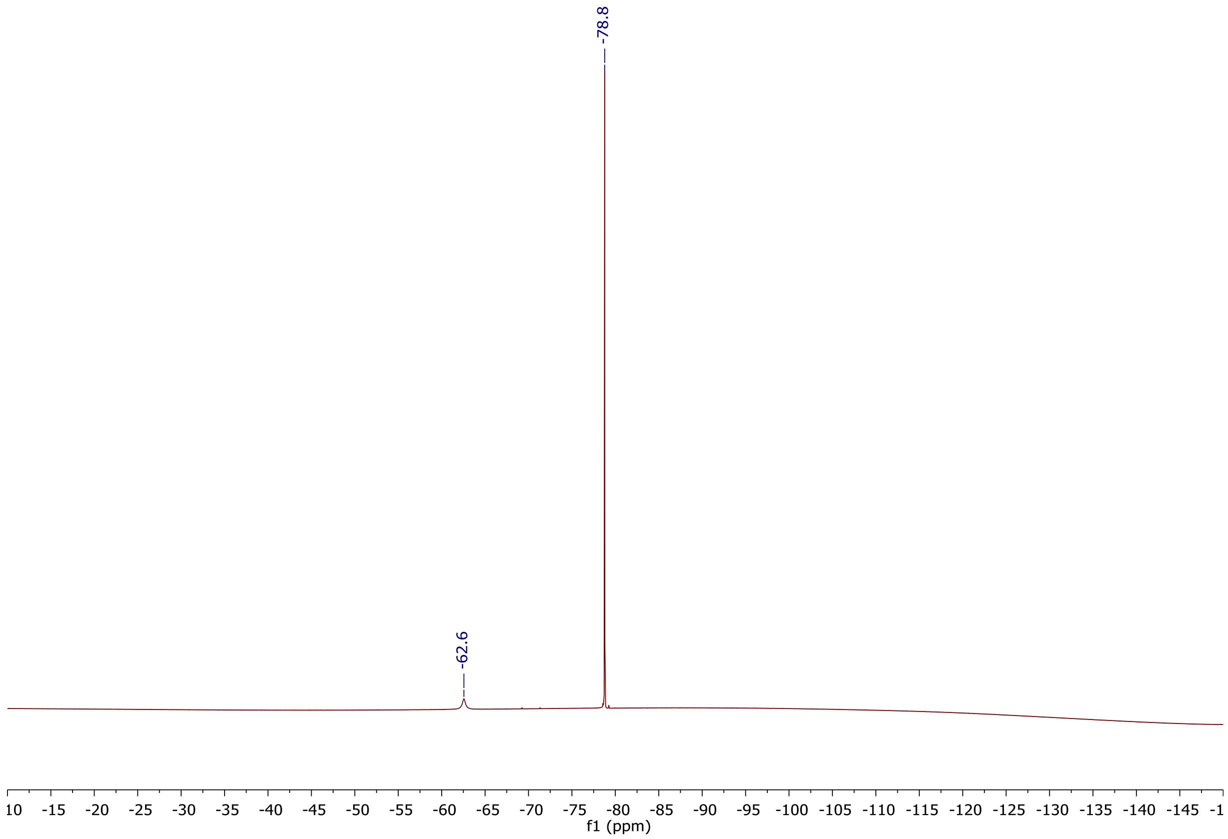

Figure S15. ^{19}F NMR spectrum of (**4**) in $CDCl_3$.

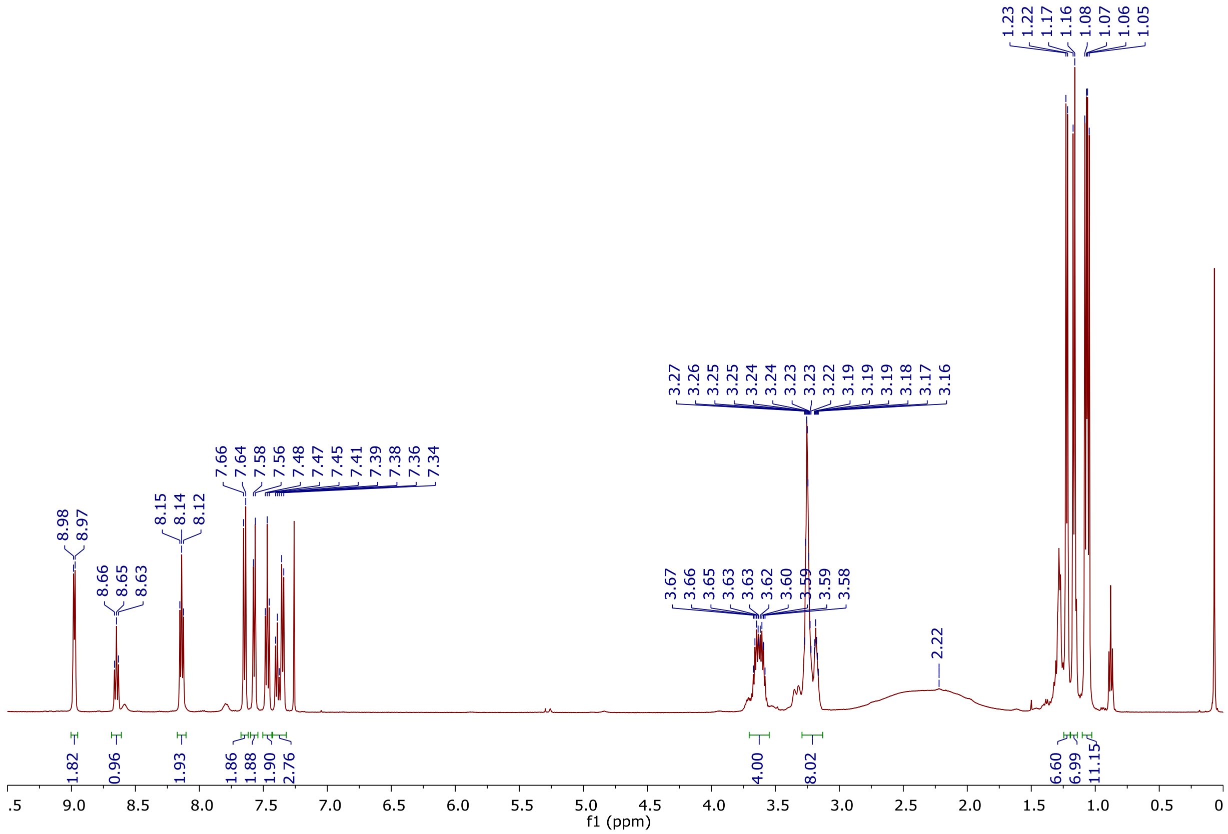

Figure S16. ^{1}H NMR spectrum of (**4**) in $CDCl_3$.

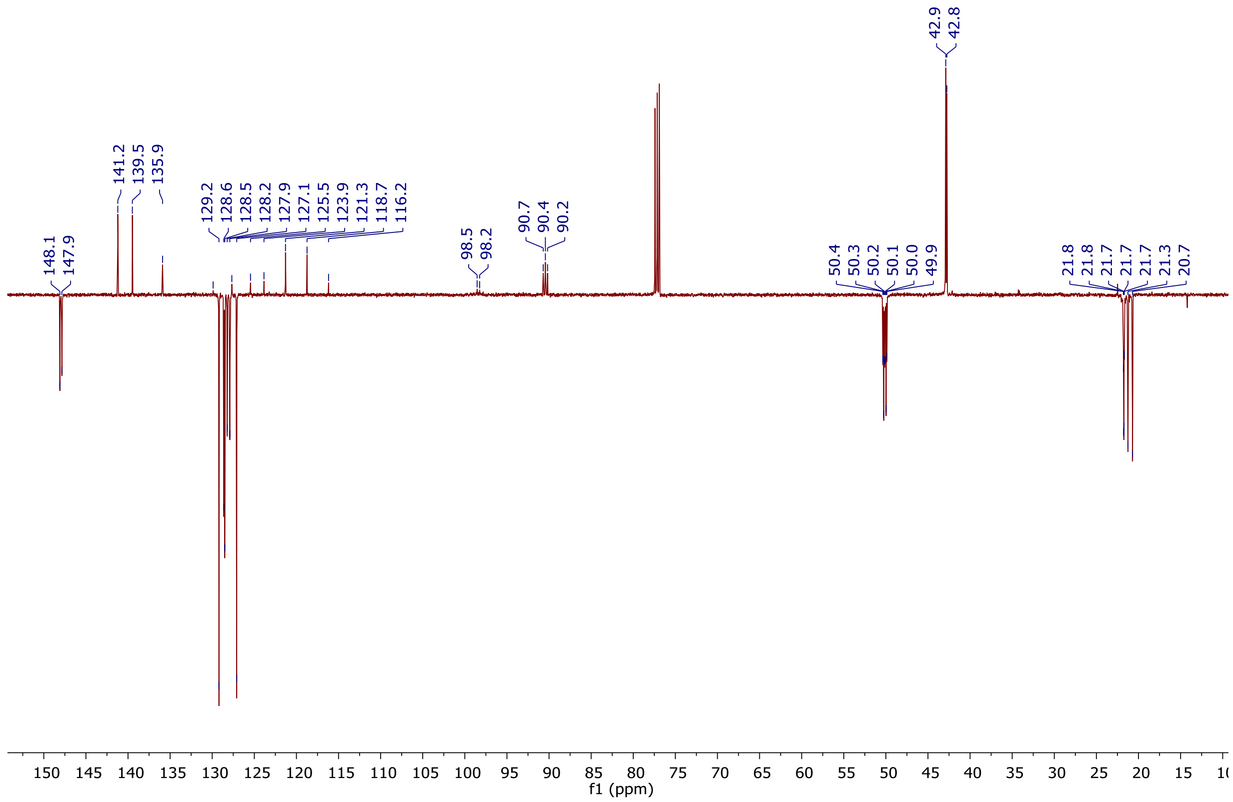

Figure S17. $^{13}C\{^{1}H\}_{jmod}$ NMR spectrum of (**4**) in $CDCl_3$.

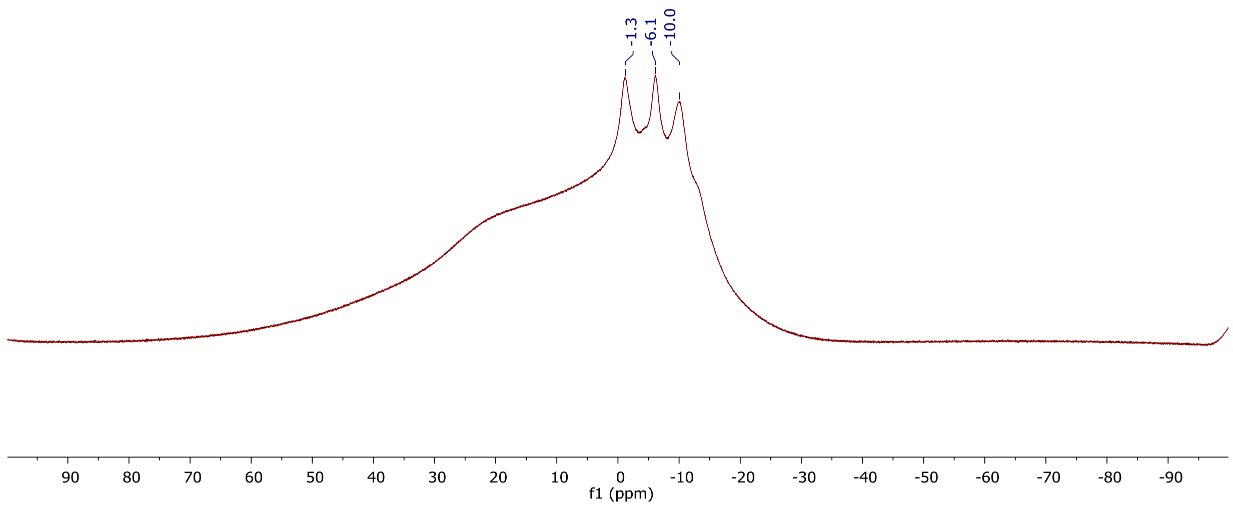

Figure S18. $^{11}B\{^{1}H\}$ NMR spectrum of (**4**) in $CDCl_3$.

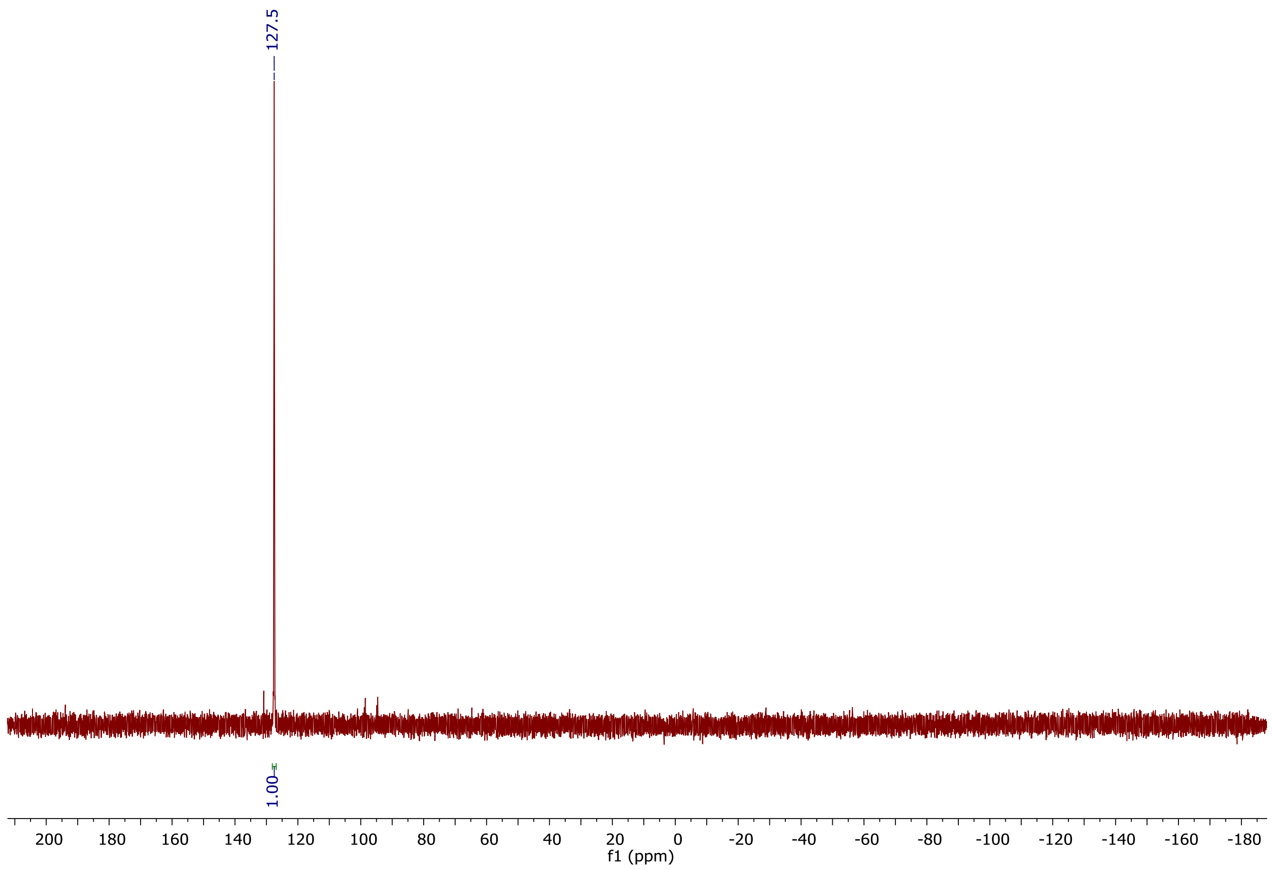

Figure S19. $^{31}P\{^{1}H\}$ NMR spectrum of (**5**) in $CDCl_3$.

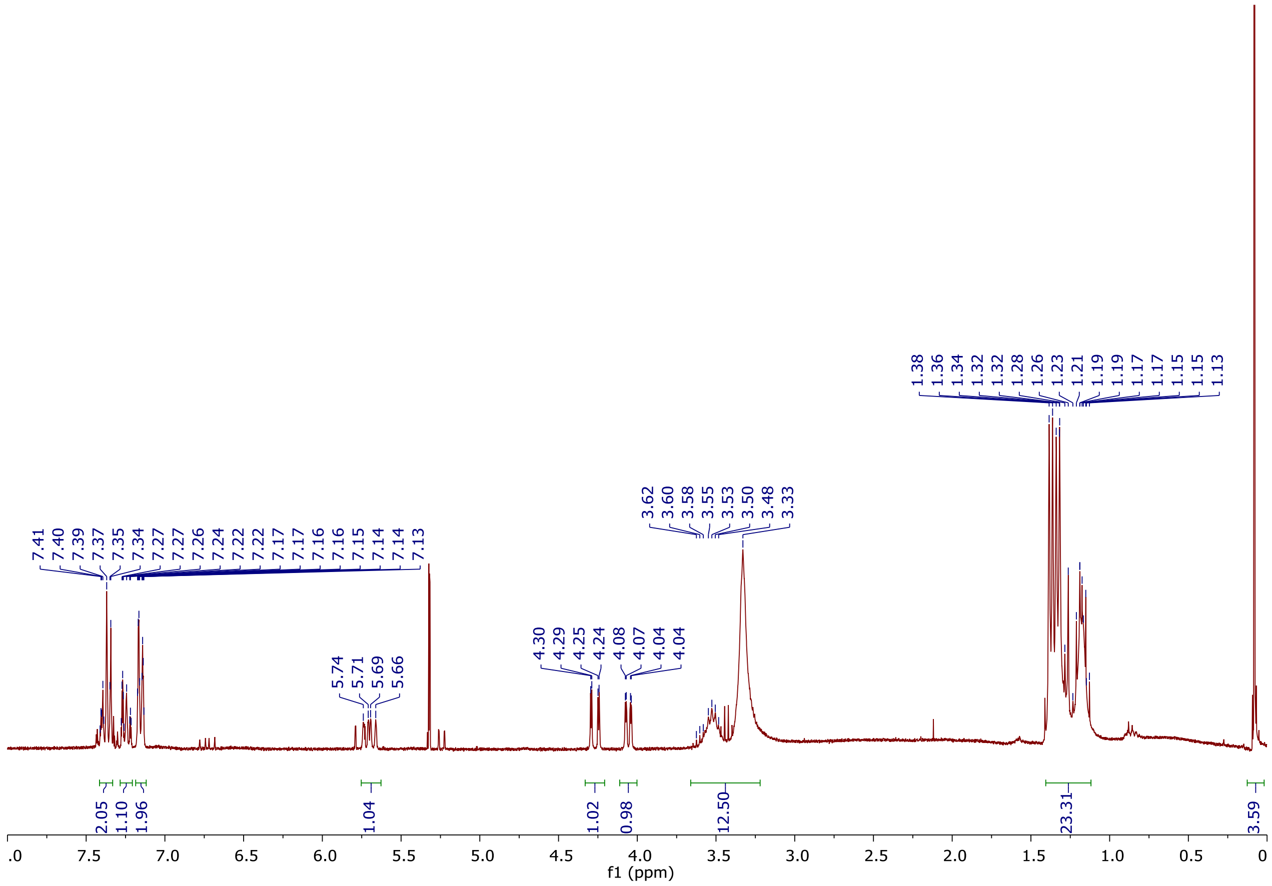

Figure S20. ^{1}H NMR spectrum of (**5**) in $CDCl_3$.

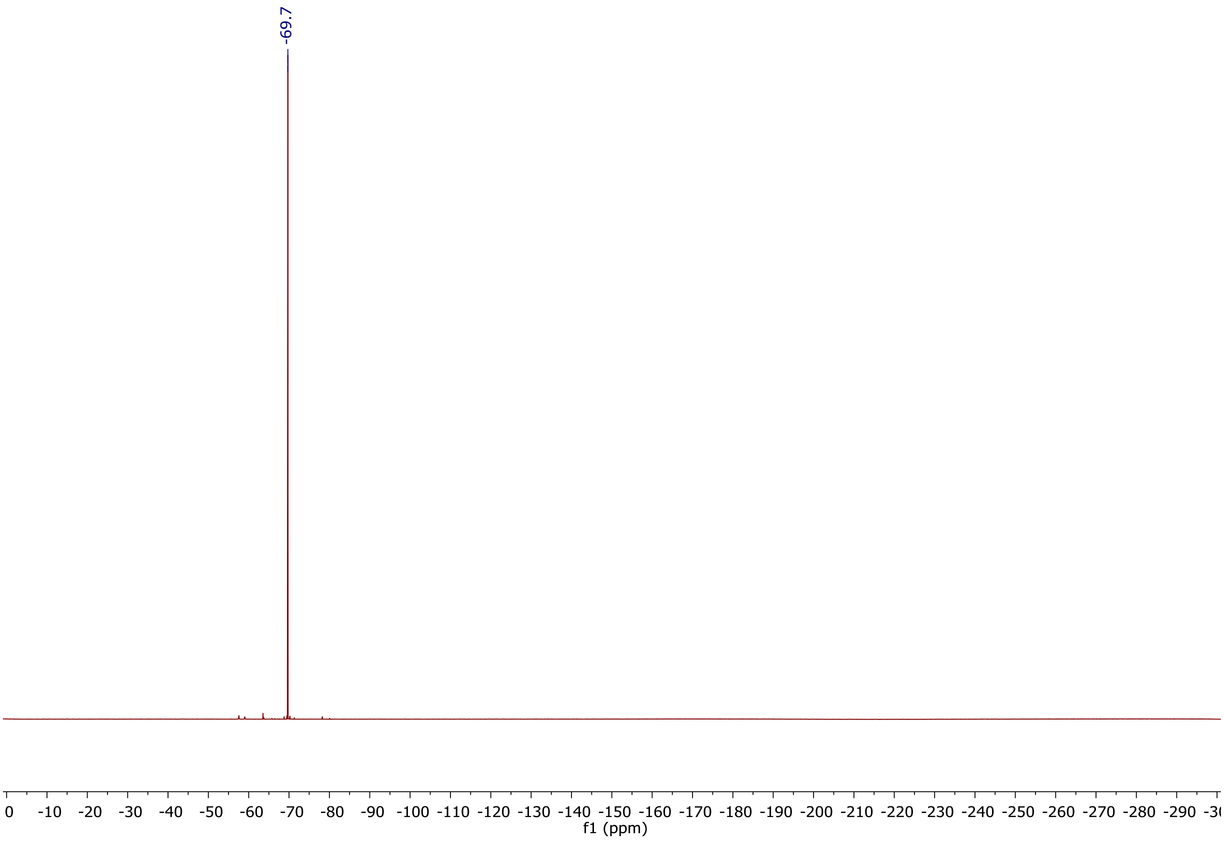

Figure S21. ^{19}F NMR spectrum of (**6**) in $CDCl_3$.

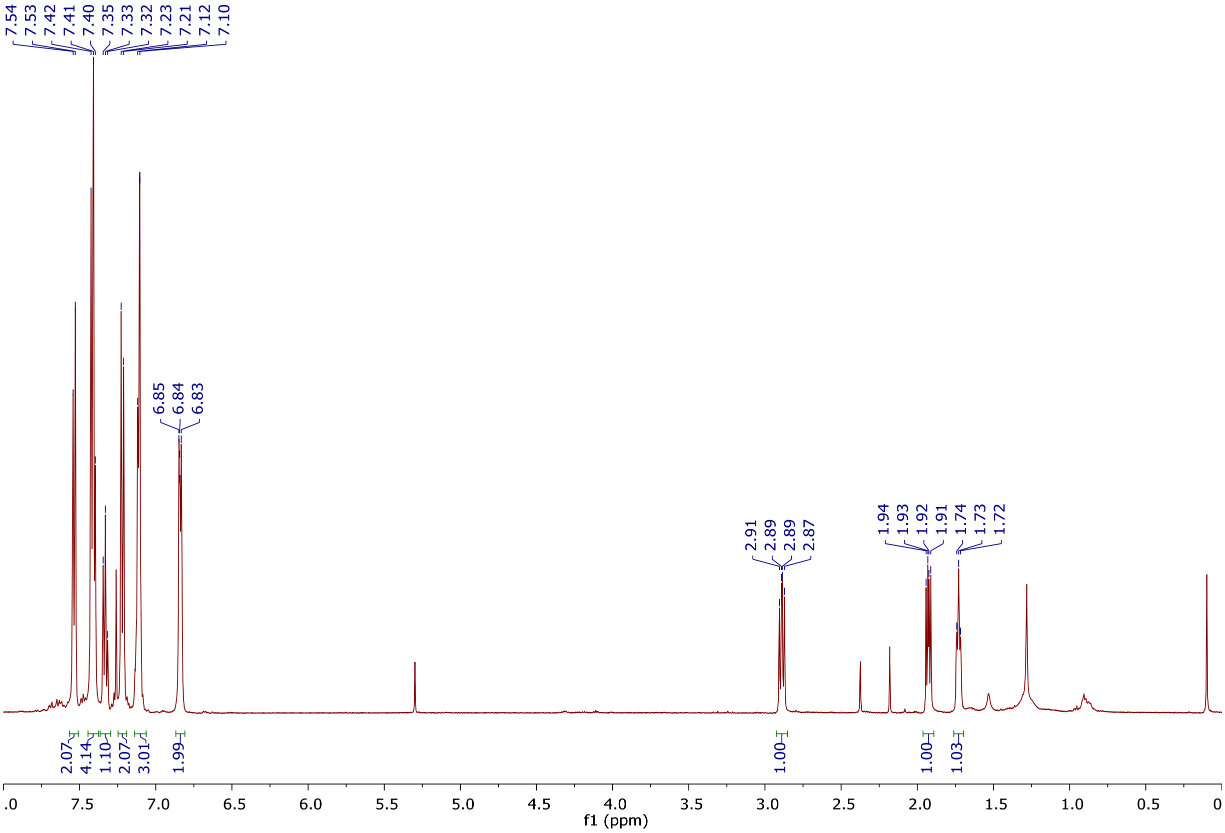

Figure S22. ^{1}H NMR spectrum of (**6**) in $CDCl_3$.

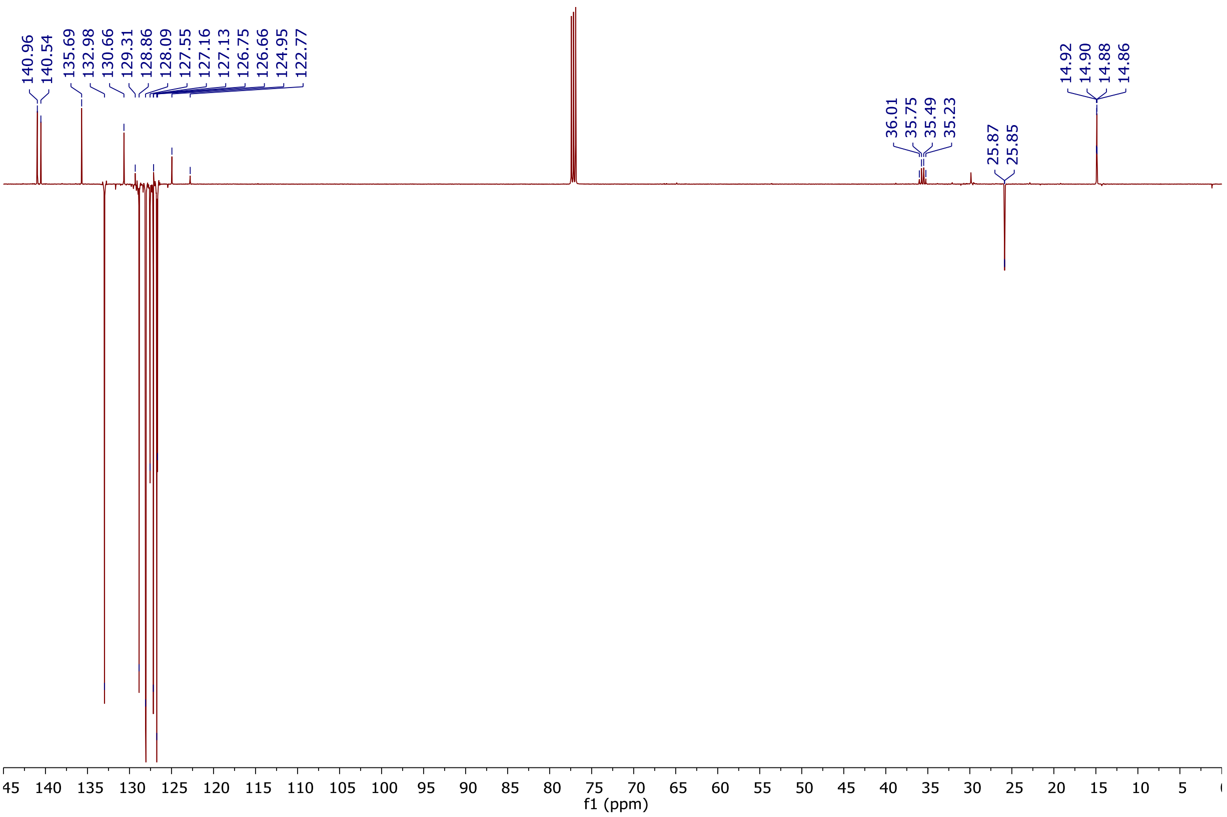

Figure S23. $^{13}C\{^{1}H\}_{jmod}$ NMR spectrum of (**6**) in $CDCl_3$.

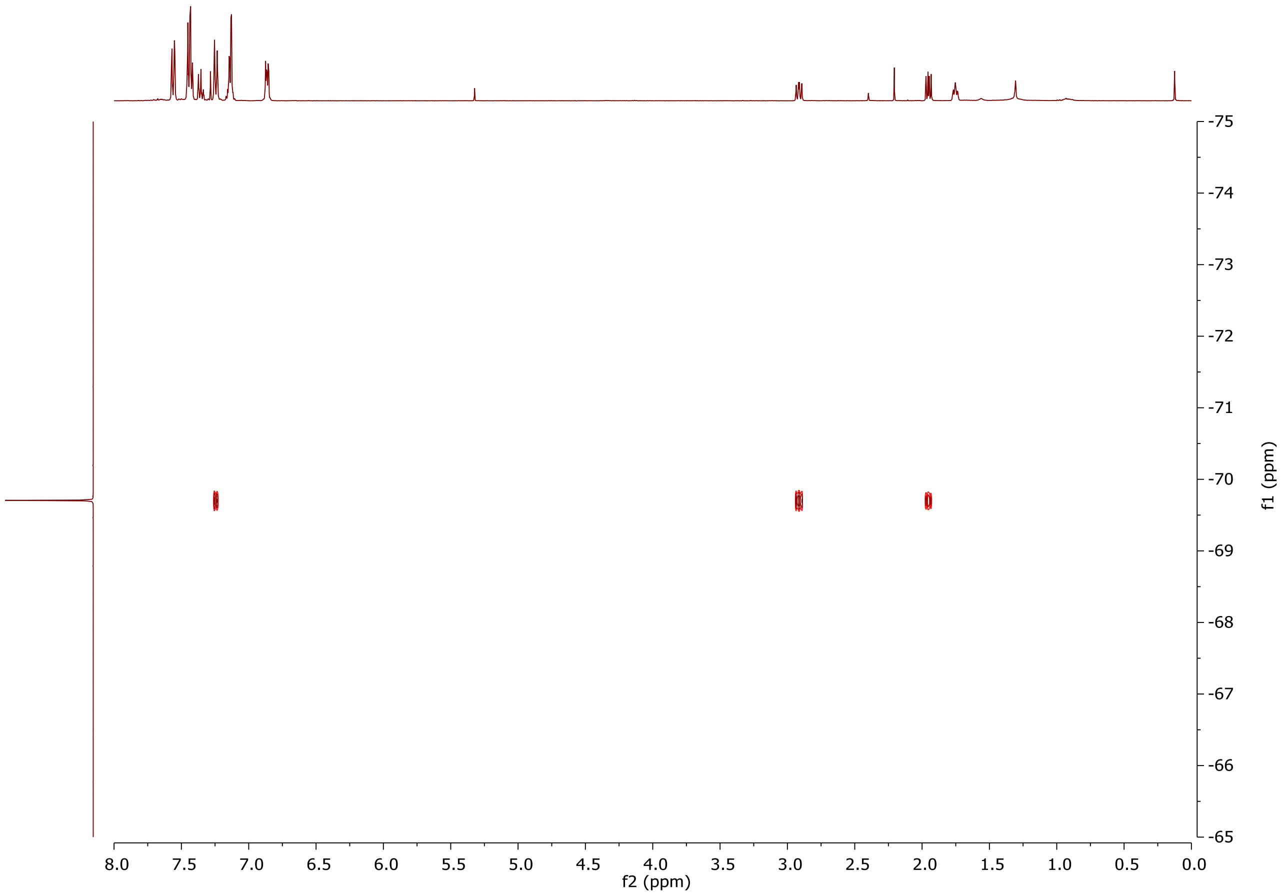

Figure S24. $\{^{1}H,^{19}F\}$ HOESY NMR spectrum of (**6**) in $CDCl_3$.

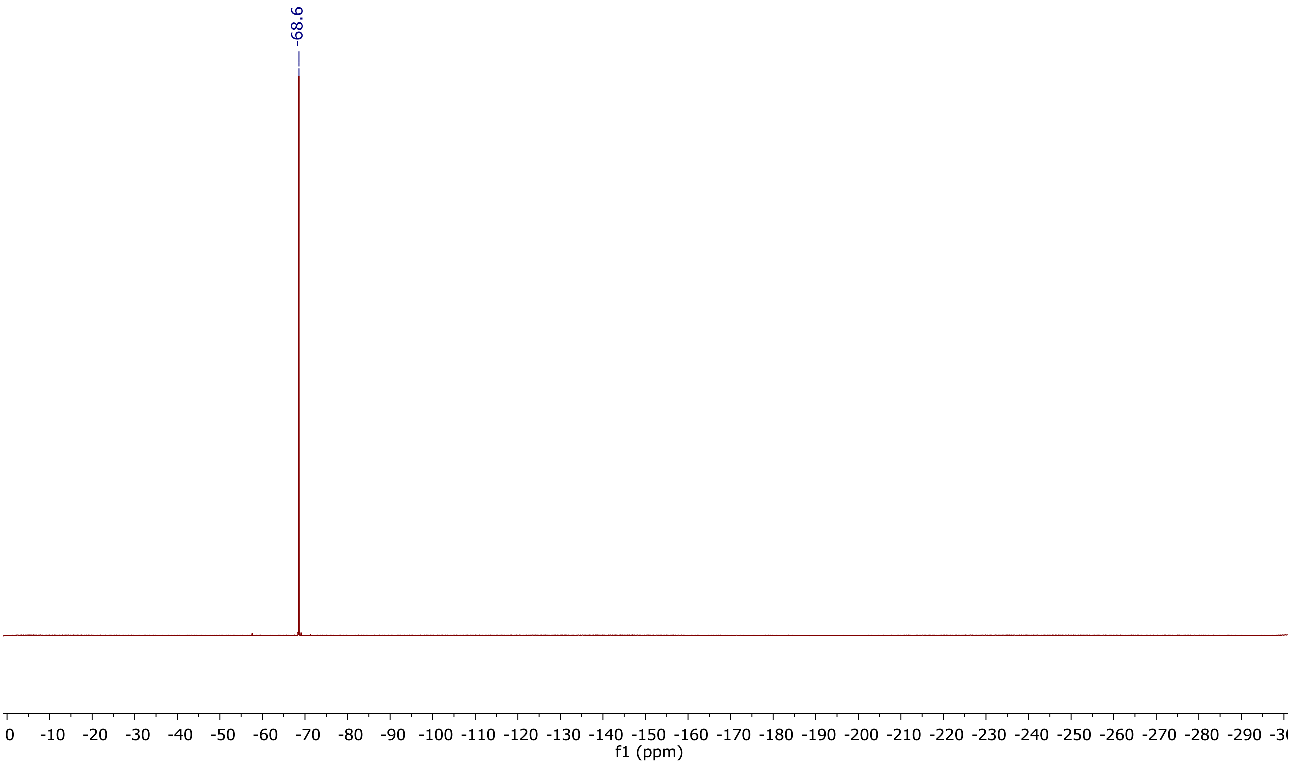

Figure S25. ^{19}F NMR spectrum of (**7**) in $CDCl_3$.

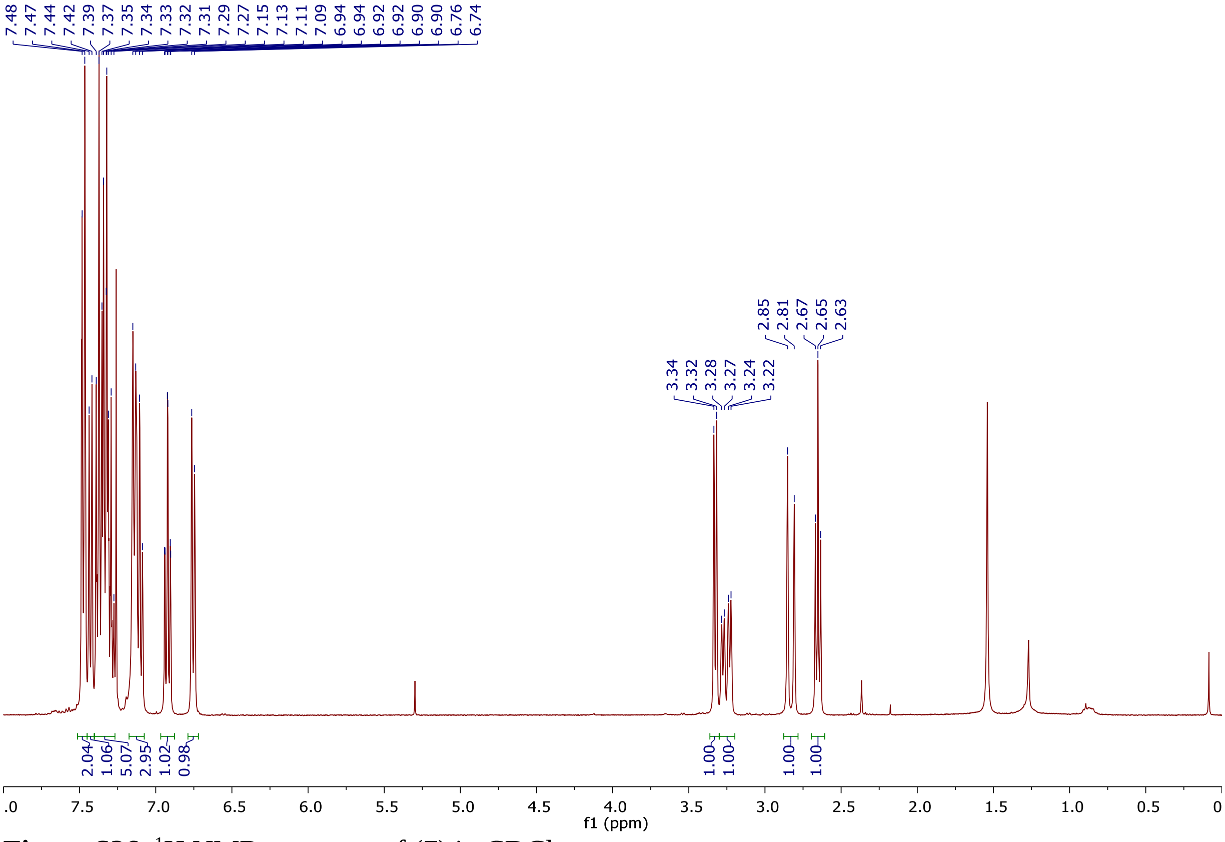

Figure S26. ^{1}H NMR spectrum of (**7**) in $CDCl_3$.

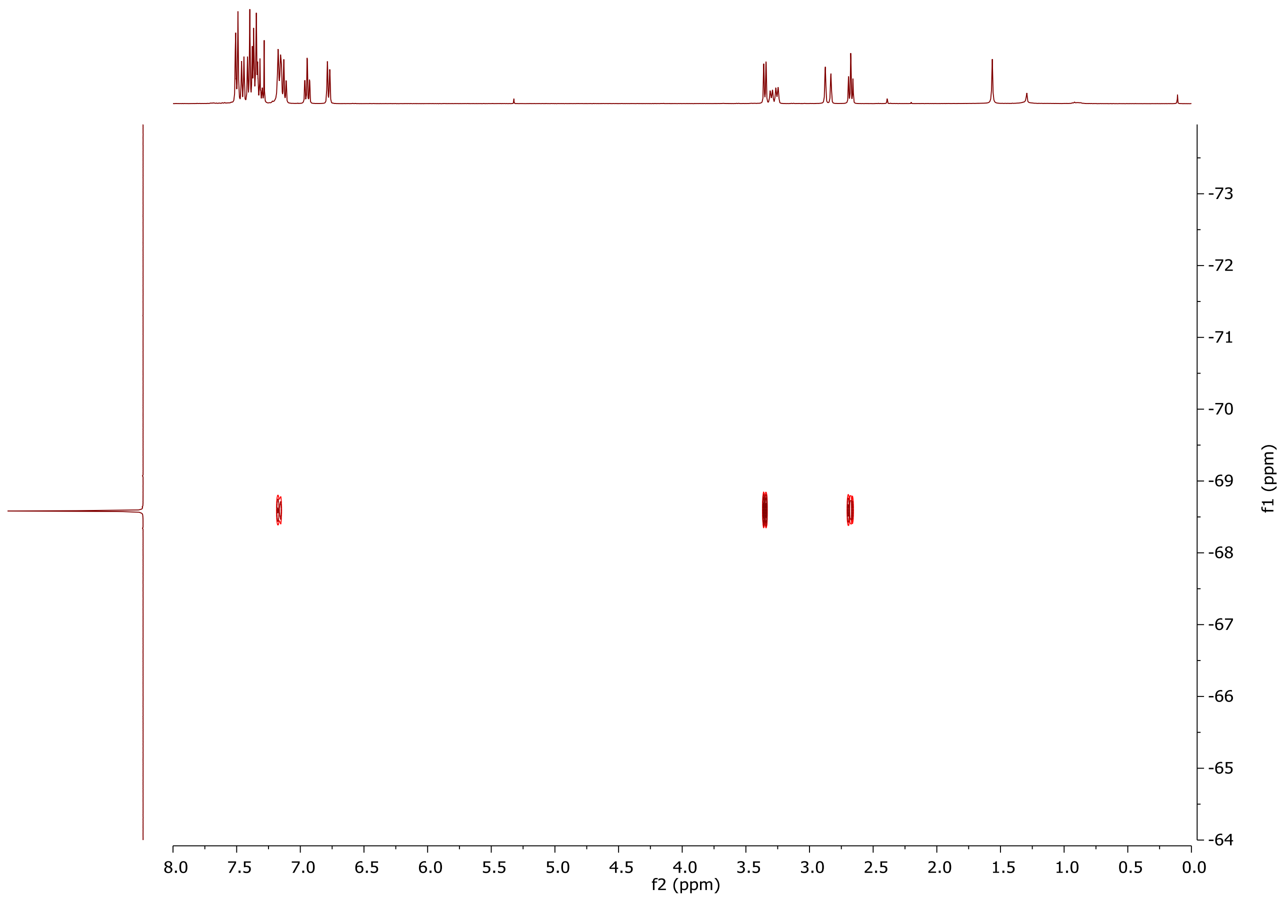

Figure S27. {^{1}H,^{19}F} HOESY NMR spectrum of (**7**) in $CDCl_3$.

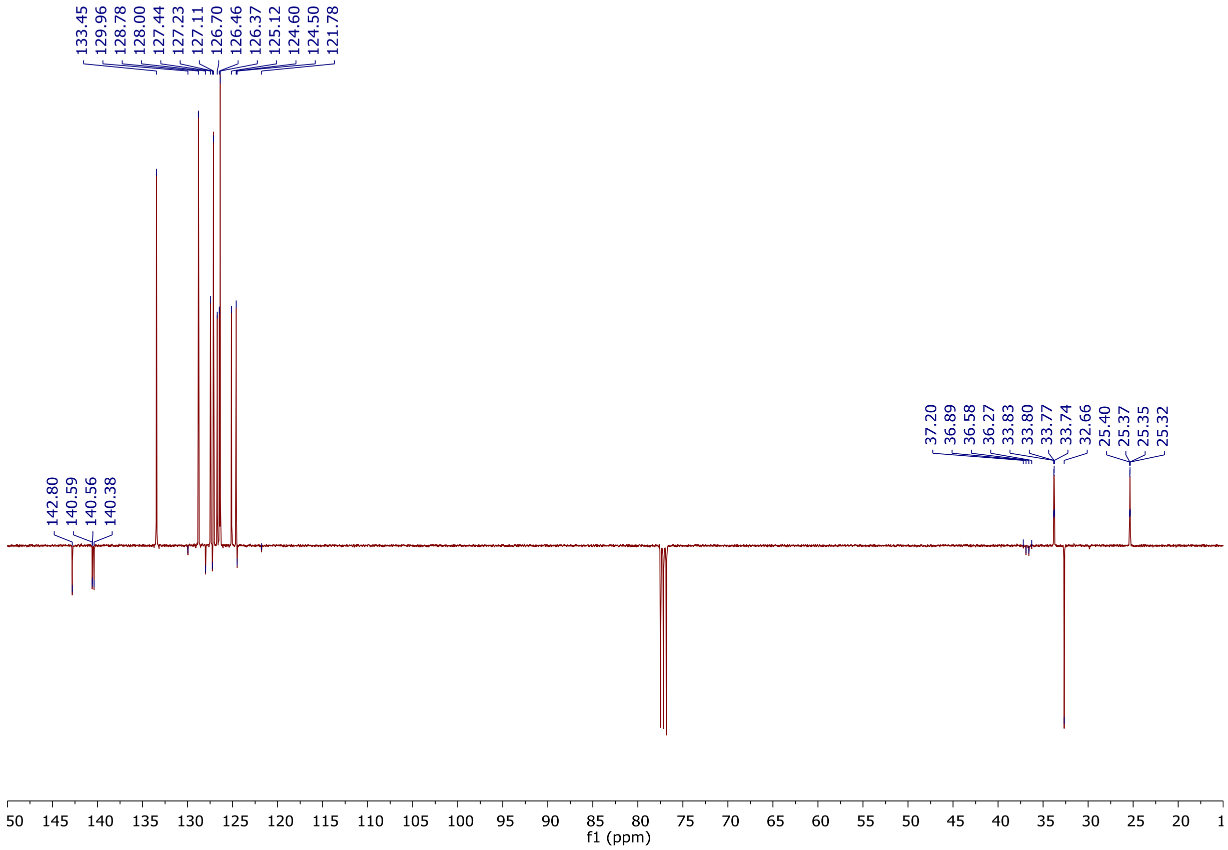

Figure S28. $^{13}C\{^{1}H\}_{jmod}$ NMR spectrum of (**7**) in $CDCl_3$.

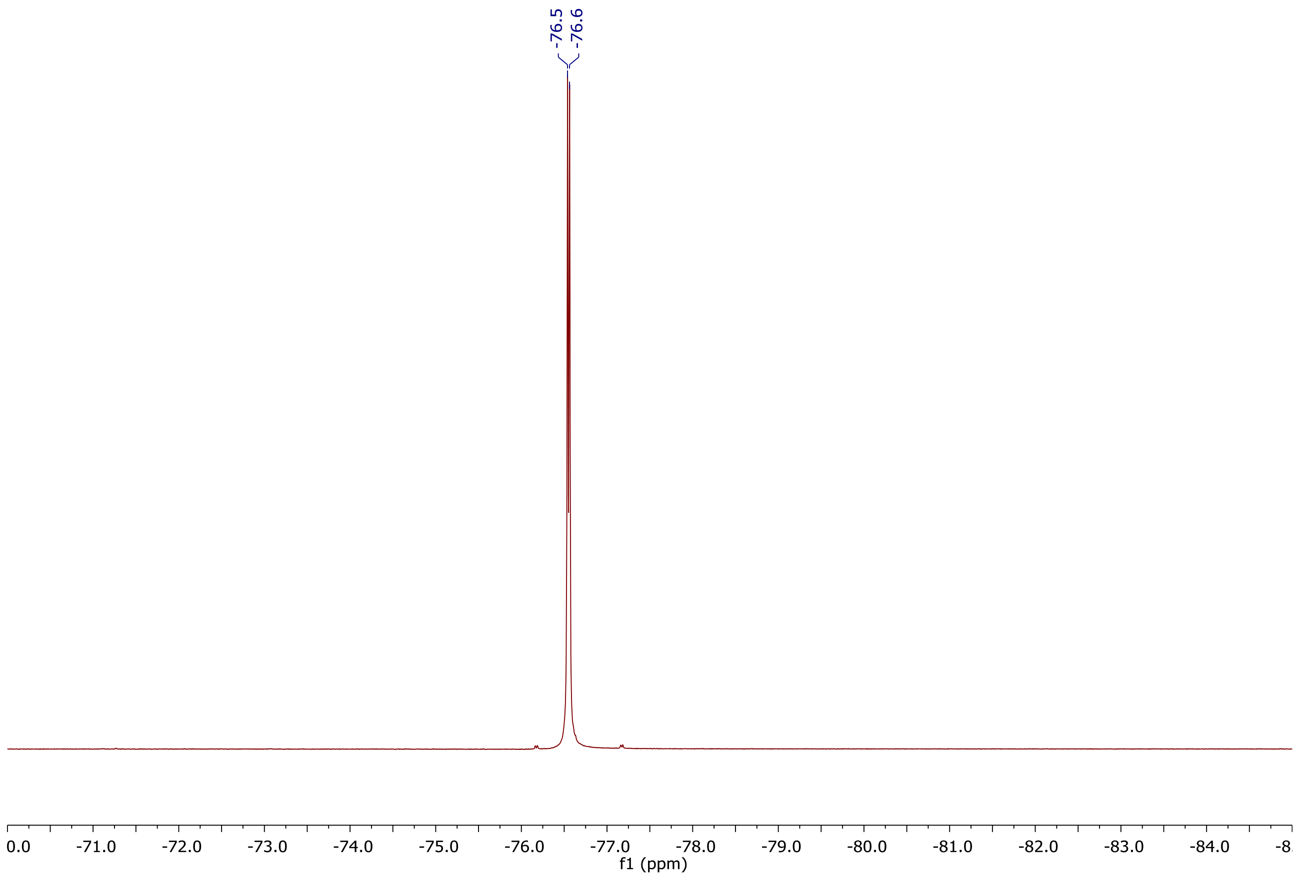

Figure S29. ^{19}F NMR spectrum of (**8**) in $CDCl_3$.

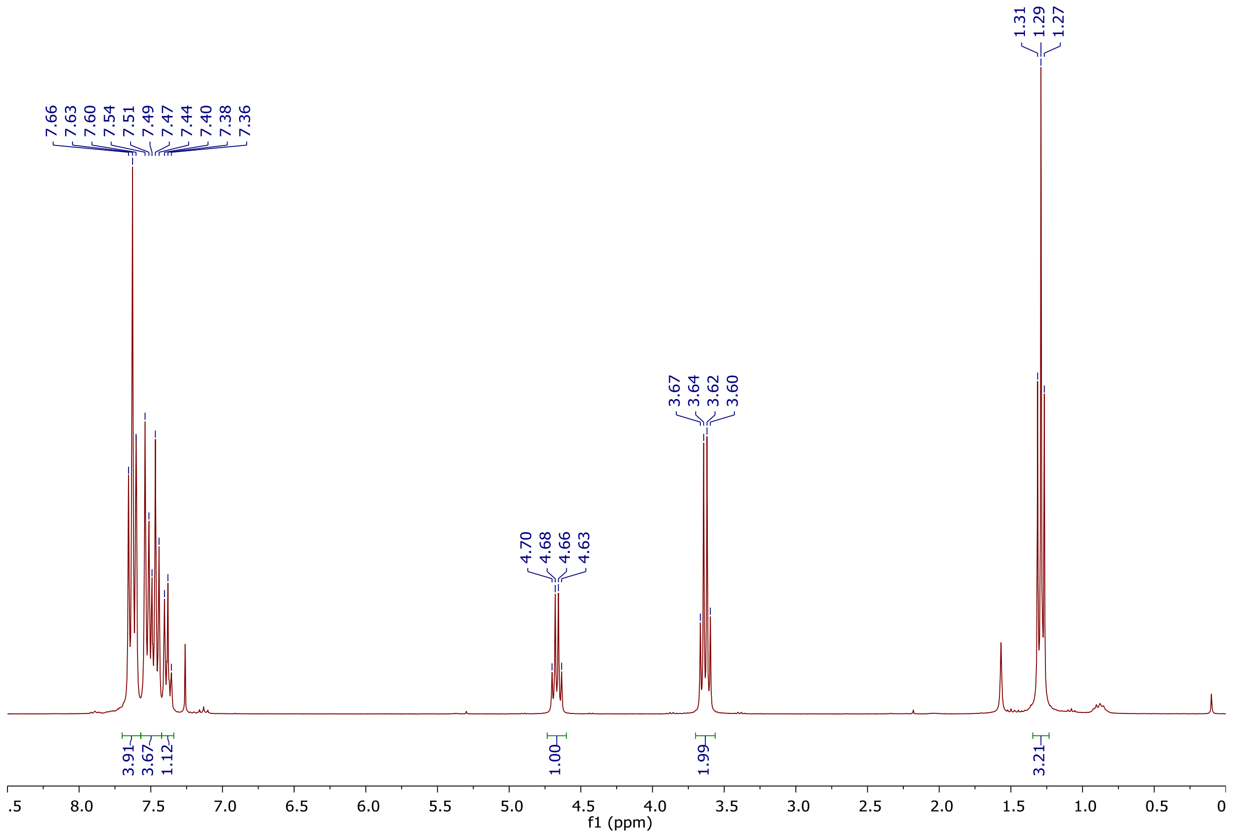

Figure S30. ^{1}H NMR spectrum of (**8**) in $CDCl_3$.

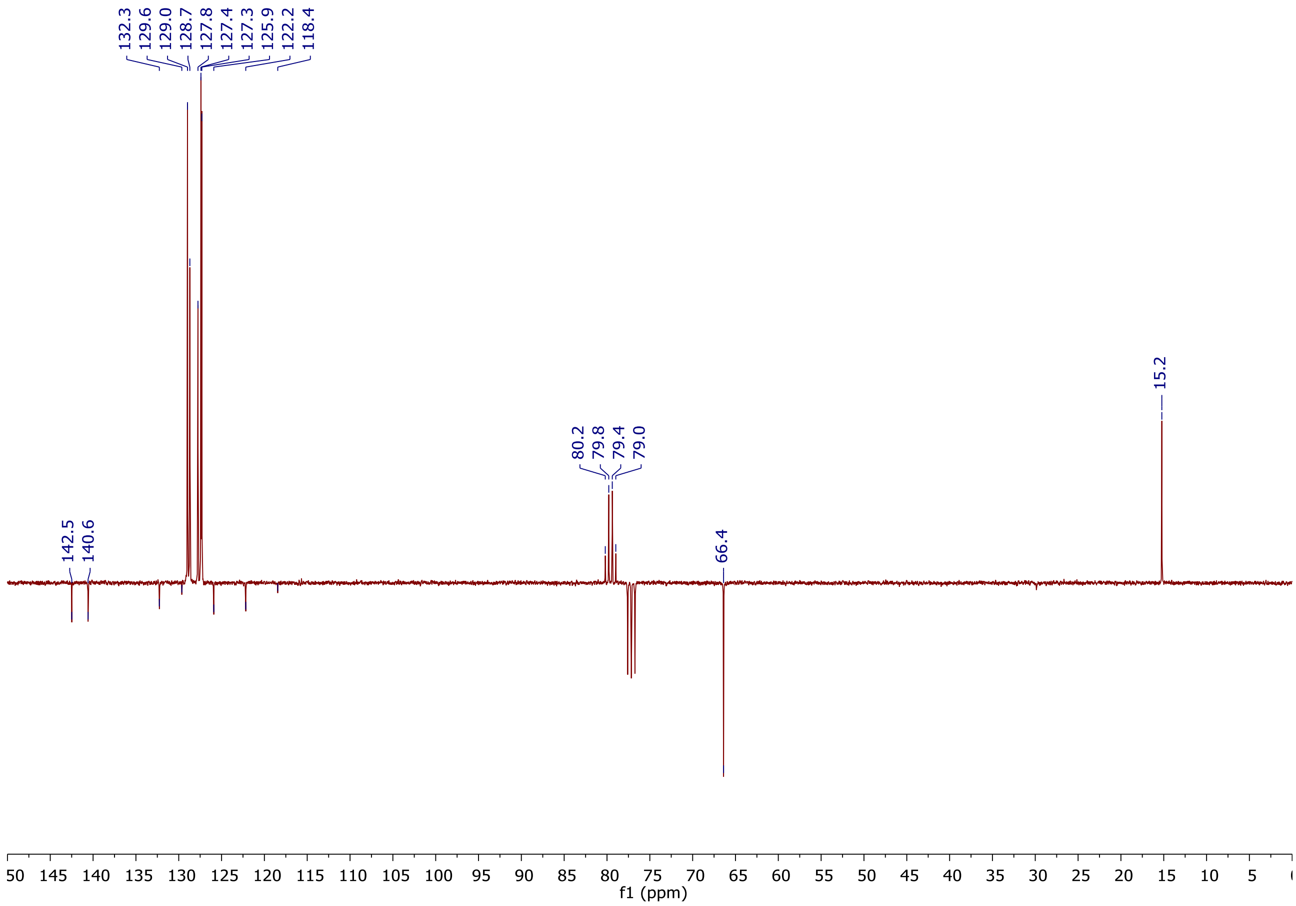

Figure S31. $^{13}C\{^{1}H\}_{jmod}$ NMR spectrum of (**8**) in $CDCl_3$.

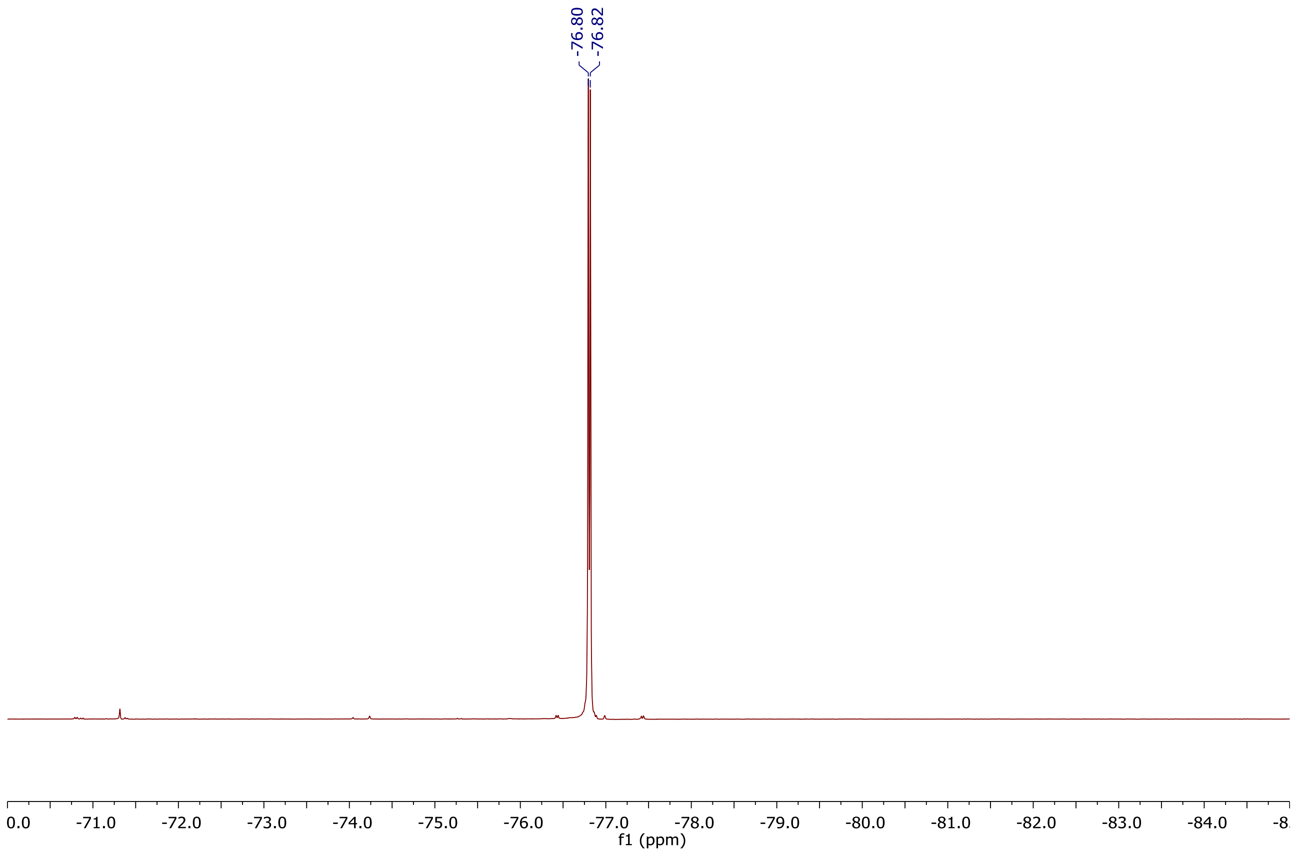

Figure S32. ^{19}F NMR spectrum of (**8’**) in $CDCl_3$ (crude reaction mixture).

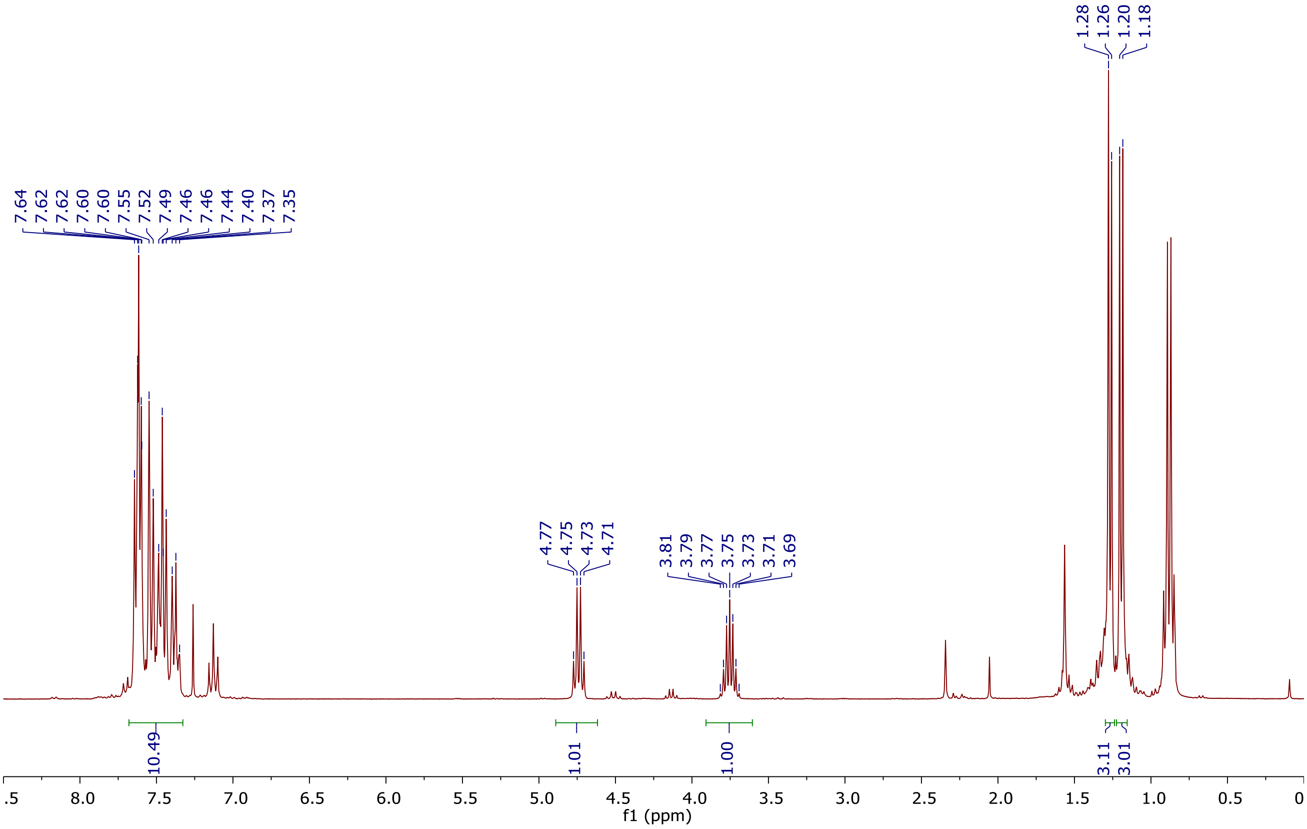

Figure S33. ^{1}H NMR spectrum of (**8'**) in $CDCl_3$ (crude reaction mixture).

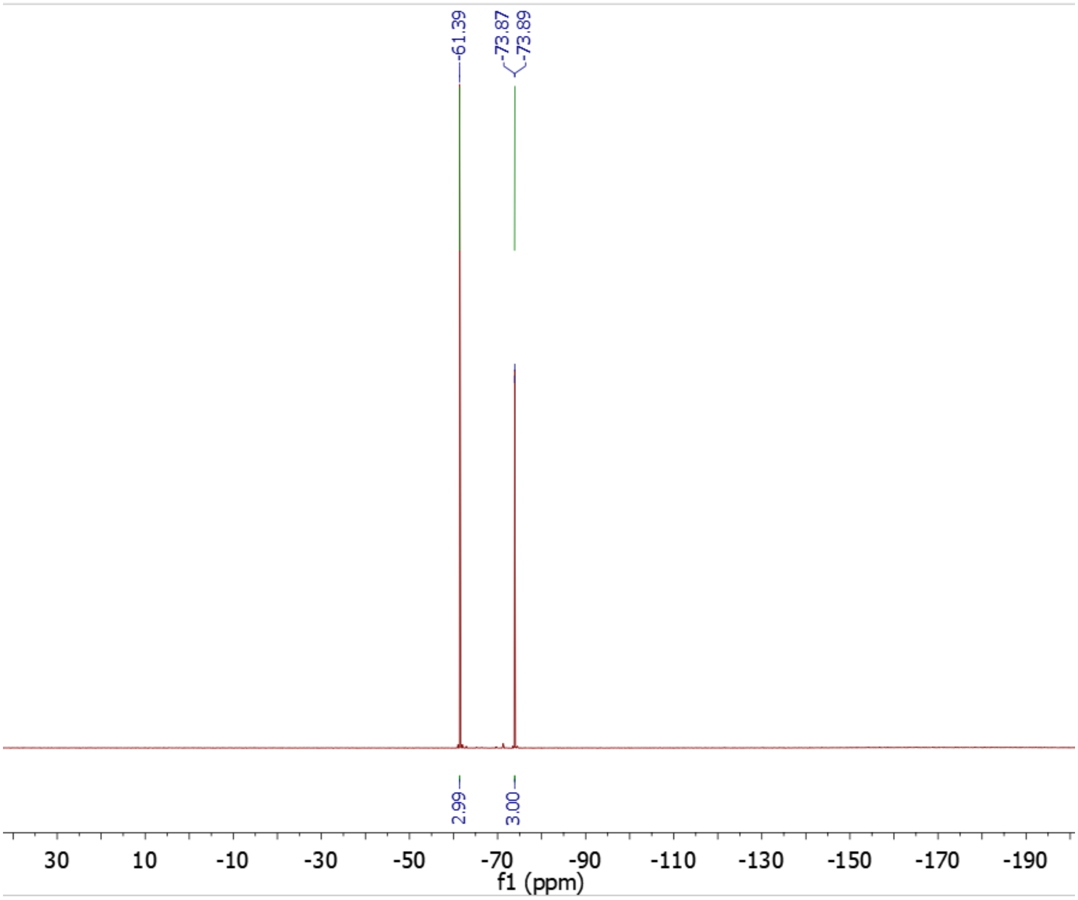

Figure S34. ^{19}F NMR spectrum of (**8"**) in $CDCl_3$.

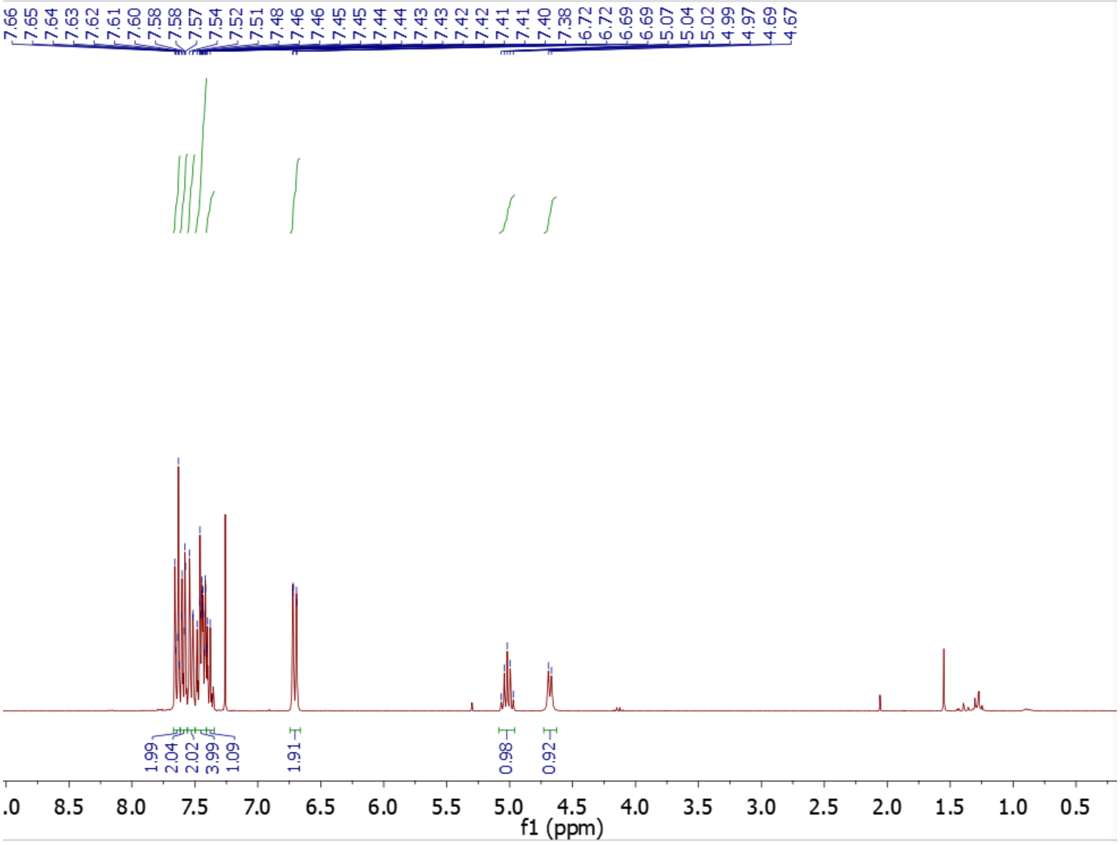

Figure S34. ^{1}H NMR spectrum of (**8”**) in $CDCl_3$.

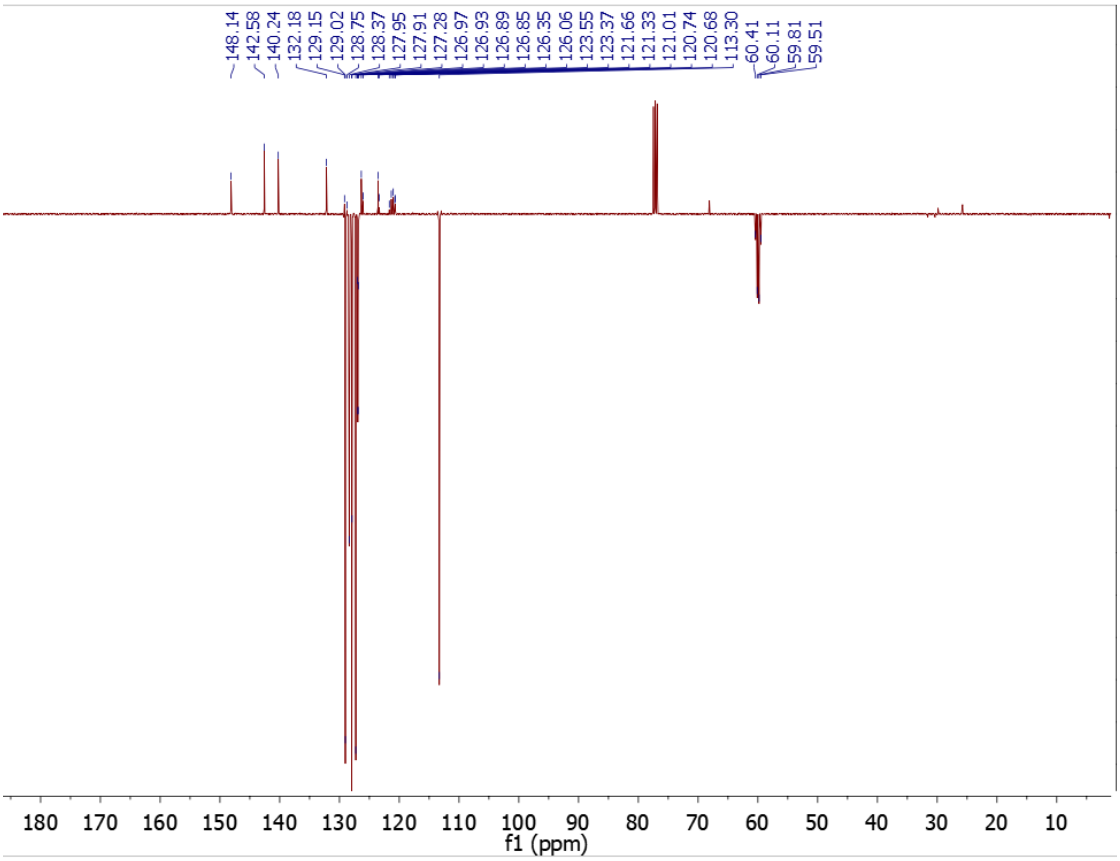

Figure S35. $^{13}C\{^{1}H\}_{jmod}$ NMR spectrum of (**8”**) in $CDCl_3$.

4. Crystallographic data

Crystallographic data were collected at low temperature (193(2) K) on a Bruker-AXS APEX II Quazar diffractometer equipped with a 30W air-cooled microfocus or on a Bruker-AXS PHOTON100 D8 VENTURE diffractometer, using MoK$_\alpha$ radiation (λ = 0.71073 Å). Phi- and omega-scans were used. An empirical absorption correction was performed with SADABS.[8] The structures were solved by direct intrinsic phasing method (SHELXT),[9] and refined using the least-squares method on F^2.[10] All H atoms on carbon atoms were refined isotropically at calculated positions using a riding model. CCDC 2124447 (**3-Ph**), 2124452 (**4'**) and 2124272 (**6**) contain the supplementary crystallographic data for this paper. These data can be obtained free of charge from The Cambridge Crystallographic Data Centre.

ID	(**Carbene 3-Ph**) MR634	(**DMAP adduct 4'**) MR706	(**cyclopropane 6**) MR705
formula	$2(C_{32}H_{55}AuB_{10}F_3N_4P_2)$, $2(C_2F_6NO_4S_2)$, CH_2Cl_2	$C_{39}H_{65}AuB_{10}F_3N_6P_2$, $C_2F_6NO_4S_2$	$C_{22}H_{17}F_3$
M_r	2484.85	1322.13	338.35
crystal system	Triclinic	Monoclinic	Orthorhombic
space group	*P-1*	*Cc*	$P2_12_12_1$
a (Å)	12.9747 (7)	11.5655 (7)	5.7542 (8)
b (Å)	19.5323 (11)	23.5016 (15)	16.803 (2)
c (Å)	20.5359 (11)	21.3348 (12)	17.010 (2)
α (°)	89.793 (2)	90	90
β (°)	87.456 (2)	97.358 (2)	90
γ (°)	80.079 (2)	90	90
V (Å^3)	5121.4 (5)	5751.2 (6)	1644.7 (4)
Z	2	4	4
ρ_{calc} (g cm^{-3})	1.611	1.527	1.366
μ (mm^{-1})	3.144	2.761	0.102
F(000)	2476	2656	704.0
crystal size (mm^3)	0.180 x 0.120 x 0.080	0.200 x 0.040 x 0.040	0.140 x 0.080 x 0.060
T/K	193 (2)	193 (2)	193 (2)
measd reflns	124878	108721	5064
Unique reflns (Rint)	17950 (0.0587)	22126 (0.0527)	3966 (0.0434)
reflns used for refinement	17950	22126	3966
refined parameters	1250	695	226
GOF on F^2	1.049	1.039	1.039
R_1[a] [I>2σ(I)]	0.0320	0.0327	0.0434
wR_2[b] [all data]	0.0766	0.0655	0.1300

[a] $R_1 = \Sigma||F_o| - |F_c||/ \Sigma |F_o|$. [b] $wR_2 = [\Sigma[w(F_o^2 - F_c^2)^2] / \Sigma [w(F_o^2)^2]]^{1/2}$

Table S1. Crystal Data, Data Collection, and Structure Refinement.

[8] Bruker, *SADABS*, Bruker AXS Inc., Madison, Wisconsin, USA.
[9] G. M. Sheldrick *Acta Cryst*. **2015**, A71, 3–8.
[10] G. M. Sheldrick *Acta Cryst*. **2015**, C71, 3–8.

	Unit 1	Unit 2
Au-C	1.971(2)	1.971(4)
P-Au	2.347(1)/2.348(1)	2.361(1)/2.355(1)
C-C_{ipso}	1.444(6)	1.439(6)
C-CF_3	1.500(6)	1.496(6)
PAuC	135.7(1)/133.7(1)	134.5(1)/135.9(1)
PAuP	90.59(4)	89.58(4)
C_{ortho}-C_{ipso}-C-Au	0.6(5)	-3.4(6)

Table S2. Key geometric features of the two independent molecules of the crystal structure of **3-Ph.**

5. Computational details

All calculations were performed using the Gaussian 09 package[11] and the B3PW91 hybrid functional[12] on the real experimental systems. The gold atom was described with the relativistic electron core potential SDD and associated basis set,[13] augmented by a set of f-orbital polarization functions.[14] The 6-31G** basis set was employed for all other atoms.[15] Frequency calculations were undertaken to confirm the nature of the stationary points, yielding zero imaginary frequency for *minima* and one imaginary frequency for transition states (TS), corresponding to the expected process (rotation of phenyl ring). The connectivity of the transition states and their adjacent *minima* was confirmed by intrinsic reaction coordinate (IRC)[16] calculations. All the geometrical structures were plotted with Chemcraft program.[17] For the frontier orbitals, the atomic orbital compositions of each MO (%) have been computed thanks to Multiwfn 3.6 package.[18]

The bonding situation in all systems was studied using Natural Bond Orbital[19] analyses (NBO, 7.0 version).[20] Charge transfer between the carbene and the metallic fragment has been calculated using atomic NPA charges. The Natural Localized Molecular Orbitals (NLMO) associated to the interactions involving the vacant of the carbene, *i.e.* the $d_{xz}(Au)\rightarrow 2p^{\pi}(C_{carbene})$ and $\pi_{C=CAr}\rightarrow 2p^{\pi}(C_{carbene})$ interactions, have been analyzed. NLMO plots associated to the $Au\rightarrow C_{carbene}$ back-donation or $Aryl\rightarrow C_{carbene}$ interaction were drawn (cutoff : 0.04) with Chemcraft program.[17]

[11] Gaussian 09, M. J. Frisch, G. W. Trucks, H. B. Schlegel, G. E. Scuseria, M. A. Robb, J. R. Cheeseman, G. Scalmani, V. Barone, B. Mennucci, G. A. Petersson, H. Nakatsuji, M. Caricato, X. Li, H. P. Hratchian, A. F. Izmaylov, J. Bloino, G. Zheng, J. L. Sonnenberg, M. Hada, M. Ehara, K. Toyota, R. Fukuda, J. Hasegawa, M. Ishida, T. Nakajima, Y. Honda, O. Kitao, H. Nakai, T. Vreven, J. A. Montgomery, Jr., J. E. Peralta, F. Ogliaro, M. Bearpark, J. J. Heyd, E. Brothers, K. N. Kudin, V. N. Staroverov, T. Keith, R. Kobayashi, J. Normand, K. Raghavachari, A. Rendell, J. C. Burant, S. S. Iyengar, J. Tomasi, M. Cossi, N. Rega, J. M. Millam, M. Klene, J. E. Knox, J. B. Cross, V. Bakken, C. Adamo, J. Jaramillo, R. Gomperts, R. E. Stratmann, O. Yazyev, A. J. Austin, R. Cammi, C. Pomelli, J. W. Ochterski, R. L. Martin, K. Morokuma, V. G. Zakrzewski, G. A. Voth, P. Salvador, J. J. Dannenberg, S. Dapprich, A. D. Daniels, O. Farkas, J. B. Foresman, J. V. Ortiz, J. Cioslowski, and D. J. Fox, Gaussian, Inc., Wallingford CT, **2009**.

[12] a) A. D. Becke, *Phys. Rev.* **1988**, *A38*, 3098–3100; b) A. D. Becke *J. Chem. Phys.* **1993**, *98*, 5648–5652; b) J. P. Perdew, in *Electronic Structure of Solids '91*, Ed. P. Ziesche and H. Eschrig, Akademie Verlag, Berlin, **1991**, 11.

[13] a) D. Andrae, U. Häussermann, M. Dolg, H. Stoll, H. Preuss, *Theor. Chim. Acta* **1990**, *77*, 123–141; b) M. Dolg, *Modern Methods and Algorithm of Quantum Chemistry, Vol. 1* (Ed.: J. Grotendorst), John von Neuman Institute for Computing, Jülich (Germany), **2000**, 479.

[14] A. W. Ehlers, M. Biihme, S. Dapprich, A. Gobbi, A. Hijllwarth, V. Jonas, K. F. Kiihler, R. Stegmann, A. Veldkamp, G. Frenking, *Chem. Phys. Letters* **1993**, *208*, 111–114.

[15] P. C. Hariharan, J. A. Pople, *Theor. Chim. Acta* **1973**, *28*, 213–222.

[16] a) K. Fukui, *Acc. Chem. Res.*, **1981**, *14*, 363–368; b) H. P. Hratchian, H. B. Schlegel, in *Theory and Applications of Computational Chemistry: The First 40 Years*, Ed. C. E. Dykstra, G. Frenking, K. S. Kim, G. Scuseria, Elsevier, Amsterdam, **2005**, 195.

[17] Chemcraft graphical software for visualization of quantum chemistry computations. https://www.chemcraftprog.com

[18] T. Lu, F. Chen, *J. Comput. Chem.*, **2012**, *33*, 580–592.

[19] a) E. Reed, L. A. Curtiss, F. Weinhold, *Chem. Rev.* **1988**, *88*, 899–926; b) J. P. Foster, F. Weinhold, *J. Am. Chem. Soc.* **1980**, *102*, 7211–7218; c) A. E. Reed, F. Weinhold, *J. Chem. Phys.* **1985**, *83*, 1736–1740.

[20] NBO 7.0 program, E. D. Glendening, J. K. Badenhoop, A. E. Reed, J. E. Carpenter, J. A. Bohmann, C. M. Morales, P. Karafiloglou, C. R. Landis, and F. Weinhold, Theoretical Chemistry Institute, University of Wisconsin, Madison, **2018**.

For each system, a charge decomposition analysis (CDA) was carried out with the CDA 2.2 program developed by G. Frenking.[21] The orbital contributions to the charge distributions are divided into four parts: *(i)* the mixing of the occupied orbitals of the ligand (carbene) and the unoccupied MOs of the metal fragment (P,P)Au^+ (Ligand→Au donation *d*), *(ii)* the mixing of the unoccupied orbitals of the ligand and the occupied MOs of the metal fragment (Ligand←Au back-donation *b*), *(iii)* the mixing of the occupied orbitals of the ligand and the occupied orbitals of the metal fragment (Ligand↔Au repulsive polarization *r*), and *(iv)* the mixing of the unoccupied orbitals of the ligand and the unoccupied orbitals of the metal fragment (residual term Δ).

The absorption spectrum was calculated at SMD(DCM)/B3PW91/SDD+f(Au), 6-31G**(other atoms)// B3PW91/SDD+f(Au), 6-31G**(other atoms) level by using time-dependent density functional theory (TD-DFT)[22] method on the geometry of the ground-state. Solvents effects (DCM: dichloromethane) was included by means of the universal Solvation Model based on Density (SMD).[23]

For the NMR calculations of the carbene gold complexes, the $^{13}C_{carbene}$ NMR chemical shift (δ in ppm) and J_{PC} and J_{PF} coupling constants (in Hertz) were computed by employing the direct implementation of the Gauge Including Atomic Orbitals (GIAO),[24] with the IGLOII[25] basis set on B, C, H, N, F and P atoms and using as reference $SiMe_4$, optimized at the same level of theory.

[21] S. Dapprich, G. Frenking, *J. Phys. Chem.* **1995**, *99*, 9352–9362.

[22] a) R. Bauernschmitt, R. Ahlrichs, Chem. Phys. Lett. **1996**, *2*56, 454–464; b) M. E. Casida, C. Jamorski, K. C. Casida, D. R. Salahub, *J. Chem. Phys.* **1998**, *108*, 4439–4449; c) R.E. Stratmann, G.E.Scuseria, M. J. Frisch, *J. Chem. Phys.* **1998**, *109*, 8218–8224.

[23] Marenich, A. V.; Cramer, C. J.; Truhlar, D. G. *J. Phys. Chem. B* **2009**, *113*, 6378–6396.

[24] a) F. London, *J. Phys. Radium* **1937**, *8*, 397–409; b) R. McWeeny, *Phys. Rev.* **1962**, *126*, 1028–1034; c) R. Ditchfield, *Mol. Phys.* **1974**, *27*, 789–807; d) K. Wolinski, J. F. Hilton, P. Pulay, *J. Am. Chem. Soc.* **1990**, *112*, 8251–8260; e) J. R. Cheeseman, G. W. Trucks, T. A. Keith, M. J. Frisch, *J. Chem. Phys.* **1996**, *104*, 5497–5509.

[25] W. Kutzelnigg, U. Fleischer, M. Schindler, *The IGLO-Method: Ab Initio Calculation and Interpretation of NMR Chemical Shifts and Magnetic Susceptibilities*, Springer-Verlag, Heidelberg, **1990**, vol. 23.

6. Computational results

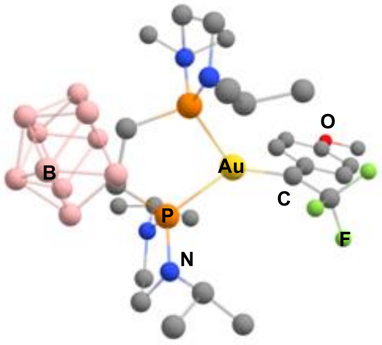

3-OMe

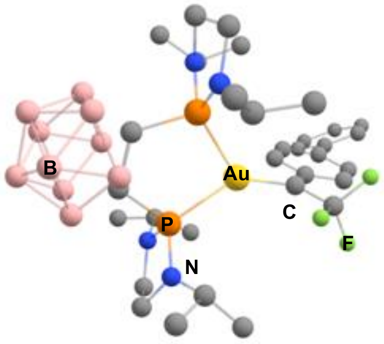

3-Ph

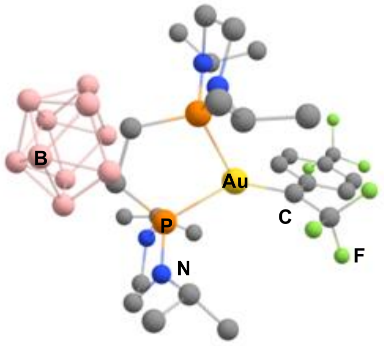

3-CF$_3$

	**3-OMe**	**3-Ph**		**3-CF$_3$**
	**Geometrical parameters**			
	**DFT**	**DFT**	**X-Ray**	**DFT**
**Au-C**	1.987	1.983	1.971(2)	1.971
**P-Au**	2.420/2.422	2.418/2.418	2.347(1)/2.348(1)	2.408/2.412
**C-C^{ipso}**	1.423	1.429	1.444(6)	1.442
**C-C$_{CF3}$**	1.515	1.515	1.500(6)	1.514
**PAuC**	133.49/136.62	135.91/134.20	135.7(1)/133.7(1)	134.45/134.74
**PAuP**	89.81	89.88	90.59(4)	89.79
**Au-C-C$_{CF3}$**	114.57	114.76	116.2(3)	114.97
**Au-C-C$_{Aryl}$**	128.56	128.42	127.4(3)	128.31
**C$_{Aryl}$-C-C$_{CF3}$**	116.85	116.82	116.4(4)	116.71
**C^{ortho}-C^{ipso}-C-Au**	3.8	-0.9	0.6(5)	-1.8
	**NMR Data**			
	**DFT^{a}**			
**δ ^{13}C (C$_{carb}$, ppm)**	271.4	276.9		284.2
**$^{2}J_{PC}$ (Hz)**	92.5	100.4		135.3
**$^{4}J_{PF}$ (Hz)**	16.9	21.2		39.1
**$^{8}J_{PF}$ (Hz)**				14.7
	**Experimental**			
**δ ^{13}C (C$_{carb}$, ppm)**	268.5	270.9		265.8
**$^{2}J_{PC}$ (Hz)**	79.5	94.4		109.0
**$^{4}J_{PF}$ (Hz)**	14.0	23.2		32.1
**$^{8}J_{PF}$ (Hz)**				10.8

Table S3. Main geometrical parameters (distances in Å and bond and dihedral angles in °) and NMR data ($^{13}C_{carbene}$ chemical shifts δ in ppm and J_{PC}, J_{PF} coupling constants in Hz) for the complexes **3-OMe**, **3-Ph** and **3-CF$_3$** calculated at the B3PW91/SDD+f(Au), 6-31G**(other atoms) level of theory. ^{a} NMR calculations carried out at GIAO-B3PW91/IGLO(II) for H, B, C, N, F, P atoms and SDD+f for Au.

	3-OMe	3-Ph	$3\text{-}CF_3$
	**NBO Analysis**		
**WBI (Au-C)**<br>**WBI (C-C^{ipso})**	0.677<br>1.312	0.687<br>1.289	0.710<br>1.234
**CT**	-0.10	-0.14	-0.25
**d_{xz}(Au) → $2p^{\pi}$(C) interaction**<br><br>**NLMO d_{xz}(Au)**	87.8 % Au<br>7.3 % $C_{Carbene}$<br>1.0 % C_{Ar}<br>1.0 % C_{Ar}	86.3 % Au<br>9.7 % $C_{Carbene}$<br>1.7 % C_{Ar}<br>0.4 % C_{Ar}	82.5 % Au<br>15.5 % $C_{Carbene}$<br>0.4 % C_{Ar}<br>0.2 % C_{Ar}
**$\pi_{C=CAr}$ → $2p^{\pi}$(C) interaction**<br><br>**NLMO $\pi_{C=CAr}$**	24.2 % $C_{carbene}$<br>49.3 % C_{Ar}<br>8.5 % C_{Ar}<br>7.7 % C_{Ar}	16.3 % $C_{carbene}$<br>45.1 % C_{Ar}<br>27.2 % C_{Ar}	10.1 % $C_{carbene}$<br>45.8% C_{Ar}<br>29.2 % C_{Ar}
	**CDA Analysis**		
**CR_2 → AuL_2 donation (d)**	0.414	0.409	0.394
**Au → CR_2 back-donation (b)**	0.191	0.195	0.211
**d/b ratio**	2.16	2.09	1.86
**Au ↔ C repulsion**	-0.379	-0.396	-0.397
**Residue term (Δ)**	-0.017	-0.015	-0.012

Table S4. Bonding situation for complexes **3-OMe**, **3-Ph**, and **$3\text{-}CF_3$** calculated at the B3PW91/SDD+f(Au),6-31G**(other atoms) level of theory. Wiberg Bond indexes (WBI) for Au-$C_{carbene}$ and $C_{carbene}$-C_{ipso}. Charge transfer (CT) from carbene to gold fragment accounting for NBO calculations. Contributions of the main atoms (in percent) in the NLMO associated to d_{xz}(Au) and $\pi_{C=CAr}$ orbitals. CDA analysis for the different complexes.

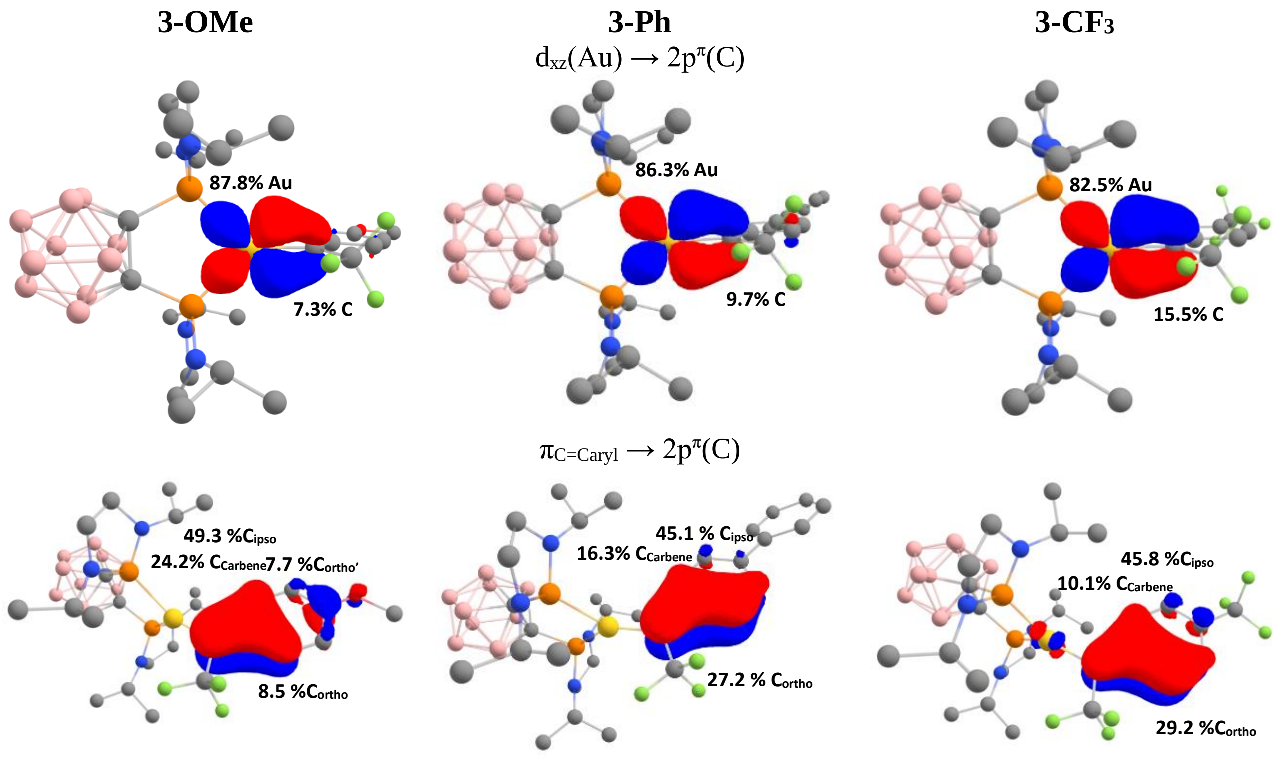

Figure S36. Plots of the NLMO (cutoff : 0.04) associated to the back-donation Au→$C_{carbene}$ (d_{xz}(Au) → $2p^{\pi}$(C)) and the delocalization of the aryl group on the vacant of the carbene ($\pi_{C=CAr}$ → $2p^{\pi}$(C)) for complexes **3-OMe**, **3-Ph** and **$3\text{-}CF_3$**. Contribution of gold and $C_{carbene}$ atoms in percent.

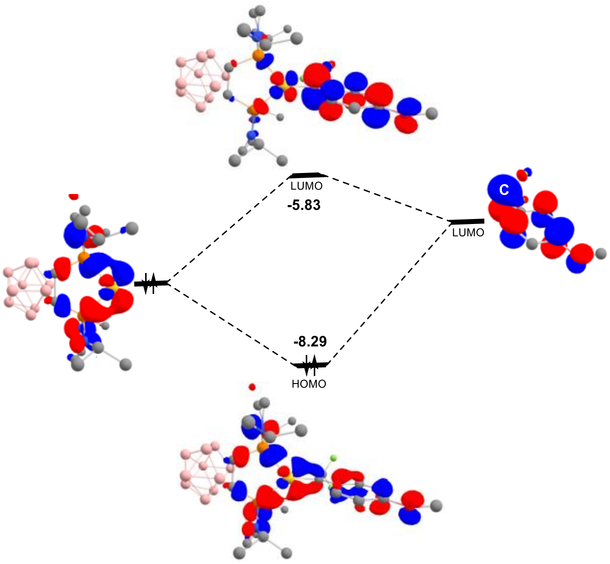

3-OMe

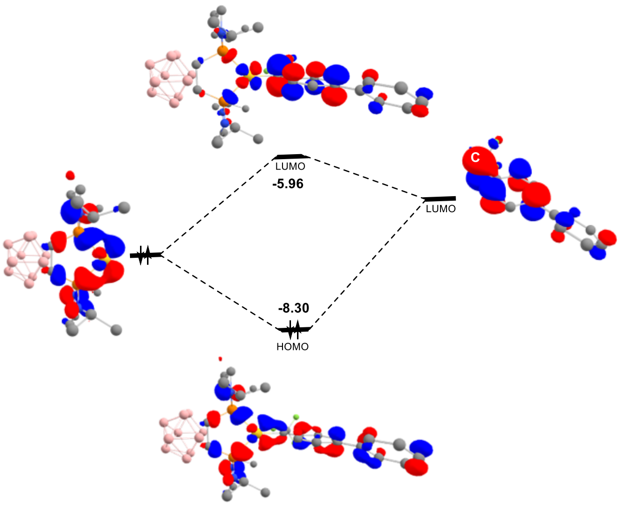

3-Ph

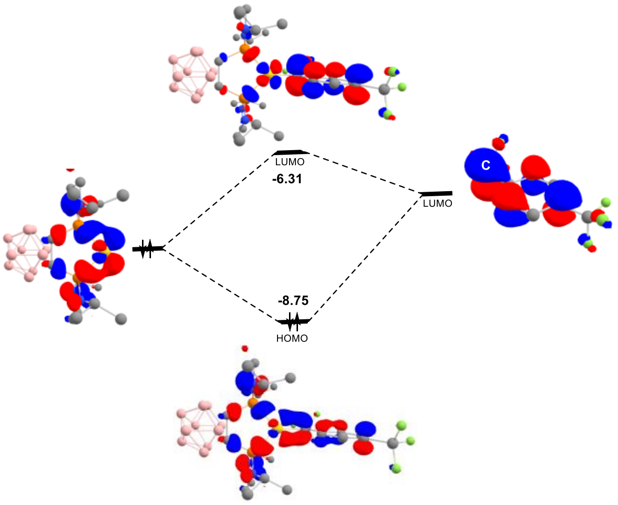

3-CF$_3$

Figure S37. Molecular diagrams corresponding to interaction between d_{xz}(Au) and $2p^{\pi}$(C) orbitals for each complex **3-OMe**, **3-Ph** and **3-CF$_3$**. Plot of the HOMO and LUMO and their energy levels for the α-CF$_3$ gold carbene complexes.

For all complexes, the plot of the frontier orbitals evidences interaction of the $2p^{\pi}$(C) orbital with both the metal and the aryl substituent. The energy gap between the frontier orbitals is relatively small in all cases: 2.34 eV for **3-Ph**, 2.46 eV for **3-OMe** and 2.44 eV for **3-CF$_3$**.

Excited state	λ (nm)	Transition energy (eV)	f	Electronic transitions[a]
1	634.8	1.95	1.13	HOMO→LUMO
2	517.0	2.40	0.01	HOMO -1 → LUMO
3	504.2	2.46	0.01	HOMO -2 → LUMO
4	463.7	2.67	0.03	HOMO -4 → LUMO (59.8 %)<br>HOMO -6 → LUMO (40.2%)
5	406.6	3.05	0.26	HOMO -3 →LUMO (86.7%)<br>HOMO -6 → LUMO (13.3%)
6	399.89	3.10	0.08	HOMO -6 → LUMO (51.7 %)<br>HOMO -4 → LUMO (35.8 %)<br>HOMO -3 → LUMO (12.5 %)
7	383.78	3.23	0.00	HOMO -5 → LUMO
8	367.43	3.37	0.00	HOMO → LUMO +1
9	363.95	3.41	0.03	HOMO -7 → LUMO

Table S5. TD-DFT calculations for complex **3-Ph**: absorption wavelengths λ (in nm) and transition energies (in eV) corresponding to the main $\pi \rightarrow \pi^*$ absorptions for the 9 first excited states, oscillator strength f, associated electronic transitions. [a]With HOMO-1 and HOMO-2 centered on the P^P-ligand; HOMO-3, HOMO-4, HOMO -5 and HOMO-6 centered on the biphenyl group; HOMO-7 and LUMO+1 centered on Au-C and Au-P bonds.

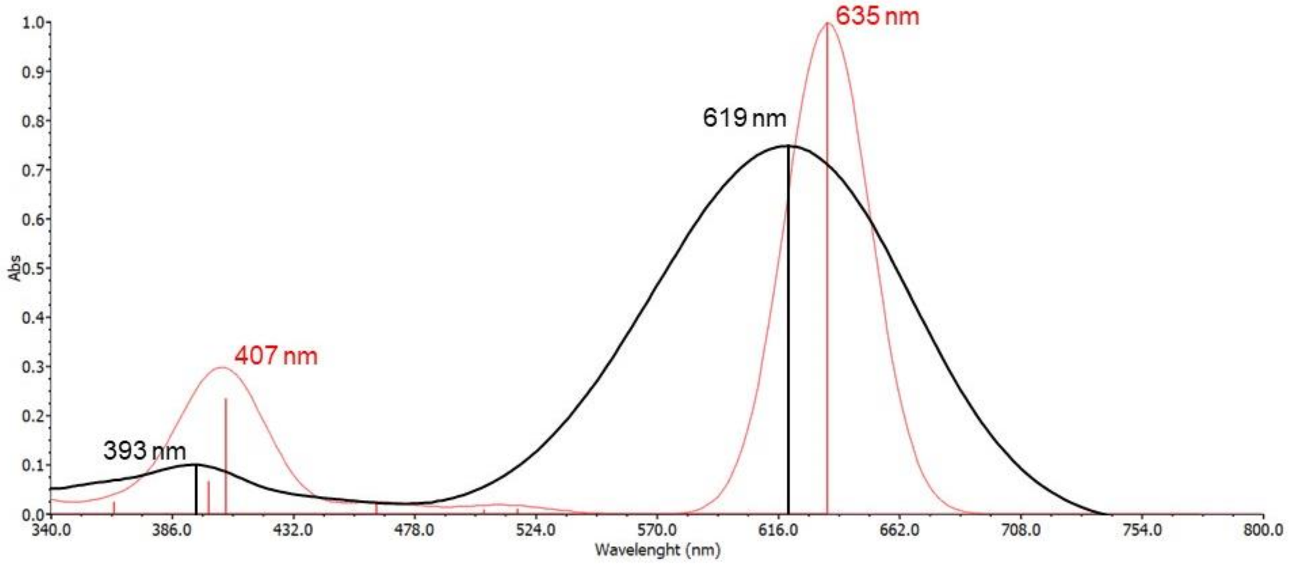

Figure S38. Experimental (black) and TD-DFT simulated (red) UV-vis spectrum of **3-Ph**.

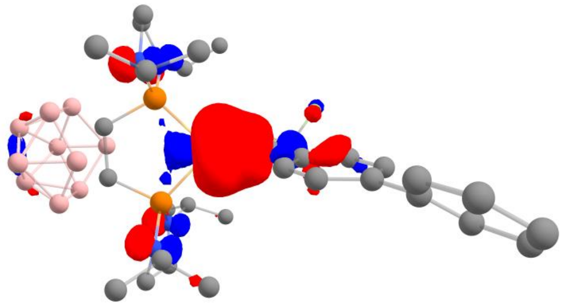	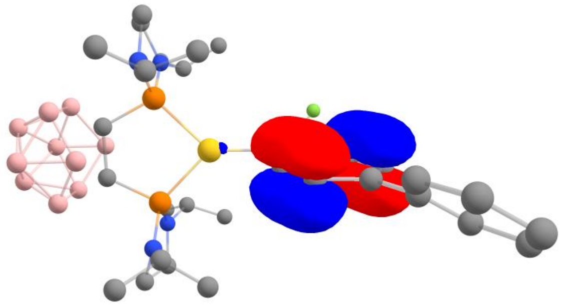
HOMO-7	HOMO-6
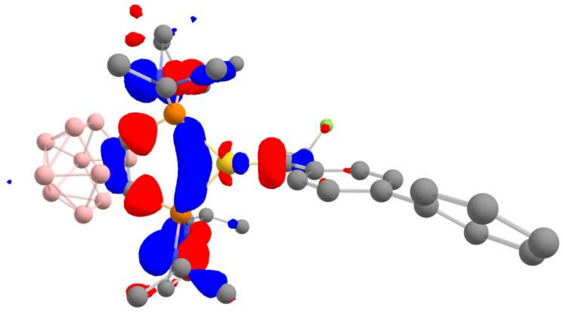	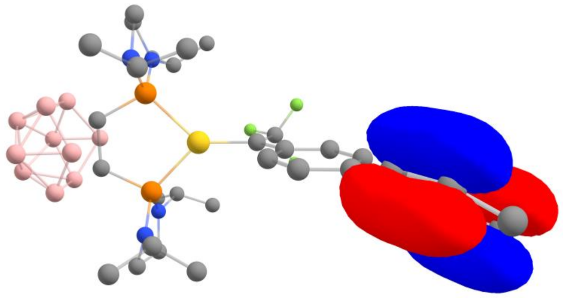
HOMO-5	HOMO-4
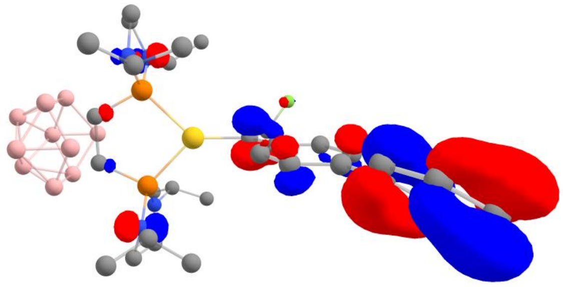	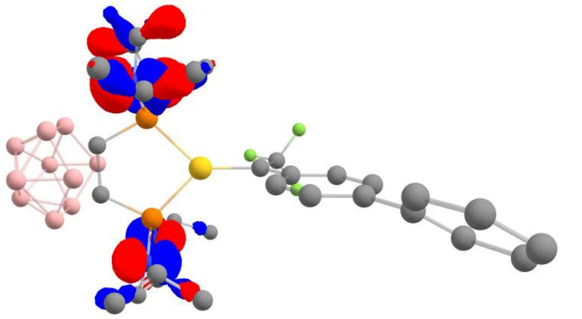
HOMO-3	HOMO-2
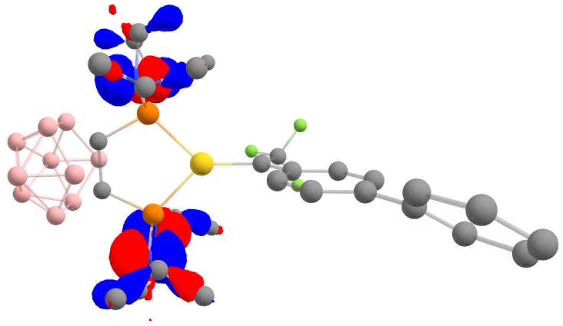	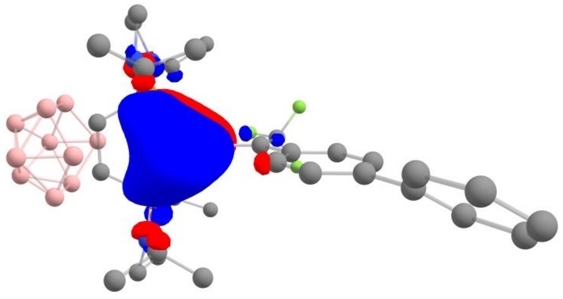
HOMO-1	LUMO+1

Figure S40. Plots of the molecular orbitals (cutoff : 0.04) corresponding to the electronic transitions described in Table S5.

	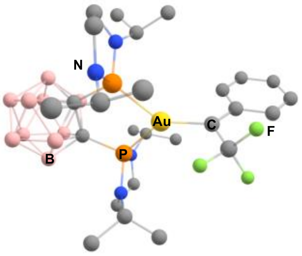	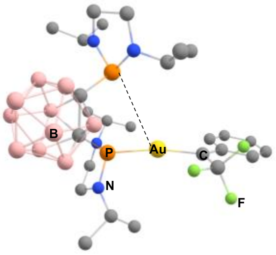
	**3-H**	**3-H$_{dissoc\ P}$**
**ΔG**	0.0	16.6
	**Geometrical parameters**	
**Au-C**	1.975	2.020
**P-Au**	2.412/2.415	2.343 /3.496
**C-C^{ipso}**	1.438	1.412
**C-CF$_3$**	1.515	1.526
**PAuC**	136.41/133.86	179.99/108.16
**PAuP**	89.73	71.84
**P-Au-C-C^{ipso}**	-90.1	-162.2
**C^{ipso}-C^{ortho}-C-Au**	3.3	2.2
**WBI (Au-C)**<br>**WBI (C-C$_{Ar}$)**	0.705<br>1.251	0.623<br>1.383
**CT**	-0.20	0.19
**d$_{xz}$(Au) → 2p$^{\pi}$(C) interaction**<br>**NLMO d$_{xz}$(Au)**	83.2 % Au<br>14.8 % C$_{Carbene}$<br>0.3 % C$_{Ar}$<br>0.2 % C$_{Ar}$	92.8 % Au<br>4.4 % C$_{Carbene}$<br>0.4 % C$_{Ar}$<br>0.4 % C$_{Ar}$
**π$_{C=CAr}$ → 2p$^{\pi}$(C) interaction**<br>**NLMO π$_{C=CAr}$**	10.6 % C$_{Carbene}$<br>45.0 % C$_{Ar}$<br>30.2 % C$_{Ar}$	18.9 % C$_{carbene}$<br>45.8 % C$_{Ar}$<br>25.4 % C$_{Ar}$
	**CDA Analysis**	
**CR$_2$ → AuL$_2$ donation (d)**	0.402	0.378
**Au → CR$_2$ back-donation (b)**	0.207	0.105
**d/b ratio**	1.94	3.60
**Au ↔ C repulsion**	-0.393	-0.348
**Residue term (Δ)**	-0.014	-0.015

Table S6. Main geometrical parameters and bonding situation (NBO and CDA analyses) for the complexes **3-H** and **3-H$_{dissoc\ P}$** (dissociation of one phosphine arm) calculated at the B3PW91/SDD+f(Au),6-31G**(other atoms) level of theory. Distances are given in angstroms, bond angles and bond dihedral angles in degrees. Charge transfer (CT) from carbene to gold fragment accounting for NBO calculation. Contributions of the main atoms (in percent) in the NLMO associated to d$_{xz}$(Au) and π$_{C=C}$**Ar**.

	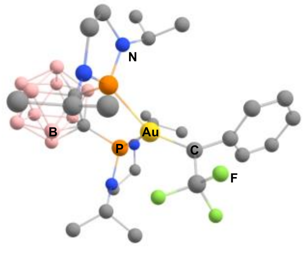	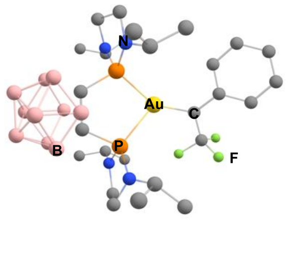	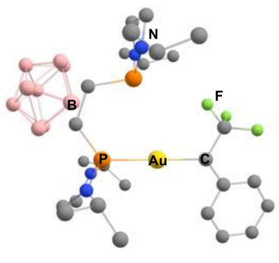
	**$TS_{rot\ Ph}$**	**$TS_{rot\ AuC}$**	**$TS_{rot\ AuC\text{-}dissoc\ P}$**[a]
**$\Delta G^{\neq}$**	$\Delta G^{\neq}$ 9.6	$\Delta G^{\neq}$ 20.5	$\Delta G^{\neq}$ 23.5
**Geometrical parameters**			
**Au-C**	1.934	2.023	2.024
**P-Au**	2.391/2.399	2.407/2.579	2.363/3.600
**C-C^{ipso}**	1.476	1.427	1.413
**C-CF_3**	1.511	1.512	1.520
**PAuC**	135.30/135.01	151.98/116.62	179.99
**PAuP**	89.56	88.47	75.25
**P-Au-C-C^{ipso}**	-90.1	16.3	-78.99
**C^{ipso}-C^{ortho}-C-Au**	90.9	-35.4	-14.9
**NBO Analysis**			
**WBI (Au-C)**<br>**WBI (C-C_{Ar})**	0.786<br>1.056	0.661<br>1.147	0.632<br>1.391
**CT**	-0.34	-0.05	0.20
**d_{xz}(Au) → $2p^{\pi}$(C) interaction**<br>**NLMO d_{xz}(Au)**	81.0 % Au<br>17.6 % $C_{Carbene}$<br>0.07 % C_{Ar}<br>0.07 % C_{Ar}	89.1 % Au<br>7.5 % $C_{Carbene}$<br>0.3 % C_{Ar}<br>1.5 % C_{Ar}	92.9 % Au<br>4.2 % $C_{Carbene}$<br>0.4 % C_{Ar}<br>0.4 % C_{Ar}
**$\pi_{C=CAr}$ → $2p^{\pi}$(C) interaction**<br>**NLMO $\pi_{C=CAr}$**	0.4% $C_{Carbene}$<br>42.6 % C_{Ar}<br>40.9 % C_{Ar}	17.5 % $C_{Carbene}$<br>44.7 % C_{Ar}<br>27.4 % C_{Ar}	19.2 % $C_{carbene}$<br>45.7 % C_{Ar}<br>25.3 % C_{Ar}
**CDA Analysis**			
**CR_2 → AuL_2 donation (d)**	0.386	0.457	0.387
**Au → CR_2 back-donation (b)**	0.259	0.152	0.097
**d/b ratio**	1.49	3.01	3.99
**Au ↔ C repulsion**	-0.392	-0.335	-0.346
**Residue term (Δ)**	-0.007	-0.006	0.007

[a] another TS for the rotation about the Au–C bond with dissociation of one P arm was also found on the PES.

Table S7. Main geometrical parameters and bonding situation (NBO and CDA analyses) for the transition states associated to the rotation of the phenyl ring (**$TS_{rot\ Ph}$**), the rotation of the Au=C bond with the 2 phosphines coordinated at gold (**$TS_{rot\ AuC}$**) or only 1 phosphine coordinated at gold (**$TS_{rot\ AuC\text{-}dissoc\ P}$**), calculated at the B3PW91/SDD+f(Au),6-31G**(other atoms) level of theory. Distances are given in angstroms, bond angles and bond dihedral angles in degrees. Charge transfer (CT) from carbene to gold fragment accounting for NBO calculation. Contributions of the main atoms (in percent) in the NLMO associated to d_{xz}(Au) and $\pi_{C=CAr}$.

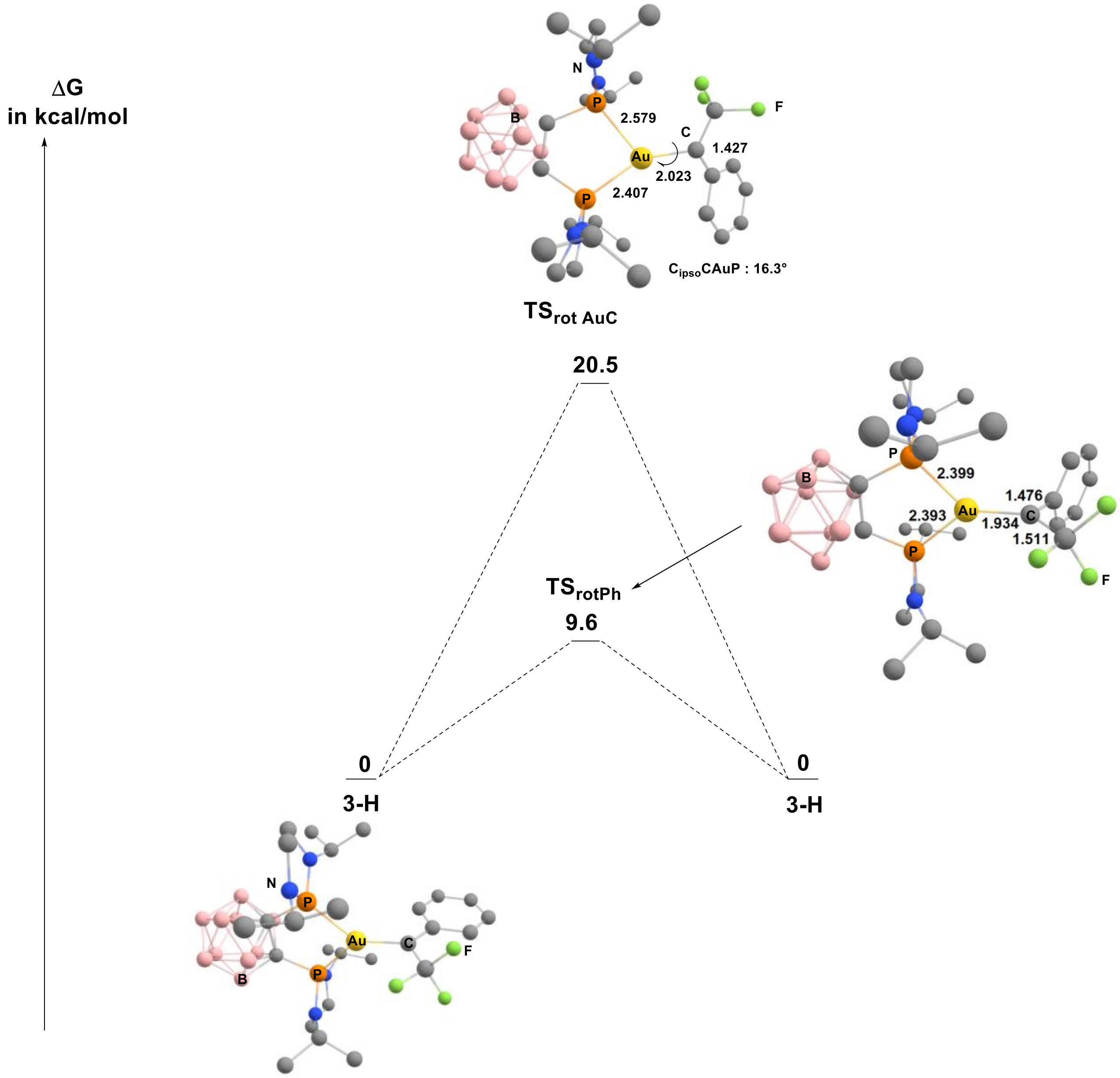

Figure S41. Energy profiles for the rotations around the Au=C bond (**TS$_{rot\ AuC}$**) and C$_{carbene}$–C$_{Ph}$ bond (**TS$_{rot\ Ph}$**), with the 2 phosphine arms coordinated to Au, calculated at the B3PW91/SDD+f(Au),6-31G**(other atoms) level of theory. Selected bond lengths in Å.

Singlet state

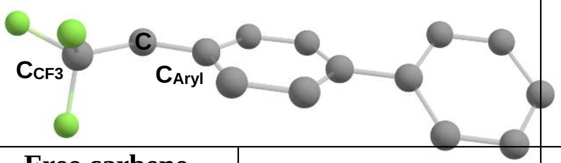

Triplet state

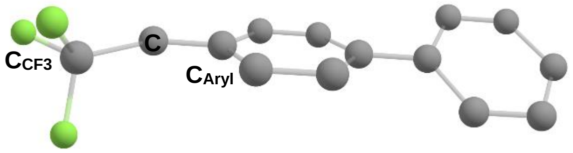

Free carbene associated to	3-CF_3		3-Ph		3-OMe	
	Singlet	Triplet	Singlet	Triplet	Singlet	Triplet
C-C_{CF3}	1.499	1.471	1.497	1.469	1.500	1.465
C-C_{Aryl}	1.429	1.396	1.421	1.393	1.415	1.395
C_{Aryl}-C-C_{CF3}	116.39	137.41	116.88	136.12	115.02	137.21
Dihedral angle Aryl/ C_{CF3}- C-C_{Aryl}	-171.9	-175.3	-175.4	176.5	179.9	-172.1
ΔG(S/T)[a]	+12.7	0	+9.9	0	+5.7	0

[a] The triplet state is more stable than the singlet state.

Table S8. Free carbenes (singlet and triplet states) associated to **3-CF_3**, **3-Ph** and **3-OMe**. Main geometrical parameters (distances in Å, bond angles and dihedral angles in °). Relative stability of the singlet and triplet states in kcal/mol (ΔG).

The singlet and triplet states of the free carbenes associated to the 3 complexes **3-CF_3**, **3-Ph** and **3-OMe** have been computed. The triplet state was found to be more stable than the singlet state (ΔG(S/T): 5.7 to 12.7 kcal/mol). The energy difference increases when the carbene becomes more electro-deficient (from R = OMe, to Ph, and CF_3), in line with a stronger stabilisation of the singlet state by π(arene) to 2p(carbene) donation for R = OMe than Ph, and CF_3. This is apparent geometrically in the C-C_{Aryl} distance that is shorter for R = OMe (1.415 Å) than Ph (1.421 Å), and CF_3 (1.429 Å). The arene substituent is perfectly coplanar with the carbene for R = OMe (dihedral angle Aryl/ C_{CF3}- C-C_{Aryl} -179.9°), but slightly twisted for Ph (-175.4°), and CF_3 (-171.9°)

7. Z-matrices and energies in au

3-OMe

Sum of electronic and zero-point Energies = -2720.045637
Sum of electronic and thermal Free Energies = -2720.131287

79	-0.502414000	-0.001447000	0.385064000
15	1.099918000	1.692186000	-0.269403000
15	1.166348000	-1.713768000	0.009242000
7	0.818756000	2.685121000	-1.598632000
7	1.424412000	2.936110000	0.782495000
5	3.568638000	0.107557000	0.657351000
1	3.071642000	0.197898000	1.731089000
5	4.252503000	1.489069000	-0.233622000
1	4.248702000	2.544738000	0.301886000
5	3.772547000	1.330178000	-1.931312000
1	3.435650000	2.280205000	-2.550701000
5	5.404812000	0.861255000	-1.423766000
1	6.343701000	1.508392000	-1.752098000
5	5.448413000	-0.905021000	-1.266424000
1	6.418699000	-1.553831000	-1.480107000
5	3.842526000	-1.532312000	-1.674628000
1	3.557076000	-2.587208000	-2.129370000
5	4.324871000	-1.373649000	0.023664000
1	4.361867000	-2.322510000	0.729234000
5	2.794142000	-0.150314000	-2.071769000
1	1.805065000	-0.243436000	-2.720517000
5	5.282851000	0.111533000	0.197200000
1	6.120039000	0.208343000	1.032403000
5	4.494178000	-0.153776000	-2.581852000
1	4.766976000	-0.252327000	-3.732518000
6	2.750932000	0.815126000	-0.669218000
6	2.793347000	-0.856573000	-0.514848000
6	0.889693000	4.105966000	-1.226926000
1	-0.114252000	4.509801000	-1.040779000
1	1.355284000	4.684721000	-2.030558000
6	1.729304000	4.170433000	0.048833000
1	2.798561000	4.237339000	-0.185236000
1	1.455521000	5.039675000	0.651160000
6	-0.043925000	2.271918000	-2.724093000
1	-0.024974000	1.174818000	-2.721746000
6	0.536467000	2.742590000	-4.056226000
1	0.533565000	3.834991000	-4.128605000
1	-0.068311000	2.361790000	-4.884666000
1	1.562492000	2.391419000	-4.186816000
6	-1.497810000	2.718160000	-2.547434000
1	-1.896570000	2.406841000	-1.576675000
1	-2.120113000	2.275652000	-3.331036000
1	-1.601457000	3.804266000	-2.631472000
6	1.304885000	2.894532000	2.252290000
1	1.105079000	1.844704000	2.499486000
6	2.604843000	3.312900000	2.936614000
1	3.443342000	2.686661000	2.623931000
1	2.499322000	3.231405000	4.022383000
1	2.853806000	4.355281000	2.710888000
6	0.113897000	3.723566000	2.736529000
1	0.256229000	4.790573000	2.535841000
1	-0.002437000	3.612611000	3.818544000
1	-0.815272000	3.401769000	2.259297000
7	1.601028000	-2.732784000	1.270569000
6	1.469262000	-4.146824000	0.890597000
1	0.522761000	-4.558240000	1.263837000
1	2.292938000	-4.732394000	1.310741000
6	1.494273000	-4.189738000	-0.638344000
1	2.519972000	-4.276262000	-1.016797000
1	0.921126000	-5.042399000	-1.010578000
6	0.013329000	-2.858161000	-2.273829000
1	-0.229967000	-1.794005000	-2.391676000
6	0.749395000	-3.317807000	-3.531370000
1	1.660490000	-2.738566000	-3.696628000
1	0.103837000	-3.204068000	-4.407247000
1	1.023781000	-4.375867000	-3.465130000
6	-1.295612000	-3.624177000	-2.067649000
1	-1.118335000	-4.698562000	-1.953594000
1	-1.948209000	-3.498100000	-2.937109000
1	-1.826769000	-3.268057000	-1.180588000
6	1.449850000	-2.346311000	2.691334000
1	1.390906000	-1.250693000	2.695472000
6	2.678813000	-2.754886000	3.500481000
1	2.789473000	-3.843706000	3.533594000
1	2.579322000	-2.407519000	4.533173000
1	3.591999000	-2.329490000	3.078364000
6	0.157816000	-2.883566000	3.310885000
1	-0.714205000	-2.634729000	2.698979000
1	0.012177000	-2.441534000	4.300388000
1	0.191850000	-3.969460000	3.443629000
7	0.870831000	-2.935993000	-1.079689000
6	-2.375031000	0.070081000	1.047008000
6	-3.583485000	-0.013328000	0.300369000
6	-4.884971000	0.127409000	0.862313000
6	-3.519230000	-0.252073000	-1.104912000
6	-6.025391000	0.044833000	0.090089000
1	-5.000840000	0.310124000	1.922932000
6	-4.643523000	-0.339132000	-1.883762000
1	-2.539700000	-0.374225000	-1.558709000
6	-5.919399000	-0.188954000	-1.294640000
1	-6.995073000	0.161488000	0.559232000
1	-4.589320000	-0.524599000	-2.951144000
8	-6.951549000	-0.286173000	-2.129091000
6	-8.281630000	-0.149756000	-1.627316000
1	-8.932692000	-0.265404000	-2.492496000
1	-8.504848000	-0.930298000	-0.893128000
1	-8.432736000	0.839545000	-1.183892000
6	-2.507273000	0.277297000	2.542192000
9	-3.115640000	1.447740000	2.827811000
9	-1.306011000	0.311363000	3.163603000
9	-3.210965000	-0.710331000	3.129586000

3-Ph

Sum of electronic and zero-point Energies = -2836.485369
Sum of electronic and thermal Free Energies = -2836.576000

79	-0.090023000	-0.073543000	0.615273000
15	-1.659187000	-1.674228000	-0.291312000
15	-1.493488000	1.735106000	-0.161825000
7	-1.172268000	-2.805435000	-1.436644000
7	-2.340042000	-2.783916000	0.739566000
5	-4.132441000	0.144925000	-0.108491000
1	-3.937609000	0.079726000	1.059652000
5	-4.687387000	-1.226670000	-1.097750000
1	-4.920650000	-2.245026000	-0.540188000
5	-3.771959000	-1.206079000	-2.615065000
1	-3.377672000	-2.214302000	-3.091839000
5	-5.427660000	-0.574817000	-2.573065000
1	-6.305336000	-1.161020000	-3.115232000
5	-5.341462000	1.195122000	-2.494691000
1	-6.157250000	1.907666000	-2.979306000
5	-3.632031000	1.663009000	-2.485012000
1	-3.137648000	2.663176000	-2.880698000
5	-4.554578000	1.643317000	-0.971920000
1	-4.686406000	2.632137000	-0.336362000

5	-2.653549000	0.177252000	-2.543503000
1	-1.525442000	0.147528000	-2.911332000
5	-5.659903000	0.255207000	-1.004622000
1	-6.690991000	0.276655000	-0.417856000
5	-4.154078000	0.292849000	-3.483844000
1	-4.107852000	0.344609000	-4.668450000
6	-3.068296000	-0.703295000	-1.145229000
6	-2.992468000	0.971548000	-1.067104000
6	-1.466195000	-4.176052000	-0.991332000
1	-0.575869000	-4.637802000	-0.545216000
1	-1.788559000	-4.787822000	-1.839487000
6	-2.578082000	-4.058655000	0.051255000
1	-3.567476000	-4.075498000	-0.421048000
1	-2.527338000	-4.882378000	0.767019000
6	-0.023148000	-2.569193000	-2.335553000
1	0.069391000	-1.478633000	-2.412460000
6	-0.306829000	-3.112018000	-3.734495000
1	-0.407541000	-4.202101000	-3.725484000
1	0.521555000	-2.868620000	-4.406452000
1	-1.223744000	-2.684846000	-4.146588000
6	1.289424000	-3.115067000	-1.766921000
1	1.462972000	-2.756178000	-0.747416000
1	2.128129000	-2.790855000	-2.390192000
1	1.303007000	-4.209167000	-1.756218000
6	-2.568531000	-2.613985000	2.187026000
1	-2.334611000	-1.563464000	2.399858000
6	-4.027554000	-2.869203000	2.560266000
1	-4.705117000	-2.220636000	2.000239000
1	-4.178154000	-2.686554000	3.628243000
1	-4.310705000	-3.908974000	2.364857000
6	-1.610053000	-3.480484000	3.006028000
1	-1.803656000	-4.548061000	2.858368000
1	-1.740160000	-3.274671000	4.072490000
1	-0.568582000	-3.277512000	2.743552000
7	-2.148080000	2.836288000	0.922443000
6	-1.798080000	4.218248000	0.562970000
1	-0.942671000	4.565191000	1.156848000
1	-2.645558000	4.884862000	0.750424000
6	-1.438548000	4.197968000	-0.923228000
1	-2.327458000	4.344042000	-1.548689000
1	-0.721935000	4.988048000	-1.159669000
6	0.317190000	2.696874000	-2.067509000
1	0.491927000	1.613615000	-2.098822000
6	0.002234000	3.167514000	-3.486203000
1	-0.873646000	2.655639000	-3.891357000
1	0.853286000	2.972032000	-4.145219000
1	-0.188350000	4.245472000	-3.512624000
6	1.574513000	3.363075000	-1.505189000
1	1.465755000	4.451503000	-1.456813000
1	2.433884000	3.154841000	-2.149769000
1	1.798671000	2.993854000	-0.500553000
6	-2.446500000	2.505083000	2.333923000
1	-2.473260000	1.409356000	2.384064000
6	-3.823951000	3.027238000	2.738558000
1	-3.861001000	4.120918000	2.702972000
1	-4.049206000	2.728430000	3.766628000
1	-4.606680000	2.635847000	2.085380000
6	-1.357260000	2.991494000	3.292570000
1	-0.363190000	2.659267000	2.980994000
1	-1.545757000	2.594113000	4.293806000
1	-1.349779000	4.082838000	3.375305000
7	-0.833565000	2.882432000	-1.167422000
6	1.624183000	-0.164719000	1.607725000
6	2.953896000	-0.153857000	1.085609000
6	4.128963000	-0.191345000	1.888478000
6	3.147138000	-0.087734000	-0.321163000
6	5.384966000	-0.153614000	1.318920000
1	4.050295000	-0.258930000	2.965835000
6	4.402605000	-0.054278000	-0.884986000
1	2.267137000	-0.049920000	-0.957585000
6	5.561822000	-0.080914000	-0.078143000
1	6.256449000	-0.213188000	1.962655000
1	4.505017000	0.030578000	-1.962062000
6	1.482083000	-0.233148000	3.114407000
9	2.044411000	-1.348818000	3.619771000
9	0.186256000	-0.249455000	3.503199000
9	2.047677000	0.830636000	3.719746000
6	6.904045000	-0.030530000	-0.674522000
6	7.155583000	-0.607002000	-1.932199000
6	7.965806000	0.598818000	-0.000844000
6	8.426368000	-0.560792000	-2.492376000
1	6.360666000	-1.129905000	-2.455915000
6	9.232603000	0.654727000	-0.569209000
1	7.788805000	1.080574000	0.956126000
6	9.467960000	0.072934000	-1.814802000
1	8.607614000	-1.025908000	-3.456592000
1	10.037302000	1.158338000	-0.042343000
1	10.459452000	0.112562000	-2.255619000

3-CF_3

Sum of electronic and zero-point Energies = -2942.490329

Sum of electronic and thermal Free Energies = -2942.580013

79	0.166383000	-0.065814000	0.545710000
15	-1.432991000	-1.679655000	-0.265160000
15	-1.329089000	1.716227000	-0.077662000
7	-0.997246000	-2.835646000	-1.403597000
7	-2.042030000	-2.762203000	0.834968000
5	-3.933831000	0.069459000	0.035199000
1	-3.698100000	-0.018154000	1.193597000
5	-4.485631000	-1.290737000	-0.971459000
1	-4.674500000	-2.328621000	-0.433835000
5	-3.621486000	-1.210315000	-2.518257000
1	-3.222078000	-2.198606000	-3.029279000
5	-5.288049000	-0.619239000	-2.404901000
1	-6.168952000	-1.212642000	-2.933223000
5	-5.240255000	1.149702000	-2.283729000
1	-6.086927000	1.854922000	-2.723318000
5	-3.542498000	1.658247000	-2.316997000
1	-3.083084000	2.680400000	-2.698324000
5	-4.414849000	1.578353000	-0.776043000
1	-4.545110000	2.547387000	-0.110291000
5	-2.532571000	0.197574000	-2.448521000
1	-1.414748000	0.206245000	-2.848378000
5	-5.489220000	0.165516000	-0.808936000
1	-6.500529000	0.147111000	-0.189150000
5	-4.065781000	0.301655000	-3.334575000
1	-4.059676000	0.383952000	-4.518019000
6	-2.882292000	-0.725271000	-1.058411000
6	-2.841786000	0.941166000	-0.939385000
6	-1.225422000	-4.195687000	-0.888912000
1	-0.299832000	-4.614230000	-0.473229000
1	-1.573047000	-4.848001000	-1.695472000
6	-2.285559000	-4.065736000	0.203127000
1	-3.296414000	-4.116921000	-0.218015000
1	-2.180918000	-4.862108000	0.943297000
6	0.084540000	-2.611836000	-2.388082000
1	0.134166000	-1.524947000	-2.528840000
6	-0.266920000	-3.238552000	-3.735615000
1	-0.318917000	-4.329802000	-3.667079000
1	0.506311000	-2.997728000	-4.470959000
1	-1.224653000	-2.869580000	-4.108615000
6	1.446162000	-3.090576000	-1.877677000

1	1.670154000	-2.680867000	-0.887570000
1	2.234474000	-2.772598000	-2.566248000
1	1.494920000	-4.182026000	-1.818710000
6	-2.207010000	-2.549281000	2.286542000
1	-1.988511000	-1.487085000	2.453777000
6	-3.640804000	-2.827398000	2.733379000
1	-4.360357000	-2.218000000	2.181927000
1	-3.749504000	-2.610274000	3.799931000
1	-3.902898000	-3.880610000	2.587823000
6	-1.191521000	-3.366503000	3.086985000
1	-1.366422000	-4.442214000	2.982796000
1	-1.278320000	-3.128546000	4.151082000
1	-0.167964000	-3.149267000	2.771772000
7	-1.951385000	2.766507000	1.069669000
6	-1.618219000	4.163669000	0.749446000
1	-0.736656000	4.489040000	1.316226000
1	-2.457698000	4.817852000	1.003431000
6	-1.331199000	4.201579000	-0.751687000
1	-2.250293000	4.361756000	-1.327892000
1	-0.632020000	5.005156000	-0.993794000
6	0.393770000	2.761433000	-2.017928000
1	0.574861000	1.681765000	-2.099259000
6	0.028859000	3.286414000	-3.404952000
1	-0.855647000	2.783669000	-3.802862000
1	0.859682000	3.124680000	-4.097881000
1	-0.169598000	4.362974000	-3.382119000
6	1.662701000	3.414179000	-1.467210000
1	1.549289000	4.498837000	-1.371448000
1	2.503330000	3.238397000	-2.145112000
1	1.919487000	3.005914000	-0.485566000
6	-2.204976000	2.381957000	2.477425000
1	-2.223725000	1.284832000	2.487311000
6	-3.571763000	2.880270000	2.942428000
1	-3.614280000	3.974133000	2.953432000
1	-3.764930000	2.538656000	3.963567000
1	-4.372117000	2.511710000	2.297428000
6	-1.087225000	2.840689000	3.416136000
1	-0.102878000	2.523462000	3.061186000
1	-1.241571000	2.409011000	4.408917000
1	-1.080795000	3.928512000	3.535529000
7	-0.730843000	2.900233000	-1.074576000
6	1.939423000	-0.143580000	1.403620000
6	3.238253000	-0.105805000	0.777421000
6	4.460520000	-0.127131000	1.495531000
6	3.314738000	-0.037476000	-0.637403000
6	5.678605000	-0.078783000	0.833173000
1	4.461103000	-0.179341000	2.576221000
6	4.526488000	0.007041000	-1.296812000
1	2.388951000	-0.023689000	-1.205243000
6	5.716324000	-0.011831000	-0.558442000
1	6.604672000	-0.094988000	1.396631000
1	4.563156000	0.054256000	-2.380353000
6	1.914898000	-0.233909000	2.915270000
9	2.543378000	-1.339174000	3.359510000
9	0.653456000	-0.290476000	3.400495000
9	2.496565000	0.838930000	3.487303000
6	7.028798000	0.063078000	-1.301512000
9	7.089059000	-0.885721000	-2.251979000
9	7.153281000	1.253546000	-1.914472000
9	8.078836000	-0.095896000	-0.486127000

3-H
Sum of electronic and zero-point Energies = -2605.586123
Sum of electronic and thermal Free Energies = -2605.668607

79	0.825203000	0.025361000	0.221720000
15	-0.824651000	-1.707008000	-0.106808000
15	-0.903999000	1.694544000	0.019110000
7	-0.634245000	-2.847570000	-1.326453000
7	-1.048054000	-2.815577000	1.108138000
5	-3.269963000	-0.108133000	0.842494000
1	-2.729359000	-0.149056000	1.896908000
5	-3.973902000	-1.527644000	0.030120000
1	-3.942563000	-2.562105000	0.605578000
5	-3.556194000	-1.432552000	-1.689999000
1	-3.239072000	-2.407428000	-2.278727000
5	-5.171435000	-0.950088000	-1.143935000
1	-6.117425000	-1.613523000	-1.413121000
5	-5.218558000	0.820967000	-1.053970000
1	-6.199055000	1.456991000	-1.258300000
5	-3.631539000	1.438339000	-1.542612000
1	-3.364359000	2.476956000	-2.043538000
5	-4.053330000	1.344057000	0.177175000
1	-4.066220000	2.319924000	0.846197000
5	-2.591121000	0.045856000	-1.925342000
1	-1.621599000	0.121841000	-2.606023000
5	-4.997546000	-0.137190000	0.441836000
1	-5.804812000	-0.204820000	1.308596000
5	-4.307010000	0.022902000	-2.372781000
1	-4.621668000	0.074217000	-3.515615000
6	-2.494681000	-0.862467000	-0.485018000
6	-2.540858000	0.807042000	-0.396189000
6	-0.625902000	-4.212722000	-0.777619000
1	0.402817000	-4.562804000	-0.622167000
1	-1.128059000	-4.897536000	-1.467415000
6	-1.366278000	-4.138958000	0.556461000
1	-2.447097000	-4.259650000	0.416929000
1	-1.018445000	-4.918735000	1.237797000
6	0.118134000	-2.563942000	-2.568202000
1	0.084512000	-1.474122000	-2.690580000
6	-0.569459000	-3.190305000	-3.779701000
1	-0.565045000	-4.283440000	-3.719792000
1	-0.036009000	-2.914789000	-4.694266000
1	-1.604197000	-2.853498000	-3.869531000
6	1.586613000	-2.985879000	-2.473124000
1	2.066059000	-2.578257000	-1.577895000
1	2.134042000	-2.622218000	-3.347557000
1	1.694000000	-4.074735000	-2.458241000
6	-0.857435000	-2.592164000	2.554454000
1	-0.690916000	-1.513533000	2.666538000
6	-2.105356000	-2.974568000	3.348160000
1	-2.986878000	-2.432183000	2.998973000
1	-1.958404000	-2.749735000	4.408479000
1	-2.309982000	-4.047440000	3.268707000
6	0.388161000	-3.315208000	3.069849000
1	0.278595000	-4.402690000	3.006067000
1	0.551312000	-3.070048000	4.123430000
1	1.280243000	-3.022838000	2.510662000
7	-1.257283000	2.703448000	1.310920000
6	-1.170232000	4.120148000	0.923494000
1	-0.203974000	4.542749000	1.226763000
1	-1.968100000	4.694263000	1.404607000
6	-1.313532000	4.159062000	-0.599093000
1	-2.367117000	4.225013000	-0.895769000
1	-0.787232000	5.020276000	-1.016899000
6	0.130770000	2.866006000	-2.301413000
1	0.389368000	1.807570000	-2.434516000
6	-0.641684000	3.324005000	-3.536792000
1	-1.542955000	2.725786000	-3.688257000
1	-0.012814000	3.234313000	-4.427387000
1	-0.937947000	4.374815000	-3.452860000
6	1.430718000	3.650143000	-2.111057000
1	1.240535000	4.720832000	-1.984042000

1	2.070045000	3.540731000	-2.992294000
1	1.983216000	3.291977000	-1.237897000
6	-1.035176000	2.317828000	2.722662000
1	-0.940033000	1.224331000	2.719445000
6	-2.241336000	2.684586000	3.584719000
1	-2.385045000	3.769129000	3.625505000
1	-2.086533000	2.339469000	4.611295000
1	-3.158122000	2.231360000	3.201243000
6	0.262544000	2.897570000	3.289264000
1	1.118064000	2.681122000	2.643396000
1	0.462646000	2.459597000	4.271091000
1	0.195579000	3.981168000	3.427233000
7	-0.707548000	2.914163000	-1.090386000
6	2.773676000	0.016103000	0.542749000
6	3.838372000	0.065377000	-0.422978000
6	5.216050000	-0.012418000	-0.084389000
6	3.509676000	0.200330000	-1.797085000
6	6.192069000	0.042246000	-1.067243000
1	5.519916000	-0.119056000	0.948758000
6	4.486829000	0.255529000	-2.773880000
1	2.459892000	0.264822000	-2.070311000
6	5.835304000	0.176510000	-2.410320000
1	7.239020000	-0.019770000	-0.787816000
1	4.210349000	0.361770000	-3.818270000
6	3.175698000	-0.087011000	1.999899000
9	3.850042000	-1.228532000	2.245701000
9	2.101842000	-0.093071000	2.822757000
9	3.945916000	0.947277000	2.386260000
1	6.606118000	0.220486000	-3.174079000

3-H$_{dissoc\ P}$

Sum of electronic and zero-point Energies = -2605.558006
Sum of electronic and thermal Free Energies = -2605.642146

79	-1.100315000	-0.947018000	0.022543000
15	0.872926000	1.938408000	-0.025112000
15	1.098879000	-1.557647000	0.552160000
7	0.216860000	2.827714000	-1.319479000
7	1.630775000	3.285695000	0.638508000
5	3.530012000	0.341196000	0.172756000
1	3.425476000	0.551798000	1.332883000
5	3.894920000	1.581013000	-1.039026000
1	4.096598000	2.679963000	-0.648759000
5	2.874182000	1.287470000	-2.452872000
1	2.368024000	2.190121000	-3.027424000
5	4.574566000	0.780950000	-2.468015000
1	5.361886000	1.342879000	-3.156053000
5	4.633721000	-0.961439000	-2.141957000
1	5.462688000	-1.682466000	-2.591247000
5	2.971944000	-1.531621000	-1.928623000
1	2.540597000	-2.608873000	-2.152464000
5	3.998153000	-1.236229000	-0.509928000
1	4.262050000	-2.122335000	0.230103000
5	1.876811000	-0.142220000	-2.105169000
1	0.727095000	-0.252272000	-2.381643000
5	4.986208000	0.200029000	-0.827989000
1	6.056883000	0.329804000	-0.332455000
5	3.309574000	-0.286972000	-3.140630000
1	3.179551000	-0.514997000	-4.298141000
6	2.315045000	0.952983000	-0.873443000
6	2.384559000	-0.692486000	-0.560581000
6	0.528639000	4.258538000	-1.241023000
1	-0.321292000	4.817169000	-0.825490000
1	0.747236000	4.660920000	-2.236226000
6	1.742409000	4.385650000	-0.322535000
1	2.675734000	4.317216000	-0.894197000
1	1.736457000	5.347422000	0.197106000
6	-0.996241000	2.407123000	-2.034847000
1	-1.116022000	1.341176000	-1.803941000
6	-0.830963000	2.534061000	-3.549719000
1	-0.706629000	3.579850000	-3.849479000
1	-1.720651000	2.151215000	-4.058861000
1	0.037378000	1.972013000	-3.899706000
6	-2.252300000	3.140026000	-1.547829000
1	-2.350059000	3.082569000	-0.458556000
1	-3.141848000	2.692188000	-1.998317000
1	-2.243631000	4.195737000	-1.835267000
6	1.928717000	3.469916000	2.064733000
1	1.835118000	2.475050000	2.515533000
6	3.363241000	3.948894000	2.285567000
1	4.083177000	3.263724000	1.831612000
1	3.578390000	4.019575000	3.356015000
1	3.522283000	4.943631000	1.856053000
6	0.910040000	4.390309000	2.744108000
1	0.949939000	5.405776000	2.336370000
1	1.115763000	4.465028000	3.816532000
1	-0.108413000	4.011307000	2.613299000
7	1.552148000	-1.408096000	2.150277000
6	2.399341000	-2.536393000	2.562316000
1	2.232029000	-2.756284000	3.619559000
1	3.459056000	-2.293167000	2.431511000
6	1.990517000	-3.731459000	1.696458000
1	2.846920000	-4.389741000	1.521028000
1	1.204140000	-4.313308000	2.195966000
6	0.835463000	-4.093912000	-0.537818000
1	0.603239000	-3.477873000	-1.414605000
6	1.796172000	-5.194362000	-0.983464000
1	2.737906000	-4.775452000	-1.345623000
1	1.343899000	-5.776223000	-1.791370000
1	2.016386000	-5.889785000	-0.166810000
6	-0.476854000	-4.674644000	-0.004032000
1	-0.313922000	-5.329064000	0.858047000
1	-0.960430000	-5.274720000	-0.780557000
1	-1.174804000	-3.882576000	0.290070000
6	0.928911000	-0.519827000	3.145268000
1	0.514011000	0.320494000	2.574343000
6	1.970692000	0.036378000	4.115880000
1	2.387145000	-0.749686000	4.753625000
1	1.508576000	0.775265000	4.777105000
1	2.797758000	0.513530000	3.584534000
6	-0.214038000	-1.216498000	3.889079000
1	-0.978062000	-1.575525000	3.191790000
1	-0.686308000	-0.529677000	4.599164000
1	0.149560000	-2.075386000	4.463034000
7	1.497549000	-3.186179000	0.426171000
6	-2.995852000	-0.420713000	-0.434285000
6	-3.927801000	0.221306000	0.410235000
6	-5.283124000	0.500167000	0.041327000
6	-3.504102000	0.592333000	1.723921000
6	-6.143775000	1.097685000	0.939601000
1	-5.645410000	0.232598000	-0.942688000
6	-4.371024000	1.195619000	2.611317000
1	-2.475978000	0.384046000	2.004803000
6	-5.692781000	1.446120000	2.219915000
1	-7.171758000	1.297080000	0.655521000
1	-4.037131000	1.470172000	3.606411000
6	-3.468363000	-0.832131000	-1.825378000
9	-4.438133000	-1.757303000	-1.719131000
9	-2.480151000	-1.364132000	-2.556404000
9	-3.952220000	0.215278000	-2.524187000
1	-6.379223000	1.915625000	2.919053000

TS$_{rot\ Ph}$
Sum of electronic and zero-point Energies = -2605.571552
Sum of electronic and thermal Free Energies = -2605.653336

79	0.810463000	-0.006312000	-0.214208000
15	-0.849147000	1.685424000	0.106988000
15	-0.873149000	-1.688575000	0.083409000
7	-0.824590000	2.695533000	1.442962000
7	-0.867501000	2.900701000	-1.018331000
5	-3.000550000	0.033840000	-1.311163000
1	-2.227742000	0.046852000	-2.212704000
5	-3.881613000	1.473445000	-0.739914000
1	-3.720381000	2.487715000	-1.327800000
5	-3.871565000	1.450708000	1.032274000
1	-3.697811000	2.448327000	1.644486000
5	-5.313769000	0.925036000	0.146948000
1	-6.298650000	1.585949000	0.163194000
5	-5.331016000	-0.848854000	0.122354000
1	-6.328529000	-1.490710000	0.120004000
5	-3.895529000	-1.425508000	0.989349000
1	-3.745704000	-2.440215000	1.581299000
5	-3.913894000	-1.402467000	-0.782757000
1	-3.777278000	-2.407676000	-1.388287000
5	-2.985320000	-0.003023000	1.546428000
1	-2.207926000	-0.028285000	2.438830000
5	-4.774391000	0.053297000	-1.320304000
1	-5.359114000	0.075132000	-2.352076000
5	-4.755652000	0.011686000	1.581151000
1	-5.325565000	-0.000192000	2.621461000
6	-2.556377000	0.842829000	0.130232000
6	-2.572206000	-0.823035000	0.099986000
6	-0.732509000	4.107146000	1.036451000
1	0.308255000	4.454429000	1.072523000
1	-1.332978000	4.728823000	1.706911000
6	-1.268692000	4.168715000	-0.393665000
1	-2.359262000	4.283957000	-0.403764000
1	-0.829939000	5.006153000	-0.940032000
6	-0.336427000	2.281426000	2.776569000
1	-0.394705000	1.186363000	2.783787000
6	-1.255168000	2.805685000	3.878278000
1	-1.226544000	3.898515000	3.935708000
1	-0.928830000	2.422819000	4.849575000
1	-2.289822000	2.496302000	3.714240000
6	1.122663000	2.672945000	3.016962000
1	1.778013000	2.299726000	2.224354000
1	1.467012000	2.247530000	3.964118000
1	1.244973000	3.758093000	3.088867000
6	-0.396770000	2.816244000	-2.416802000
1	-0.167027000	1.757057000	-2.584617000
6	-1.482856000	3.238477000	-3.403665000
1	-2.382625000	2.628705000	-3.297633000
1	-1.115466000	3.134639000	-4.428645000
1	-1.761229000	4.288100000	-3.261513000
6	0.894233000	3.611682000	-2.616502000
1	0.729923000	4.688324000	-2.504176000
1	1.275992000	3.449331000	-3.628401000
1	1.667580000	3.302882000	-1.908330000
7	-1.002306000	-2.909380000	-1.054962000
6	-0.843468000	-4.232743000	-0.430829000
1	0.188834000	-4.587914000	-0.541245000
1	-1.513947000	-4.954374000	-0.906776000
6	-1.187933000	-4.057468000	1.047506000
1	-2.262371000	-4.185259000	1.224691000
1	-0.649696000	-4.785657000	1.658886000
6	-0.233680000	-2.355187000	2.732094000
1	-0.107248000	-1.265221000	2.730539000
6	-1.231379000	-2.722588000	3.828599000
1	-2.203444000	-2.252622000	3.662272000
1	-0.852692000	-2.399014000	4.802237000
1	-1.379461000	-3.806079000	3.881945000
6	1.139542000	-2.984397000	2.969268000
1	1.088642000	-4.077985000	2.960788000
1	1.523639000	-2.686141000	3.949334000
1	1.857856000	-2.663168000	2.210372000
6	-0.611187000	-2.729799000	-2.475837000
1	-0.605608000	-1.646312000	-2.646723000
6	-1.644472000	-3.353016000	-3.411368000
1	-1.704962000	-4.437319000	-3.272191000
1	-1.355723000	-3.175572000	-4.451459000
1	-2.637832000	-2.929168000	-3.253228000
6	0.794883000	-3.259946000	-2.763073000
1	1.530519000	-2.868180000	-2.055409000
1	1.102410000	-2.956176000	-3.767242000
1	0.828763000	-4.353435000	-2.731398000
7	-0.753120000	-2.696794000	1.393572000
6	2.700870000	-0.010473000	-0.620700000
6	3.788702000	-0.013491000	0.377034000
6	4.312177000	1.196259000	0.859218000
6	4.321216000	-1.225727000	0.842970000
6	5.342820000	1.187543000	1.795190000
1	3.916488000	2.138306000	0.492244000
6	5.351729000	-1.221487000	1.779191000
1	3.932796000	-2.165810000	0.463628000
6	5.864186000	-0.018200000	2.259724000
1	5.741742000	2.130318000	2.157822000
1	5.757644000	-2.165974000	2.129380000
6	3.177401000	-0.011035000	-2.054558000
9	3.973384000	1.042460000	-2.297131000
9	2.157151000	0.050789000	-2.939694000
9	3.864315000	-1.135835000	-2.319857000
1	6.668588000	-0.020053000	2.988603000

TS$_{rot\ AuC}$
Sum of electronic and zero-point Energies = -2605.556095
Sum of electronic and thermal Free Energies = -2605.635887

79	0.908986000	0.111204000	-0.487038000
15	-0.826188000	-1.679184000	0.172122000
15	-0.793697000	1.783854000	-0.174312000
7	-1.304868000	-2.829790000	-0.961467000
7	-0.601535000	-2.786078000	1.397596000
5	-2.443279000	0.275980000	1.950465000
1	-1.444573000	0.323217000	2.589236000
5	-3.478869000	-1.163532000	1.827661000
1	-3.182962000	-2.116081000	2.463483000
5	-3.977702000	-1.326630000	0.138676000
1	-4.016842000	-2.395261000	-0.365151000
5	-5.096380000	-0.641952000	1.327737000
1	-6.056271000	-1.252571000	1.665265000
5	-5.074180000	1.121559000	1.152157000
1	-6.015858000	1.811766000	1.364344000
5	-3.937025000	1.529524000	-0.144939000
1	-3.948682000	2.465772000	-0.868054000
5	-3.445572000	1.693211000	1.548357000
1	-3.124859000	2.747958000	1.975235000
5	-3.241108000	0.018663000	-0.768869000
1	-2.745459000	-0.075611000	-1.841809000
5	-4.141249000	0.349164000	2.471279000
1	-4.403585000	0.464389000	3.622849000
5	-4.953883000	0.085149000	-0.301680000
1	-5.797924000	0.017371000	-1.133060000
6	-2.445494000	-0.706822000	0.550855000
6	-2.421560000	0.961669000	0.396451000

6	-1.280059000	-4.189216000	-0.404138000
1	-0.365269000	-4.713017000	-0.709233000
1	-2.142866000	-4.758896000	-0.764415000
6	-1.329470000	-4.030264000	1.114314000
1	-2.364394000	-3.975909000	1.473237000
1	-0.838039000	-4.869987000	1.609778000
6	-1.353217000	-2.651247000	-2.430810000
1	-1.066366000	-1.608196000	-2.616779000
6	-2.759434000	-2.880153000	-2.989996000
1	-3.099303000	-3.901250000	-2.788419000
1	-2.747522000	-2.751657000	-4.076642000
1	-3.492326000	-2.190166000	-2.572440000
6	-0.358164000	-3.556619000	-3.162554000
1	0.653720000	-3.468791000	-2.770787000
1	-0.337872000	-3.284108000	-4.221626000
1	-0.663858000	-4.606432000	-3.109176000
6	0.222227000	-2.613785000	2.609716000
1	0.724159000	-1.645651000	2.483012000
6	-0.612419000	-2.569841000	3.889864000
1	-1.328313000	-1.746345000	3.885719000
1	0.043289000	-2.449628000	4.757728000
1	-1.167727000	-3.503721000	4.026992000
6	1.301687000	-3.695058000	2.704153000
1	0.875285000	-4.671514000	2.955169000
1	2.001671000	-3.441934000	3.505864000
1	1.862915000	-3.785541000	1.773287000
7	-0.514653000	2.986014000	0.969061000
6	-0.700244000	4.329976000	0.402782000
1	0.270734000	4.784270000	0.165999000
1	-1.216963000	4.975796000	1.120197000
6	-1.530380000	4.159327000	-0.871769000
1	-2.603496000	4.228157000	-0.657695000
1	-1.280172000	4.931748000	-1.602989000
6	-1.019686000	2.573274000	-2.842587000
1	-0.823910000	1.496673000	-2.922524000
6	-2.301236000	2.883337000	-3.613684000
1	-3.149660000	2.317406000	-3.222112000
1	-2.174196000	2.628809000	-4.669926000
1	-2.547800000	3.949181000	-3.563050000
6	0.189350000	3.313302000	-3.417898000
1	0.059837000	4.399539000	-3.369608000
1	0.325599000	3.050890000	-4.471335000
1	1.104035000	3.048684000	-2.879552000
6	0.255502000	2.825535000	2.220209000
1	0.498059000	1.757478000	2.290633000
6	-0.560268000	3.218106000	3.452208000
1	-0.855390000	4.271595000	3.410657000
1	0.044252000	3.085298000	4.354742000
1	-1.463490000	2.615487000	3.553036000
6	1.572228000	3.603792000	2.182072000
1	2.142991000	3.385346000	1.277431000
1	2.186551000	3.331142000	3.044788000
1	1.402273000	4.683817000	2.233963000
7	-1.177675000	2.837799000	-1.400674000
6	2.842660000	-0.479090000	-0.545798000
6	3.899716000	0.297608000	0.016601000
6	5.086209000	-0.253250000	0.592092000
6	3.803893000	1.715795000	-0.062376000
6	6.083283000	0.571508000	1.073725000
1	5.196240000	-1.326192000	0.682959000
6	4.817817000	2.534165000	0.405401000
1	2.933672000	2.129048000	-0.562147000
6	5.956967000	1.964150000	0.977687000
1	6.971729000	0.139187000	1.522804000
1	4.738329000	3.612277000	0.309615000
6	3.189345000	-1.916967000	-0.857883000
9	4.322313000	-2.032247000	-1.567733000
9	2.220603000	-2.528409000	-1.559332000
9	3.346256000	-2.626959000	0.287471000
1	6.758242000	2.602855000	1.338059000

TS$_{rot\ AuC\text{-}dissocP}$

Sum of electronic and zero-point Energies = -2605.547952

Sum of electronic and thermal Free Energies = -2605.631169

79	-1.406198000	-0.365216000	-0.214381000
15	1.501958000	1.723570000	0.159250000
15	0.401589000	-1.876557000	-0.037373000
7	2.102971000	2.828817000	-0.982672000
7	2.019352000	2.646952000	1.463571000
5	2.935727000	-0.769147000	1.418149000
1	2.224982000	-0.571813000	2.346683000
5	4.261709000	0.312104000	0.951355000
1	4.457976000	1.253702000	1.639158000
5	4.259963000	0.430756000	-0.812343000
1	4.455964000	1.466229000	-1.347291000
5	5.436928000	-0.615128000	0.000921000
1	6.588295000	-0.326413000	0.013135000
5	4.855300000	-2.281630000	-0.106551000
1	5.570803000	-3.226897000	-0.171102000
5	3.319951000	-2.270143000	-0.985519000
1	2.863986000	-3.133512000	-1.650810000
5	3.328237000	-2.389330000	0.781607000
1	2.874014000	-3.333015000	1.330931000
5	2.921492000	-0.585178000	-1.408498000
1	2.189598000	-0.286028000	-2.294775000
5	4.619046000	-1.358701000	1.406478000
1	5.164117000	-1.615376000	2.429388000
5	4.607172000	-1.168295000	-1.484775000
1	5.144352000	-1.295555000	-2.535729000
6	2.812221000	0.270340000	0.061297000
6	2.249140000	-1.338219000	-0.030320000
6	2.720635000	4.010922000	-0.374186000
1	2.035796000	4.867478000	-0.430886000
1	3.644882000	4.281584000	-0.897595000
6	3.009330000	3.657848000	1.086927000
1	4.030097000	3.275193000	1.203592000
1	2.908897000	4.541198000	1.723553000
6	1.626837000	2.911056000	-2.375711000
1	0.961101000	2.050756000	-2.516024000
6	2.772175000	2.793803000	-3.384259000
1	3.496541000	3.604796000	-3.255722000
1	2.380508000	2.865090000	-4.403964000
1	3.303245000	1.845680000	-3.286743000
6	0.815783000	4.184994000	-2.641609000
1	0.041153000	4.338852000	-1.887342000
1	0.330063000	4.112486000	-3.619099000
1	1.457338000	5.071456000	-2.667235000
6	1.376452000	2.641882000	2.784894000
1	0.754697000	1.737676000	2.805649000
6	2.404982000	2.531098000	3.910001000
1	3.035311000	1.647327000	3.784080000
1	1.901750000	2.463884000	4.879312000
1	3.055562000	3.411483000	3.941446000
6	0.451703000	3.848273000	2.973053000
1	1.010496000	4.789903000	2.956837000
1	-0.058150000	3.792042000	3.940128000
1	-0.306182000	3.886225000	2.185389000
7	0.275096000	-2.900112000	1.291698000
6	0.064630000	-4.297014000	0.893135000
1	-0.995754000	-4.569799000	0.983940000
1	0.649436000	-4.967309000	1.531294000
6	0.518772000	-4.405381000	-0.565284000

1	1.581902000	-4.665017000	-0.630387000
1	-0.050972000	-5.174569000	-1.092473000
6	-0.260304000	-2.938411000	-2.539046000
1	-0.260821000	-1.858911000	-2.734870000
6	0.646778000	-3.604656000	-3.571676000
1	1.659403000	-3.198214000	-3.537196000
1	0.245763000	-3.442918000	-4.576434000
1	0.704630000	-4.686809000	-3.414255000
6	-1.703534000	-3.436272000	-2.645521000
1	-1.774867000	-4.512343000	-2.455481000
1	-2.094152000	-3.256884000	-3.651781000
1	-2.347477000	-2.915635000	-1.929411000
6	-0.139144000	-2.443482000	2.631823000
1	0.077601000	-1.368655000	2.659677000
6	0.692074000	-3.113728000	3.724585000
1	0.495960000	-4.189730000	3.773910000
1	0.434307000	-2.692699000	4.700799000
1	1.760684000	-2.965295000	3.555468000
6	-1.639937000	-2.623578000	2.875934000
1	-2.227358000	-2.134243000	2.091095000
1	-1.919347000	-2.178668000	3.835672000
1	-1.922058000	-3.680481000	2.912488000
7	0.256781000	-3.093363000	-1.166100000
6	-2.954602000	0.929310000	-0.365867000
6	-4.285171000	0.649046000	0.016785000
6	-5.292796000	1.648094000	0.222326000
6	-4.648102000	-0.716487000	0.242957000
6	-6.558743000	1.290273000	0.637081000
1	-5.058530000	2.694453000	0.078813000
6	-5.923463000	-1.064140000	0.637635000
1	-3.892646000	-1.477291000	0.070357000
6	-6.879061000	-0.060026000	0.837322000
1	-7.309952000	2.054663000	0.806243000
1	-6.186865000	-2.105369000	0.790802000
6	-2.615241000	2.363109000	-0.739044000
9	-3.531750000	2.925774000	-1.547056000
9	-1.444686000	2.441707000	-1.381655000
9	-2.528188000	3.101382000	0.384781000
1	-7.883569000	-0.330131000	1.151079000

Free carbene associated to 3-CF_3 - Singlet
Sum of electronic and zero-point Energies = -943.833668
Sum of electronic and thermal Free Energies = -943.875918

6	2.364242000	1.017960000	0.113229000
6	0.999072000	0.599513000	0.049028000
6	0.495352000	-0.733160000	0.062087000
6	0.058875000	1.661666000	0.005936000
6	-0.866326000	-0.972343000	0.042907000
1	1.185774000	-1.566548000	0.103491000
6	-1.305091000	1.421848000	-0.063431000
1	0.454953000	2.671886000	0.019497000
6	-1.761546000	0.103199000	-0.032542000
1	-1.248549000	-1.987493000	0.064109000
1	-2.014132000	2.240155000	-0.121371000
6	3.398619000	-0.058489000	-0.027128000
9	3.500047000	-0.641851000	1.194418000
9	4.602211000	0.435502000	-0.349511000
9	3.160377000	-1.047022000	-0.935105000
6	-3.246035000	-0.176089000	-0.015911000
9	-3.540102000	-1.311544000	-0.671926000
9	-3.686894000	-0.314930000	1.247805000
9	-3.946864000	0.819552000	-0.582434000

Free carbene associated to 3-CF_3 - Triplet
Sum of electronic and zero-point Energies = -943.852514
Sum of electronic and thermal Free Energies = -943.896116

6	-2.307130000	0.739494000	-0.079913000
6	-0.931866000	0.503261000	-0.041993000
6	-0.394218000	-0.816671000	-0.032372000
6	-0.023808000	1.597697000	-0.018099000
6	0.973483000	-1.014550000	0.006626000
1	-1.068954000	-1.664345000	-0.054990000
6	1.340863000	1.382252000	0.017733000
1	-0.418918000	2.608264000	-0.022164000
6	1.848370000	0.077581000	0.031506000
1	1.372773000	-2.023256000	0.022453000
1	2.023476000	2.224972000	0.044996000
6	-3.544604000	-0.051606000	0.002338000
9	-3.428635000	-1.234309000	-0.651288000
9	-4.577356000	0.611788000	-0.541189000
9	-3.876535000	-0.342072000	1.277384000
6	3.333701000	-0.149904000	0.009048000
9	3.670007000	-1.308612000	0.606179000
9	3.804832000	-0.205691000	-1.253738000
9	3.999119000	0.839901000	0.633814000

Free carbene associated to 3-OMe - Singlet
Sum of electronic and zero-point Energies = -721.385699
Sum of electronic and thermal Free Energies = -721.425536

6	1.669616000	1.060360000	-0.002652000
6	0.367107000	0.507120000	-0.003758000
6	-0.017921000	-0.874708000	-0.001292000
6	-0.687399000	1.462847000	-0.003863000
6	-1.338282000	-1.246284000	0.001033000
1	0.749283000	-1.640147000	-0.000665000
6	-2.021872000	1.106286000	-0.003092000
1	-0.389881000	2.506948000	-0.004662000
6	-2.352306000	-0.260540000	-0.000404000
1	-1.642465000	-2.288124000	0.003733000
1	-2.792558000	1.868088000	-0.003657000
6	2.784985000	0.057134000	-0.000835000
9	2.763007000	-0.722559000	1.116152000
9	3.999417000	0.634689000	-0.025217000
9	2.747002000	-0.771500000	-1.082767000
8	-3.603695000	-0.734060000	0.001520000
6	-4.692088000	0.182189000	0.001004000
1	-4.679403000	0.811023000	-0.895744000
1	-5.593034000	-0.430731000	0.002377000
1	-4.678244000	0.813339000	0.896107000

Free carbene associated to 3-OMe - Triplet
Sum of electronic and zero-point Energies = -721.393957
Sum of electronic and thermal Free Energies = -721.434663

6	-1.630846000	0.741561000	-0.097441000
6	-0.280561000	0.393675000	-0.063338000
6	0.154450000	-0.967236000	-0.060034000
6	0.722223000	1.398192000	-0.044380000
6	1.494895000	-1.279726000	-0.026687000
1	-0.585413000	-1.759237000	-0.085004000
6	2.070173000	1.078773000	-0.015849000
1	0.418016000	2.440210000	-0.049768000
6	2.469686000	-0.265401000	-0.004485000
1	1.827477000	-2.312946000	-0.020601000
1	2.802746000	1.877775000	-0.000195000
6	-2.921273000	0.055689000	0.012326000

9	-2.934017000	-1.116882000	-0.674512000
9	-3.918923000	0.815711000	-0.474400000
9	-3.242877000	-0.248982000	1.290928000
8	3.755007000	-0.688468000	0.025876000
6	4.781956000	0.285323000	0.049962000
1	4.715889000	0.918873000	0.943192000
1	5.720802000	-0.269010000	0.072547000
1	4.758555000	0.918355000	-0.845767000

Free carbene associated to 3-Ph - Singlet
Sum of electronic and zero-point Energies = -837.824209
Sum of electronic and thermal Free Energies = -837.867265

6	-3.006050000	0.920771000	-0.324167000
6	-1.632513000	0.581305000	-0.192738000
6	-1.063792000	-0.681256000	0.155286000
6	-0.724638000	1.636631000	-0.487105000
6	0.303400000	-0.858113000	0.192323000
1	-1.717752000	-1.512446000	0.389699000
6	0.644192000	1.467631000	-0.408757000
1	-1.160316000	2.586840000	-0.779902000
6	1.186053000	0.211644000	-0.075153000
1	0.713272000	-1.823542000	0.473875000
1	1.309868000	2.289178000	-0.654757000
6	-4.002515000	-0.122203000	0.076630000
9	-4.123487000	-0.974259000	-0.973334000
9	-5.215517000	0.404988000	0.315361000
9	-3.727367000	-0.879393000	1.181876000
6	2.646055000	0.013753000	-0.010628000
6	3.488037000	1.037719000	0.452949000
6	3.226477000	-1.200874000	-0.410703000
6	4.864305000	0.850485000	0.519517000
1	3.055823000	1.973306000	0.795640000
6	4.604043000	-1.382363000	-0.353771000
1	2.596406000	-1.994328000	-0.801678000
6	5.427311000	-0.358952000	0.114028000
1	5.498491000	1.648479000	0.894470000
1	5.036687000	-2.323255000	-0.681115000
1	6.502683000	-0.503321000	0.162378000

Free carbene associated to 3-Ph – Triplet
Sum of electronic and zero-point Energies = -837.838582
Sum of electronic and thermal Free Energies = -837.883032

6	-2.952043000	0.722064000	-0.163690000
6	-1.571949000	0.538836000	-0.120038000
6	-0.992008000	-0.710217000	0.250692000
6	-0.687335000	1.604862000	-0.443384000
6	0.379508000	-0.866787000	0.279787000
1	-1.640540000	-1.535817000	0.523084000
6	0.680993000	1.427586000	-0.401955000
1	-1.106402000	2.562196000	-0.737084000
6	1.253024000	0.190559000	-0.043237000
1	0.793571000	-1.821558000	0.591164000
1	1.330345000	2.251741000	-0.682573000
6	-4.138816000	-0.121260000	0.031625000
9	-4.278937000	-1.044819000	-0.947023000
9	-5.260614000	0.617830000	0.050023000
9	-4.083356000	-0.807269000	1.200284000
6	2.717215000	0.008809000	-0.006079000
6	3.563109000	1.050373000	0.408151000
6	3.302961000	-1.210023000	-0.384806000
6	4.943198000	0.879093000	0.443015000
1	3.131102000	1.991991000	0.735166000
6	4.683261000	-1.380059000	-0.351233000
1	2.671407000	-2.020068000	-0.738487000
6	5.509859000	-0.336688000	0.063062000
1	5.577845000	1.695197000	0.776919000
1	5.115504000	-2.328136000	-0.658856000
1	6.587467000	-0.470118000	0.089645000
